# Multimorbidity and the risk of post-tuberculosis lung disease: a systematic review and meta-analysis

**DOI:** 10.1136/bmjgh-2025-020365

**Published:** 2026-06-01

**Authors:** Katherine Jane Hill, Irene Mbabazi, Marcello Sergio Scopazzini, Benedict Warner, Christine Sekaggya-Wiltshire, Sarah Mills, Stellah Mpagama, Helen Ruth Stagg, Derek James Sloan

**Affiliations:** 1 Infection and Global Health, University of St Andrews School of Medicine, St Andrews, UK; 2 Research Department, Infectious Diseases Institute - Makerere University, Kampala, Uganda; 3 Department of Infectious Disease Epidemiology, London School of Hygiene & Tropical Medicine, London, UK; 4 School of Health and Wellbeing, University of Glasgow, Glasgow, UK; 5 Kibong’oto Infectious Diseases Hospital, Sanya Juu, Tanzania

**Keywords:** Tuberculosis, HIV, Nutrition, Diabetes, Global Health

## Abstract

**Introduction:**

Post-tuberculosis lung disease (PTLD), a chronic respiratory syndrome affecting approximately half of tuberculosis (TB) survivors, is an emerging public health threat in low- and middle-income countries (LMICs). Multimorbidity, defined as two or more coexisting chronic health conditions, is also rising in LMICs. This systematic review and meta-analysis examines associations between multimorbidity or individual comorbidities and PTLD.

**Methods:**

Medline, EMBASE, Web of Science, Global Health and Scopus databases up to 7 March 2024 were searched and studies in LMICs assessing PTLD and either a single comorbidity or multimorbidity were included. Summary data were extracted independently by two reviewers. Random effects meta-analyses were performed where sufficient comparable data were reported. Risk of bias was assessed using Risk Of Bias in Non-randomised Studies-of Exposure and certainty of evidence for meta-analysable associations was assessed using Grading of Recommendations Assessment, Development and Evaluation. Study protocol was registered with PROSPERO (CRD42024552486).

**Results:**

41 papers, from 10 321 screened, were included. None analysed multimorbidity. PTLD measurement tools varied widely. Meta-analysis of 10 of 24 papers analysing the association between spirometry-defined PTLD and HIV status found reduced odds of PTLD in people living with HIV (OR 0.68, 95% CI 0.52 to 0.89). 15 papers reported on diabetes and showed inconsistent associations; meta-analysis of three papers yielded an OR of 1.65 (95% CI 0.96 to 2.84). Adults with undernutrition had increased odds of abnormal spirometry following TB on meta-analysis of three studies (OR 1.99, 95% CI 1.02 to 3.87). Evidence for other comorbidities was limited and inconclusive. Overall, 24/41 (58.5%) of included studies were at high risk of bias and certainty of evidence for meta-analysed associations was very low.

**Conclusion:**

PTLD may be more common among adults who are HIV-negative or undernourished, but the evidence is very uncertain. The absence of dedicated multimorbidity studies and inconsistencies in defining PTLD highlight the need for prospective studies evaluating comorbidities in relation to PTLD.

**PROSPERO registration number:**

CRD42024552486.

**WHAT IS ALREADY KNOWN ON THIS TOPIC**

- Approximately half of tuberculosis (TB) survivors develop chronic respiratory abnormalities—post-TB lung disease (PTLD)—following TB treatment.
- It is recognised that an assessment of comorbidities which may influence outcomes is an important aspect of clinical care, but it is not clear which comorbidities matter most or how the coexistence of multiple long-term conditions (termed multimorbidity) influences PTLD.

**WHAT THIS STUDY ADDS**

- Careful analysis of the associations between several comorbidities and PTLD has revealed the high risk of bias in current evidence used to evaluate these relationships.
- There may be increased odds of PTLD in adults who are HIV-negative or undernourished, but the evidence is very uncertain.
- There is inconsistency in PTLD assessment methods and definitions currently in use in research, which makes evidence synthesis challenging.

**HOW THIS STUDY MIGHT AFFECT RESEARCH, PRACTICE OR POLICY**

- This review highlights a critical need for better standardised, high-quality, prospective studies delineating the relationship between comorbidities and multimorbidity and risk of PTLD.

## Introduction

 Over 10 million people globally fall ill with tuberculosis (TB) every year and it remains a leading cause of death worldwide.^1^ The focus on TB diagnostics and treatment has resulted in more patients surviving and a move towards the WHO’s end TB targets.^2^ However, the immune responses that help clear infection can lead to fibrotic granuloma and cavity formation, leaving survivors with chronic lung damage following treatment.^3 4^ This clinical syndrome has been coined ‘post-TB lung disease (PTLD)’ and affects over 40% of TB survivors following treatment.^5^ As WHO reports end of TB treatment (EOT) outcomes without considering post-treatment events, reports of successful treatment in 85% of individuals masks the impact of TB on global morbidity.^1 6^

At the first international PTLD symposium ‘evidence of chronic respiratory abnormality, with or without symptoms, attributable at least in part to previous tuberculosis’ was chosen as the standard PTLD definition.^7^ This recognises that lung damage may be asymptomatic but still relevant to patient outcomes, and patients without previously documented TB may still suffer from PTLD. At this symposium, a toolbox of PTLD measurements was suggested, which included an assessment of comorbidities that may influence disease or outcomes. However, it is unclear which comorbidities affect the risk of developing PTLD, and how the coexistence of two or more long-term conditions (LTCs)—termed multimorbidity by the Academy of Medical Sciences^8^—may modify this risk. Prior reviews have summarised risk factors for PTLD, reporting associations with age, HIV status, smear-positivity, smoking, alcohol use, radiological abnormality and prior TB.^5 9^ However, these reviews did not search specifically for or evaluate studies focused on single or multiple LTCs and did not assess the methodological quality of included studies in depth. As non-communicable disease prevalence in low- and middle-income countries (LMICs) increases, coinciding with an ongoing high TB incidence, this knowledge gap needs to be addressed urgently.^10 11^

We examined whether multimorbidity, defined as two or more LTCs in addition to TB, increases the risk of developing PTLD among people treated for TB in LMICs. Our secondary objective was to examine the effect of individual comorbidities on PTLD risk. We focused on LMICs to reflect the epidemiology and management of LTCs, and the socio-economic determinants of chronic lung disease, in countries which account for 99% of people newly diagnosed with TB each year.^1^

## Methods

### Search strategy and selection criteria

We systematically reviewed, and meta-analysed where possible, studies evaluating the association between PTLD and either multimorbidity or single comorbidities. Medline, EMBASE, Web of Science, Global Health and Scopus databases were searched using search terms for multimorbidity and comorbidities, PTLD and pulmonary disease/assessment (online supplemental table S1). Papers from database inception until the date of the search, 7 March 2024, were included. The search was not restricted by study location or publication language. The search strategy was developed with guidance from a medical librarian.

Studies evaluating (1) participants of any age with a diagnosis of TB involving the respiratory system in which (2) an assessment of PTLD had occurred, using one or more of the recommended assessment tools (online supplemental table S2)^7^ and presenting a description of (3) either multimorbidity or a single comorbidity, defined as any morbidity additional to TB and guided by Ho *et al*,^12^ in the study population were included. To be most representative of evidence to date, studies that analysed variables used for standard definitions of comorbidities continuously were also included. Studies from countries with a World Bank classification of high-income in the year the study was conducted were excluded. Case reports, case series, reviews, qualitative studies and editorials were excluded. Full details of inclusion and exclusion criteria are in online supplemental table S3.

The results of the database searches were de-duplicated automatically, using Endnote and Covidence software, and manually. Titles and abstracts of papers, followed by full texts, were screened within the Covidence software. All screening, full-text reviews, data extraction and risk of bias assessments were performed independently by the primary reviewer—KJH—and a second reviewer—BW, IM or MSS. Disagreements at any stage of the screening or extraction were resolved by consensus. Studies not in English were reviewed and data extracted alongside a human translator. A core patient and public involvement group of three TB survivors were consulted to guide research question design and contextualise the experience of TB treatment alongside comorbidities in an LMIC. The review was conducted in accordance with Preferred Reporting Items for Systematic Reviews and Meta-Analyses (PRISMA) guidelines. The protocol was registered with PROSPERO before data extraction (https://www.crd.york.ac.uk/PROSPERO/view/CRD42024552486).

### Data analysis

Data inclusive of study design, participant demographics, TB diagnosis method, PTLD assessment method, definition of multimorbidity and comorbidities, ORs or risk ratios for PTLD for multimorbidity or comorbidities and details of any adjustment were extracted. Several comorbidities were measured in different ways across studies. Undernutrition, identified by the WHO as a key TB comorbidity, and anaemia are common in TB and consistently associated with disease severity and treatment outcomes.^13^,^16^ Nutritional status is also a modifiable determinant of TB risk: in the “Reducing Activation of Tuberculosis by Improvement of Nutritional Status (RATIONS)” cluster-randomised trial, household nutritional supplementation reduced TB incidence.^17^ We, therefore, extracted body mass index (BMI) and haemoglobin where reported and analysed them as comorbidities. Studies which reported BMI or haemoglobin as continuous variables were analysed separately from studies which reported them categorically.

The risk of bias of included papers were assessed using the Risk Of Bias in Non-randomised Studies-of Exposure tool.^18^ Age, smoking and socio-economic status were identified as the minimum confounder set to be adjusted for.

Papers were grouped according to what comorbidity, defined as a single LTC comorbid to TB, was analysed. Papers which analysed two or more LTCs in addition to TB were categorised as multimorbidity studies. Papers were also grouped according to which PTLD assessment tool was used. Descriptive statistics were used to present data. ORs, if not reported, were calculated where possible. Random effects meta-analyses were undertaken when three or more papers met the following criteria: multimorbidity or comorbidity defined uniformly, PTLD measured and defined uniformly, and ORs available. Papers were further subgrouped according to presence or absence of adjustment for the minimum confounder set. The proportion of total variability due to interstudy heterogeneity was calculated using the I^2^ statistic. Publication bias was assessed using funnel plots and Egger’s test. Where there were enough studies for meta-analysis, the certainty of evidence for the association was assessed by two reviewers (KJH and BW) using the Grading of Recommendations Assessment, Development and Evaluation (GRADE) framework, as described in online supplemental table S4.^19^ Discrepancies were resolved by consensus. RStudio V.4.4.1 was used for all analyses, code given in online supplemental table S5.

## Results

**Figure 1:**
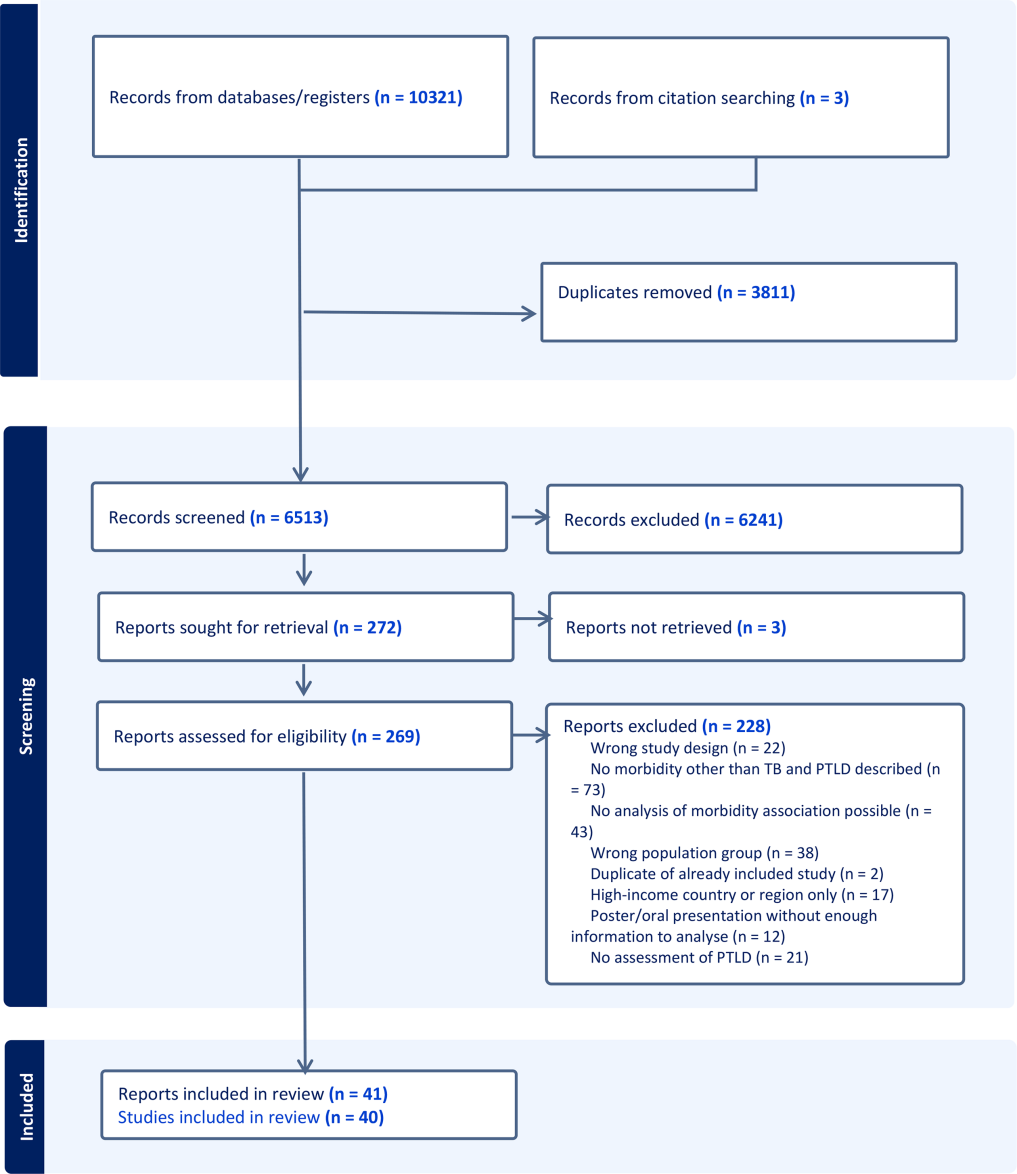
PRISMA flow diagram. PRISMA, Preferred Reporting Items for Systematic Reviews and Meta-Analyses.

Our search strategy identified 10 321 papers and a further three papers were identified through screening the references of included papers. After de-duplication and title and abstract screening, 272 papers were eligible for full-text review (figure 1). Of these, 41 papers from 40 studies were retained (table 1 and online supplemental table S6).^20^,^60^ Inter-rater reliability was 90–97% at title and abstract screening and 80–94% at full-text review. Reasons for exclusion following full-text review are summarised in online supplemental table S14.

**Table 1:** Summary of included papers

Paper name	Study type	Country	Sample size*	Time point of comorbidity assessment	Comorbidities measured (Number: Which)	Time point of PTLD assessment	PTLD assessment tool used in analysis	Risk of bias Full results shown in online supplemental table S4
Allwood *et al*^20^	Cross-sectional	South Africa	100	At PTLD assessment	3: N, HIV, Htn	EOT	Echocardiography	Very high
Allwood *et al*^21^	Cross-sectional	South Africa	107	At PTLD assessment	5: N, Al, Dr, HIV, RD	≤5 years after EOT	Spirometry and 6MWT	Very high
Andrea and Faisal^22^	Cross-sectional	Malaysia	82	At PTLD assessment	1: D	≤5 years after EOT	Spirometry	Very high
Auld *et al*^23^	Prospective cohort	South Africa	92	TB diagnosis	1: D	≤1 year after EOT	Spirometry	Very high
Chin *et al*^24^	Prospective cohort	Zimbabwe	175	During treatment	2: HIV, MH	Variable	Composite	High
Gandhi *et al*^25^	Cross-sectional†	India	146	At PTLD assessment	1: N	EOT	Spirometry	Very high
Gupte *et al*^26^	Prospective cohort	India	172	TB diagnosis	4: N, Al, HIV, D	≤1 year after EOT	Spirometry	Some concerns
Gupte *et al*^27^	Prospective cohort	India	377	TB diagnosis	3: N, HIV, D	EOT	SGRQ	Very high
Khosa *et al*^28^	Prospective cohort	Mozambique	62	TB diagnosis	4: N, Al, HIV, An	≤1 year after EOT	Spirometry	Very high
Kumar Rai and Kumar^29^	Prospective cohort	India	128	TB diagnosis	1: D	EOT	CXR	Very high
Lin *et al*^30^	Cross-sectional	China	115	At PTLD assessment	1: D	EOT	Symptoms, CXR and 6MWT	Very high
Lisha *et al*^31^	Cross-sectional	India	224	TB diagnosis and PTLD assessment	1: D	≤5 years after EOT	CXR	Very high
Louw *et al*^32^	Cross-sectional	South Africa	100	At PTLD assessment	4: HIV, D, Htn, RD	≤5 years after EOT	Echocardiography	Some concerns
Mancuzo *et al*^33^	Cross-sectional	Brazil	378	At PTLD assessment	8: Al, HIV, D, Htn, RD, K, Ma, Cv	≤5 years after EOT	Spirometry	High
Manji *et al*^34^	Cross-sectional	Tanzania	501	TB diagnosis	1: HIV	EOT‡	Spirometry	High
Mbatchou Ngahane *et al*^35^	Cross-sectional	Cameroon	269	TB diagnosis	2: N, HIV	≤5 years after EOT	Spirometry	Very high
Meghji *et al*^36^§	Prospective cohort	Malawi	368	TB treatment completion	3: N, HIV, An	≤1 year after EOT	Spirometry and symptoms	Some concerns
Mily *et al*^37^	Case-control	Bangladesh	71	TB diagnosis	1: D	EOT	CXR	High
Mpagama *et al*^38^	Cross-sectional	Tanzania	219	Unclear	3: Al, Dr, HIV	≤5 years after EOT	Composite¶	Some concerns
Mugo^39^	Cross-sectional	Kenya	183	At PTLD assessment	2: N, HIV	≤5 years after EOT	Spirometry	High
Namusobya *et al*^40^	Cross-sectional	Uganda	326	At PTLD assessment	2: Al, I	≤5 years after EOT	Symptoms	High
Nightingale *et al*^41^§	Prospective cohort	Malawi	301	TB treatment completion	3: N, HIV, RD	≤5 years after EOT	Spirometry	Some concerns
Nihues *et al*^42^	Cross-sectional	Brazil	121	At PTLD assessment	4: N, Al, Dr, RD	1–12 years after EOT	Symptoms	High
Nkereuwem *et al*^43^	Cross-sectional	The Gambia	79	At PTLD assessment	2: N, HIV	EOT	Spirometry	High
Nuwagira *et al*^44^	Cross-sectional	Uganda	95	At PTLD assessment	1: HIV	≤5 years after EOT	Spirometry	High
Osman *et al*^45^	Cross-sectional	South Africa	51	TB treatment completion	1: HIV	≤5 years after EOT	Symptoms	Very high
Page *et al*^46^	Prospective cohort	Uganda	284	Unclear	1: HIV	≤10 years after EOT	Chronic pulmonary aspergillosis**	Very high
Perfura-Yone *et al*^47^	Cross-sectional	Cameroon	177	TB treatment completion	1: HIV	EOT	Symptoms	Very high
Pydipalli *et al*^48^	Cross-sectional	India	118	At PTLD assessment	3: Al, D, HTN	≤1 year after EOT	Spirometry	Very high
Ralph *et al*^49^	Prospective cohort	Indonesia	200	TB diagnosis	1: HIV	EOT	SGRQ and 6MWT	Some concerns
Ross *et al*^50^	Retrospective cohort	South Africa	185	TB diagnosis	1: HIV	≤5 years after EOT	Spirometry	Some concerns
Salzer *et al*^51^	Prospective cohort	Mozambique	20	TB diagnosis	1: HIV	EOT	CXR and Aspergillus IgG	Very high
Santamaria-Alza *et al*^52^	Retrospective cohort	Columbia	141	TB diagnosis and PTLD assessment	2: D, Dr	Variable	Imaging (CXR or CT)	Very high
Soemarwoto *et al*^53^	Case–control	Indonesia	64	Not stated	Multiple††	Not stated	Not stated	Very high
Swaminathan *et al*^54^	Prospective cohort	India	162	TB diagnosis	1: HIV	EOT	CXR	Very high
Tandon *et al*^55^	Retrospective cohort	India	60	TB diagnosis	6: Al, D, HTN, An, K, Ma	EOT	CXR	Very high
Vashakidze *et al*^56^	Cross-sectional	Georgia	58	At PTLD assessment	3: N, Al, HCV	≤5 years after EOT	Spirometry and SGRQ	High
Wu *et al*^57^	Prospective cohort	China	71	TB diagnosis	1: D	EOT	HRCT and Symptoms	Very high
Wu *et al*^58^	Cross-sectional	China	975	At PTLD assessment	3: Al, RD, Me	≤5 years after EOT	CAT	Very high
Zawedde *et al*^59^	Cross-sectional	Uganda	162	TB diagnosis	1: HIV	EOT	Spirometry	Very high
Zubair *et al*^60^	Retrospective cohort	Pakistan	321	Not stated	1: D	EOT	CXR	Very high

* Number of participants included in analysis.

† Reported as case–control but appears cross-sectional.

‡ Participants enrolled into study after 20 weeks of anti-TB treatment.

§ Nightingale is the same cohort as Meghji, assessed 2 years later.

¶ Symptoms plus either abnormal spirometry or CXR.

** Based on symptoms, CXR changes and positive aspergillus IgG.

†† Definition of ‘multiple diseases’—likely TB or destroyed lung accompanied by ≥1 other disease versus none—did not fit our definition of multimorbidity.

AI, alcohol use disorder; An, anaemia; CAT, chronic obstructive pulmonary disease assessment test; Cv, cardiovascular disease; CXR, chest X-ray; D, diabetes mellitus; Dr, recreational drug use; EOT, end of TB treatment; HCV, chronic hepatitis C infection; HRCT, high-resolution CT; Htn, hypertension; I, immunosuppressive conditions other than HIV; K, chronic kidney disease; Ma, malignancy; Me, ‘chronic metabolic disorder’; MH, mental health condition; 6MWT, 6-minute walk test; N, nutritional status; PTLD, post-TB lung disease; RD, chronic respiratory disease; SGRQ, St George’s Respiratory Questionnaire; TB, tuberculosis.

**Figure 2:**
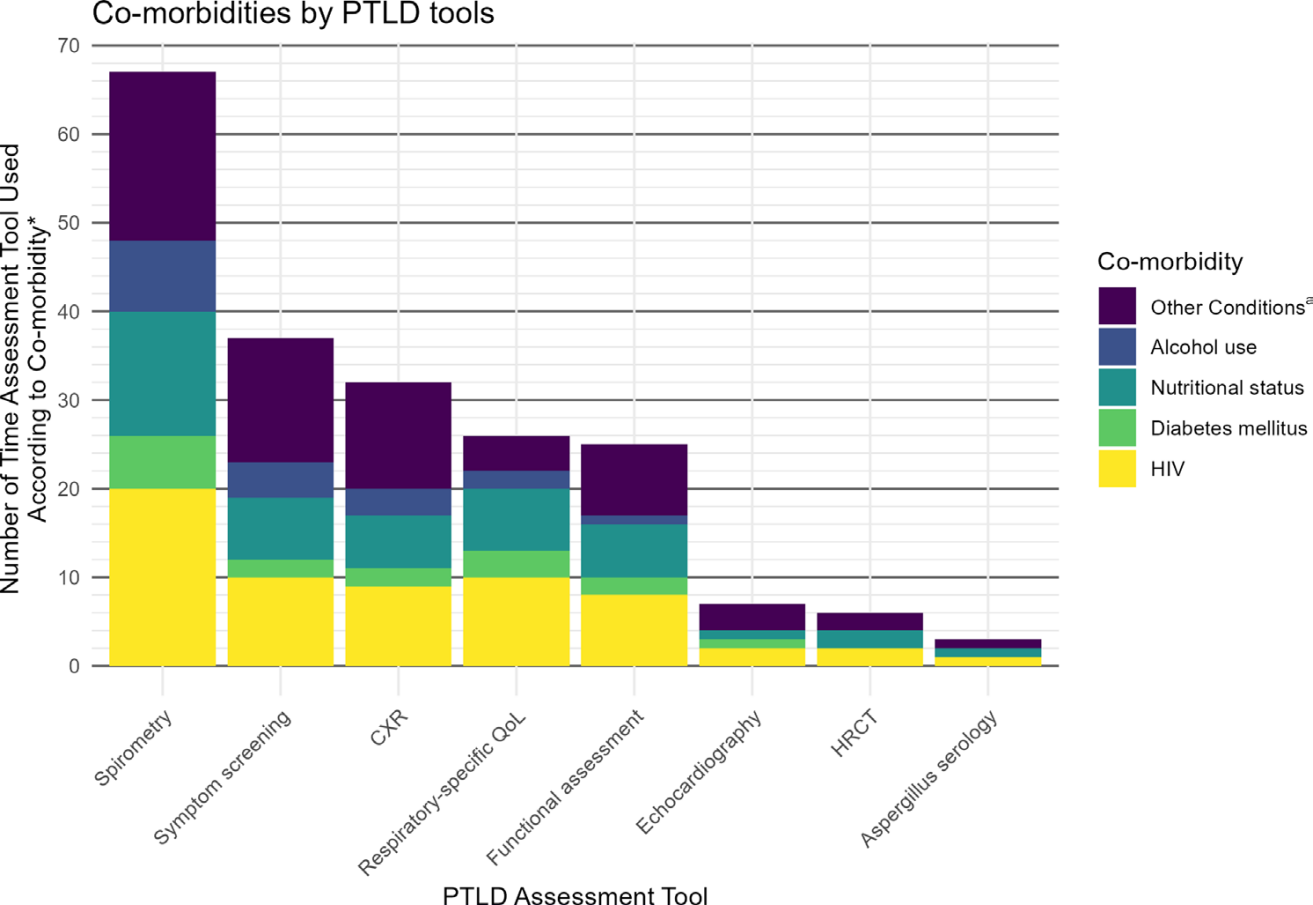
PTLD assessment tools used in included papers. *This represents each time a PTLD assessment tool is used to assess the association between the results of that assessment tool and the specified comorbidity. Some papers assess PTLD using multiple tools, and according to multiple comorbidities. ^a^Other conditions include recreational drug use, pre-existing respiratory disease, hypertension, anaemia, cardiac disease, chronic kidney disease, malignancy, mental health, hepatitis C and immunosuppression other than HIV.

Included papers were predominantly cross-sectional (22/41, 53.7%) and from Africa (21/41, 51.2%, online supplemental figure S1). All papers studied adults apart from Nkereuwem *et al* which assessed children and adolescents only.^43^ Only two papers restricted to drug-resistant TB. There was little consistency in PTLD assessment timing or method. Spirometry, chronic respiratory symptom screening and chest X-ray (CXR) were most used (figure 2). The reported prevalence of PTLD ranged from 13–91%. 24/41 (58.5%) of the included papers were deemed to be at very high risk of bias due to uncontrolled confounding in their analyses of the association between the comorbidity and PTLD (online supplemental table S7). The remainder were either high risk (n=10, 23.8%) or some concerns (n=7, 16.7%).

No papers reported the effect of multimorbidity on risk of PTLD, despite 22 papers measuring and analysing more than one LTC. The following paragraphs look at individual comorbidities, further sub-grouped by the PTLD measurement used due to substantial variation between papers.

### HIV

**Figure 3:**
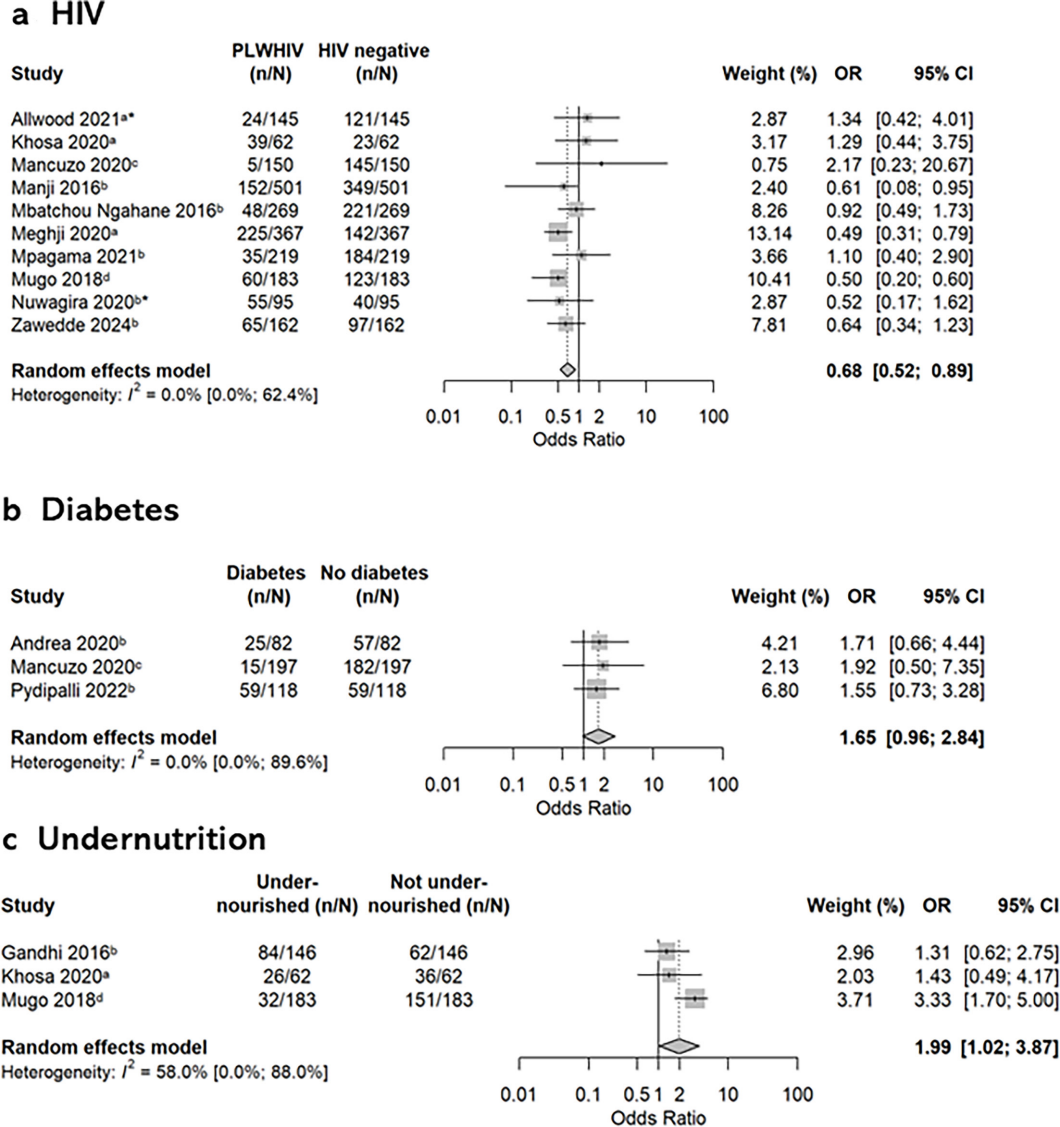
Forest plots of meta-analyses of the association between PTLD and HIV, diabetes mellitus and undernutrition. I^2^ reported as value (95% CI). ^a^Airway obstruction=FEV1/FVC <LLN or Z-score<−1.64, restrictive defect=FVC <lower limit of normal or Z-score<−1.64. ^b^Airway obstruction=FEV1/FVC <0.70, restrictive defect=FVC <0.80 predicted. ^c^Moderate/severe airway obstruction=FEV1/FVC<0.60, moderate/severe restrictive defect=FVC <0.60 predicted. ^d^Cut-offs not specified. *Only airway obstruction reported. FEV1, forced expiratory volume in 1 s; FVC, forced vital capacity; LLN, lower limit of normal; PLWHIV, people living with HIV; PTLD, post-tuberculosis lung disease.

24 papers assessed the risk of PTLD according to HIV status (online supplemental table S8).^1112 15 17^,^19 23^ PTLD assessments were conducted at EOT in nine papers and up to 10 years following EOT in the remainder. Most papers (17/24) were at high or very high risk of bias. HIV prevalence ranged from 4.0% to 65.6%. 14 papers assessed PTLD using spirometry.^2123 26 28 33^,^36 39 41 43 44 50 59^ 10 of these (71%), which categorised lung function as abnormal or not based on forced expiratory volume in 1 s (FEV1)/forced vital capacity (FVC) and FVC results and had extractable ORs, were included in a meta-analysis (figure 3). From this, there were reduced odds of developing PTLD if coinfected with HIV (pooled OR 0.68, 95% CI 0.52 to 0.89). I^2^ was 0% but with a wide uncertainty interval (95% CI 0.0 to 62.4%). Sensitivity analysis grouping the studies by whether they adjusted for the minimum confounder set found a lower OR among those that did (OR 0.59, 95% CI 0.39 to 0.90), compared with those that did not (OR 0.77, 95% CI 0.52 to 1.13) (online supplemental figure S2), demonstrating the importance of adjusted results. No clear evidence of publication bias was seen on visual inspection of the funnel plot (online supplemental figure S3). Egger’s test could not rule out publication bias (intercept 1.63, 95% CI 0.38 to 2.89) but this result is influenced by Mancuzo *et al* which had few participants with both HIV and PTLD, a relatively large effect size and reported an unadjusted OR (uOR).^33^ GRADE assessment of the association between HIV and abnormal spirometry in papers included in the meta-analysis was downgraded due to limitations in risk of bias (8/10 papers at high or very high risk), inconsistency and publication bias domains, resulting in very low certainty in this result (online supplemental table S13).

The results from the remaining four adult spirometry papers were mixed.^26 41 43 50^ Nightingale *et al* found people living with HIV (PLWHIV) had better lung function at 3 years following TB; FEV1 and FVC were 0.19 L (95% CI 0.06 to 0.31) and 0.18 L (95% CI 0.03 to 0.32) higher respectively in PLWHIV.^41^ Two papers reported no demonstrable difference in absolute FEV1 or FVC.^26 50^ In children and adolescents, Nkereuwem *et al* did not demonstrate a difference in PTLD according to HIV status, but this assessment was unadjusted (uOR 0.9, 95% CI 0.8 to 1.1).^43^

The reported results in papers which assessed the association of HIV status with PTLD using symptomology (three papers),^36 45 47^ 6-minute walk test (6MWT) (two papers),^21 49^ St George’s Respiratory Questionnaire (SGRQ) (two papers)^27 49^ or CXR (two papers)^51 54^ were variable (table 2). No evidence of an association was seen in the two papers which assessed for pulmonary hypertension using echocardiography,^20 32^ or in the one paper which used a composite measure of radiology, symptoms, spirometry and exercise testing.^24^

**Table 2:** Summary of associations between comorbidities and PTLD

Comorbidity (No. of papers)	Results	Risk of bias*
HIV (24†)^2021 24 26^,^28 32^	Spirometry (14 papers): HIV may reduce the odds of abnormal spirometry, but the evidence is very uncertain. See results section for details.	●●●●●●●● ●●●●●●
	Symptoms (three papers): PLWHIV less likely to have ongoing respiratory symptoms at 1 year in one paper,^36^ no evidence of an association in two papers.^45 47^	●●●
	6MWT (two papers): PLWHIV walked 49.55 m further in one paper (95% CI 9.31 to 89.79, p value 0.02),^21^ no evidence of an association in the other.^49^	●●
	SGRQ (two papers): PLWHIV had worse SGRQ scores in one paper,^49^ no difference in the other.^27^	●●
	CXR (two papers): reduced odds of sequelae in PLWHIV in one paper (uOR 0.02 (95% CI 0.00 to 0.12)),^54^ other study too small to draw any conclusions.^51^	●●
	Echocardiography (two papers): no evidence of an association with PH.^20 32^ Only 56 PLWHIV in total.	●●
	Composite measure (one paper): no evidence of an association (aRRR=0.6 (95% CI 0.3 to 1.3))^24^	●
Diabetes mellitus (15) 2223 26 27 29,33 37 48 52 55 57 60	Spirometry (five papers): direction of effect towards increased odds of PTLD in diabetes in papers included in meta-analysis, but CIs cross the null and evidence is very uncertain.	●●●●●
	Symptoms (two papers): inconsistency in results—increased respiratory symptoms in diabetes in one paper (p value 0.027),^30^ no difference found in the other paper.^57^	●●
	Radiological imaging (eight papers): increased risk of consolidation and cavitation but reduced risk of bronchiectasis at EOT in one paper.^57^ No difference found in other seven papers.^29^,^3137 52 55 60^	●●●●●●●●
	Other measures: no evidence of an association found when PTLD assessed by SGRQ (one paper),^27^ 6MWT (one paper)^30^ or echocardiography (one paper)^32^	●●●
Nutritional status (13‡)^2021 25^,^28 35 36 39 41^	**Continuous BMI**	
	Spirometry (four papers): worsening lung function with reducing BMI in three papers,^21 36 41^ reducing FEV1 and FVC (% predicted) but increasing FEV1/FVC with increasing BMI in the other.^56^	●●●●
	6MWT (one paper): distance decreased as BMI increased but few participants were undernourished.^21^	●
	Others: no evidence of an association with persistent symptoms (two papers)^36 42^ or pulmonary hypertension (one paper).^20^	●●
	**Undernutrition**	
	Spirometry (five papers): malnutrition may increase the odds of abnormal spirometry, but the evidence is very uncertain. See results section for details.	●●●●●
	SGRQ (one paper): higher (worse) median scores in persons with undernutrition^27^	●
	**Overweight/obesity**	
	Spirometry (three papers): reduced odds of abnormal spirometry if overweight in one paper,^25^ in the other two there was no evidence of an association between obesity^35^ or being overweight.^39^	●●●
AUD (7)^26 28 33 40 42 55 58^	Spirometry (three papers): no overall evidence of an association. Only 168 participants in total with AUD.	●●●
	Symptoms (two papers): inconsistent results, see results section for detail.	● ●
	Others: no evidence of an association found when PTLD assessed by CXR (one paper) or CAT (one paper).	●●
Recreational drug use (4)^21 38 42 52^	Radiological sequelae seen more frequently, but not quantified, in one paper.^52^ Otherwise, no clear association found using any assessment tool. Only 150 total patients with recreational drug use, which was not uniformly defined.	●●●●
Respiratory disease (6)^21 32 33 41 42 58^	Spirometry (three papers): increased odds of abnormal spirometry in one paper,^33^ no evidence of an association in other two papers.^21 41^	●●●
	Others: patients with prior respiratory disease more likely to have chronic respiratory symptoms,^42^ and worse CAT scores following TB.^58^ No evidence of an association with worse 6MWT results or PH assessed by echocardiography.	●●●●
Hypertension (5)^20 32 33 48 55^	No evidence of an association demonstrated with abnormal spirometry,^33 48^ CXR abnormalities^55^ or PH assessed by echocardiography.^20 32^ Only 152 participants in total with hypertension.	●●●●●
Anaemia and Hb (3)^28 36 55^	No evidence of an association between anaemia at EOT and CXR sequelae.^55^ Baseline Hb: relative risk of both mild and moderate-severe lung impairment on spirometry reduced with increased baseline Hb in one study.^28^ EOT Hb: increased Hb associated with persistent respiratory symptoms.^36^	●●●
Cardiac disease (1)^33^	16 patients had cardiac disease, none of whom had abnormal spirometry.	●
CKD (2)^33 55^	No evidence of an association demonstrated. Only 24 participants in total had CKD.	●●
Malignancy (2)^33 55^	No evidence of an association demonstrated. Only 11 participants in total had malignancy.	●●
Mental health (1)^24^	No evidence of an association demonstrated. Only 29 participants with a mental health condition.	●
Hepatitis C (1)^56^	Hepatitis C, present in four participants, was associated with worse spirometry results (FEV1 % predicted, FVC % predicted and FEV1/FVC) and SGRQ scores. However, p value was between 0.01 and 0.05 for FEV1/FVC and otherwise all above 0.05. No 95% CI reported.	●
‘Chronic Metabolic Disorder’ (1)^58^	No evidence of an association with persistent respiratory symptoms (uOR 1.33, 95% CI 0.91 to 1.95). ‘Chronic metabolic disorder’ not further defined.	●
Immunosuppressive conditions§ (1)^40^	Increased odds of PTLD symptoms (aOR 7.72, 95% CI 3.13 to 19.04). Included conditions were predominantly diabetes and chronic steroid use.	●

* Risk of bias of each paper in the association summary is illustrated with a coloured dot; ● = very high, ● = high, ● = some concerns and ● = low risk.

† From 23 studies.

‡ From 12 studies.

§ Other than HIV.

aOR, adjusted OR; aRRR, adjusted relative risk ratio; AUD, alcohol use disorder; BMI, body mass index; CAT, Chronic Obstructive Pulmonary Disease Assessment Tool; CKD, chronic kidney disease; CXR, chest X-ray; EOT, end of treatment; FEV1, forced expiratory volume in 1 s; FVC, forced vital capacity; Hb, serum haemoglobin; 6MWT, 6-minute walk test; PH, pulmonary hypertension; PLWHIV, people living with HIV; PTLD, post-tuberculosis lung disease; SGRQ, St George’s Respiratory Questionnaire; TB, tuberculosis; uOR, unadjusted OR.

### Diabetes mellitus

15 papers assessed the association of diabetes with PTLD (online supplemental table S9).^2223 26 27 29^,^33 37 48 52 55 57 60^ The prevalence of diabetes ranged from 5% to 50%. PTLD assessments were conducted at EOT in seven papers, at a mean of 69.3 months (±103.4 SD) in one,^52^ and up to 5 years after EOT in the remainder. In contrast to the papers looking at HIV in which 20 (80%) of papers were from Africa, 10 (67%) of diabetes papers were from South-East Asia with only 2 papers (13%) from Africa. Three papers assessing PTLD by spirometry were meta-analysed (figure 3).^22 33 48^ While there was a direction of effect towards increased odds of PTLD in patients with diabetes, the overall estimate was imprecise and CIs crossed the null (OR 1.65, 95% CI 0.96 to 2.84). Use of Egger’s test to assess publication bias was not possible and the funnel plot (online supplemental figure S4) should be interpreted with caution due to fewer than 10 included studies. None of the included papers adjusted for confounding. The certainty of evidence was rated as very low using GRADE, owing to risk of bias (all papers were at high or very high risk), inconsistency and imprecision (online supplemental table S13). Auld *et al* did not find evidence of an association between baseline glycated haemoglobin (HbA1c) and abnormal spirometry at 12 months (uOR 1.13, 95% CI 0.76 to 1.69, HbA1c categorised into <5.7, 5.7–5.9, 6.0–6.2, ≥6.3). Gupte *et al* reported variance in the effect of diabetes by spirometry pattern of disease (online supplemental figure S5).^26^ One paper found increased post-TB respiratory symptoms with diabetes (p=0.027)^30^ while another found no evidence of an association.^57^ Eight papers used radiological imaging to assess for PTLD: CXR (six), high-resolution CT (one) or either (one).^29^,^3137 52 55 57 60^ Diabetes was associated with increased risk of consolidation and cavitation but reduced risk of bronchiectasis in Wu *et al.*^57^ The remaining seven radiological imaging papers did not find any difference according to diabetes status.^29^,^3137 52 55 60^ There were few participants with diabetes in these papers and all but two papers were at high or very high risk of bias. No evidence of an association was found between diabetes and PTLD assessed by SGRQ,^27^ 6MWT^30^ or echocardiography.^32^

### Nutritional status

13 papers assessed the association of nutritional status with PTLD (online supplemental table S10); undernutrition (defined as BMI <18.5 kg/m^2^) in six papers,^25^,^2839 43^ overweight (BMI ≥25.0 kg/m^2^) or obesity (BMI ≥30.0 kg/m^2^) in three papers^25 35 39^ and BMI as a continuous variable in six papers.^20 21 36 41 42 56^ PTLD assessments were conducted at EOT in four papers and up to 12 years after EOT in the remainder. Studies had some concern (three papers), high (four papers) or very high risk of bias (six papers). Three papers reporting ORs for the association between undernutrition in adults, measured either at TB diagnosis^28^ or time of PTLD assessment,^25 39^ and abnormal spirometry were included in a meta-analysis (figure 3 and online supplemental table S6). Odds of abnormal spirometry increased with undernutrition (pooled OR 1.99, 95% CI 1.02 to 3.87) but with very low certainty on GRADE assessment due to very serious limitations in risk of bias and inconsistency (I^2^=58.0%, 95% CI 0.0% to 88.0%) (online supplemental table S13). In Nkereuwem *et al*, undernutrition, which was present in 26% of children and adolescents, greatly increased the odds of abnormal spirometry at the EOT; adjusted OR (aOR) of 8.30 (95% CI 2.0 to 35.2).^43^ The sole paper that used SGRQ found higher (worse) median scores in persons with undernutrition; 10 (IQR 4–22) versus 6 (IQR 2–17) in low BMI and normal BMI, respectively).^27^

One paper found reduced odds of abnormal spirometry if overweight (uOR 0.20 (0.06–0.77)).^25^ The other two papers did not find evidence of an association between abnormal spirometry and obesity^35^ or being overweight.^39^

Four papers which analysed BMI as a continuous variable used spirometry.^21 36 41 56^ Three reported worsening lung function with reducing BMI; aOR for airway obstruction 1.14, 95% CI 1.04 to 1.27 per each unit decrease in BMI,^21^ or increased absolute FEV1 and FVC with each unit increase in BMI.^36 41^ The other reported reducing FEV1 and FVC (% predicted) but increasing FEV1/FVC with increasing BMI.^56^ St. George’s Respiratory Questionnaire (SQRQ) score increased (worsened) by 0.57 with each unit increase in BMI,^56^ conflicting with that found in undernutrition.^27^ 6MWT distance decreased as BMI increased in one paper, though few participants were undernourished; BMI IQR 18.5–25.6.^21^ No evidence of an association was found with BMI and persistent symptoms (two papers)^36 42^ or pulmonary hypertension (one paper).^20^

### Alcohol use disorder

Seven papers assessed the association between alcohol use disorder (AUD) and PTLD (online supplemental table S11),^26 28 33 40 42 55 58^ three of which used spirometry assessment.^26 28 33^ No meta-analysis was possible as only two papers had extractable ORs. No overall evidence of an association between AUD and abnormal spirometry was seen, but there were only 168 participants with AUD in total and definitions of AUD varied across these studies (online supplemental table S11). According to symptomology, one paper reported increased odds of PTLD with ‘alcoholism’ although with a wide CI (aOR 3.1, 95% CI 1.16 to 8.30),^42^ and one point estimate suggested reduced odds but with a wide CI inclusive of both a null effect and increased odds (aOR 0.26, 95% CI 0.12 to 5.37).^40^ Neither paper defined how alcoholism was assessed. No evidence of an association between AUD and PTLD was found using CXR^55^ or the Chronic Obstructive Pulmonary Disease (COPD) Assessment Test.^58^

Four additional papers studied the association between any alcohol consumption and PTLD.^21 38 48 56^ There was no overall evidence of an association between alcohol consumption and PTLD using spirometry,^21 48 56^ 6MWT^21^ or composite assessment.^38^

### Other comorbidities

Papers analysing other comorbidities are described in online supplemental table S12. There was insufficient literature for any of these for evidence synthesis or meta-analysis. Associations between all comorbidities and PTLD risk are summarised in table 2, which also demonstrates the high risk of bias and limited volume of data for each association.

## Discussion

In this review, we primarily sought to explore whether multimorbidity, defined as two or more LTCs in addition to TB, is associated with an increased risk of PTLD in LMICs. We found no studies that addressed this question directly. Multimorbidity is increasing in high-burden TB countries,^61^ and patients with TB are more likely to be multimorbid.^62^ Patients with multimorbidity have worse WHO TB treatment outcomes,^63^ but the greatest burden is likely chronic morbidity following TB disease. Future research into TB-multimorbidity in LMICs needs to pay attention to its effect on long-term morbidity such as PTLD.

The definition of PTLD chosen at the first international PTLD symposium is patient-orientated and globally practical for capturing the broad variety of presentations of lung damage following TB disease.^7^ However, the range of recommended assessment tools (online supplemental table S2) capture very different aspects of respiratory health which are not well correlated and may not all be clinically relevant.^21^ This review demonstrates that the inconsistency in assessments used in PTLD research is substantially limiting evidence synthesis. Only three hypotheses had sufficient uniformity in definitions of comorbidities and PTLD to allow meta-analysis and GRADE assessment. We hope that the refined PTLD definition published in 2025—‘evidence of pulmonary disease in at least two of three domains (lung function, respiratory symptoms and imaging) that is attributable at least in part to previous tuberculosis disease’—will facilitate research into its determinants.^64^ Until that is extensively deployed, caution is needed when meta-analysing datasets which assess PTLD differently.

Most included papers (34/41, 82.9%) were at very high or high risk of bias in their assessments of comorbidities and PTLD, largely driven by residual confounding. An assessment of the effect of comorbidities on risk of PTLD, although extractable from 41 papers, was not their primary objective. Large, prospective cohort studies designed to determine the effect of either individual comorbidities or multimorbidity, with confounders identified a priori and adjusted for in analyses, are required to address this question with better certainty.

The reduced risk of PTLD in PLWHIV seen in this meta-analysis, although with very low certainty, is consistent with a previous meta-analysis which reported a lower prevalence of PTLD in PLWHIV than HIV-negative participants (32.8% vs 66.9%, respectively, p=0.0013).^5^ We have strengthened the assessment of this by using search terms that identified more studies, including only papers with an internal comparison by HIV status and grouping analysis by PTLD assessment method. Several plausible mechanisms may explain the observed direction of effect. HIV-TB coinfected patients often present with less extensive radiological disease and are more likely to have paucibacillary TB.^65 66^ PLWHIV are proactively screened for TB and may be diagnosed earlier, further reducing risk of PTLD. If the immune responses which drive cavity formation and parenchymal destruction in pulmonary TB are impaired by HIV coinfection, TB-associated lung damage may be less.^67^ However, any protective effects at TB diagnosis may be counteracted by immune restoration soon after anti-retroviral therapy initiation, which can drive pulmonary inflammation and reduce lung function.^36 68^ Future studies should stratify analysis by markers of immune suppression and reconstitution during TB treatment, rather than relying solely on binary HIV status.

We observed a direction of effect towards increased odds of PTLD in patients with diabetes; however, the estimate was imprecise and evidence very uncertain. As with HIV, degree of immunosuppression in diabetes depends on degree of disease control.^69 70^ An oversimplified analysis of risk of PTLD dependent on the presence or absence of diabetes may mask a more complex association. No evidence of an association between AUD and PTLD was seen. However, the small number of studies, variation in AUD definitions and lack of adjustment for confounders mean that a true association cannot be ruled out.

Undernutrition may be associated with increased odds of PTLD, but the evidence is very uncertain. Undernutrition increases the risk of acquiring TB disease, having more severe disease and having a poor TB treatment outcome.^14^,^16^ This association with PTLD is therefore unsurprising. However, nutritional state can fluctuate over short periods of time and PTLD syndromes such as COPD can drive a catabolic state. The predominance of cross-sectional studies, with BMI assessed at the same timepoint as PTLD, prevents determination of causality. Undernutrition also reflects a wide range of underlying factors—other comorbidities such as HIV, socio-economic circumstances, physical activity and, importantly for this review, TB disease severity. Similar considerations apply to haemoglobin, where anaemia may act both as a severity marker for HIV or TB and as an independent morbidity. Establishing causal relationships would require careful measurement and adjustment for confounders, which none of the meta-analysed studies did, and it will remain difficult to fully separate the effects of undernutrition or anaemia from those of TB severity. Nonetheless, BMI and haemoglobin at TB diagnosis may be useful for risk stratifying patients and to guide interventions.

There were no studies of prior respiratory disease which had an assessment of lung disease before development of TB. Without prospective studies, which assess respiratory disease severity prior to TB disease, it cannot be assumed that any observed associations between respiratory disease and PTLD seen are due to post-TB pathology, rather than the underlying respiratory disease itself.

Only one study looked at mental health.^24^ A high proportion of patients with TB have depression and this adversely affects treatment adherence, frequency of adverse events and EOT outcomes.^71 72^ A meta-review by Jarde *et al*, reported that coexistence of depression and TB, rather than HIV-TB, carried greater odds of adverse TB treatment outcomes.^73^ There is an increasing mandate for depression screening and care to be incorporated into TB management.^74 75^ Implementation of this, including differentiating depression from TB symptoms, creates an opportunity for exploration of the relationship between depression and PTLD using routine data.

The main strengths of this review are the rigorous methodological approach, use of patient and public involvement, inclusion of non-English language studies and broad search criteria, which included all the recommended PTLD assessment tools.^7^ These provide confidence that this is a broad, comprehensive summary of current evidence on the effect of comorbidities on PTLD risk. The main limitation of the review is that no multimorbidity studies were found. This highlights a critical research gap. Included studies had high risk of bias and were predominantly cross-sectional and small, particularly for some comorbidities. This limited our assessment of associations and contributed to low levels of certainty.

## Conclusion

There may be increased odds of PTLD in adults who are HIV-negative or undernourished, but the evidence is very uncertain and underlying mechanisms require further investigation. There is a critical need for high-quality, prospective studies delineating relationships between comorbidities and risk of PTLD. This is the first step towards identifying and implementing interventions to reduce the burden of PTLD, both for individuals and healthcare systems.

## Supplementary material

10.1136/bmjgh-2025-020365 online supplemental file 1

10.1136/bmjgh-2025-020365 online supplemental file 2

## Data Availability

All data relevant to the study are included in the article or uploaded as supplementary information.
