# Supplementary material for: Multimorbidity and the risk of post-tuberculosis lung disease: a systematic review and meta-analysis
Source: BMJ Glob Health. 2026 Jun 1;11(6):e020365. doi: 10.1136/bmjgh-2025-020365 (PMC13239530; doi:10.1136/bmjgh-2025-020365)
Supplement: online supplemental file 1 [file bmjgh-11-6-s001.docx]

**Appendices**

[**Supplementary Table S1: Database search terms** 5](#_Toc220664257)

[**Supplementary Table S2: Description of PTLD Measurement Tools** 12](#_Toc220664258)

[**Supplementary Table S3: Inclusion and exclusion criteria** 13](#_Toc220664259)

[**Supplementary Table S4: Grading of Recommendations Assessment, Development and Evaluation (GRADE) Framework Methods** 16](#_Toc220664260)

[**Supplementary Table S5: R code for included meta-analyses** 17](#_Toc220664261)

[**Supplementary Table S6: Extended Summary of Included Papers** 22](#_Toc220664262)

[**Supplementary Table S7: Risk of bias assessment** 24](#_Toc220664263)

[**Supplementary Figure S1: Geographical distribution of papers by WHO region** 26](#_Toc220664264)

[**Supplementary Table S8: Summary of included HIV papers** 27](#_Toc220664265)

[**Supplementary Table S9: Summary of included diabetes papers** 29](#_Toc220664266)

[**Supplementary Table S10: Summary of included nutritional status papers** 31](#_Toc220664267)

[**Supplementary Table S11: Summary of included Alcohol Papers** 33](#_Toc220664268)

[**Supplementary Table S12: Summary of other included papers** 35](#_Toc220664269)

[**Supplementary Figure S2: Sensitivity analysis for meta-analysis of abnormal spirometry by HIV status in adults** 37](#_Toc220664270)

[**Supplementary Figure S3: Publication bias for HIV meta-analysis** 38](#_Toc220664271)

[**Supplementary Figure S4: Publication bias for diabetes meta-analysis** 39](#_Toc220664272)

[**Supplementary Figure S5: Diabetes forest plots – contrasting airway obstruction and restrictive spirometry pattern from Gupte 2019^18^** 40](#_Toc220664273)

[**Supplementary Figure S6: Publication bias for undernutrition meta-analysis** 42](#_Toc220664274)

[**Supplementary Table S13: GRADE Assessment Results** 43](#_Toc220664275)

[**Supplementary Table S14: Papers excluded at full text stage with brief reasons** 44](#_Toc220664276)

[**References used in appendices** 48](#_Toc220664277)

## **Supplementary Table S1: Database search terms**

| **Embase <1974 to 2024 March 07>** | | |
| --- | --- | --- |
|  |  |  |
| **#** | **Search query** | **Results** |
| 1 | exp Comorbidity/ | 409283 |
| 2 | exp Chronic Disease/ | 248803 |
| 3 | (multimorbid* or multi-morbid* or "multi morbid*" or (multiple chronic adj3 (disease* or condition* or illness* or morbid*)) or (concurrent chronic adj3 (disease* or condition* or illness* or morbid*)) or "multiple comorbid*" or comorbid* or co-morbid* or polymorbid* or poly-morbid* polypatholog* or poly-patholog*).mp. [mp=title, abstract, heading word, drug trade name, original title, device manufacturer, drug manufacturer, device trade name, keyword heading word, floating subheading word, candidate term word] | 657077 |
| 4 | exp non communicable disease/ | 12452 |
| 5 | (non communicable disease* or non-communicable disease* or noncommunicable disease* or noninfectious disease* or non infectious disease* or non-infectious disease*).mp. [mp=title, abstract, heading word, drug trade name, original title, device manufacturer, drug manufacturer, device trade name, keyword heading word, floating subheading word, candidate term word] | 27112 |
| 6 | (cardiovascular disease* or heart disease* or heart failure* or coronary infarction* or cardiac infarction* or heart infarction* or myocardial infarction* or angina* or arrythmia* or atrial fibrillation or hypertens* or high blood pressure* or hypercholesterol* or dyslipid$em* or peripheral arterial disease or venous thrombotic disease or chronic lung disease* or chronic respiratory disease* or chronic obstructive disease* or chronic obstructive airway disease* or chronic obstructive lung disease or asthma* or emphysema* or chronic obstructive pulmonary disease* or copd* or cystic fibrosis or bronchitis or pulmonary fibrosis or lung fibrosis or sarcoid* or bronchiectasis or silicosis or occupational lung disease or occupational pulmonary disease or (oncological adj2 (disease* or condition* or illness*)) or neoplasm* or cancer* or tumor* or tumour* or malignan* or leuk$em* or myeloma or anaemi* or anemi* or myelofibro* or diabetes* or diabetic* or addison's disease or thyroid disease* or stroke or cerebrovascular or transient isch$emic attack or epilepsy or (neurological adj2 (disease* or illness* or morbid* or condition*)) or parkinson's disease or polygenic disease* or polygenic disorder* or (mental adj2 (disorder* or health* or condition* or illness* or morbid*)) or depression* or (depressive adj2 disorder*) or personality disorder* or bipolar disease or schizo* or post-traumatic stress disorder* or PTSD* or anxiety* or anxious or dement* or inflammatory bowel disease* or chronic liver disease or cirrhosis or cirrhotic or alcohol* or smoking or smoker* or tobacco* or substance abuse or intravenous drug use* or drug abuse* or drug addict* or "body mass index" or "low BMI" or "high BMI" or malnutrition or malnourish* or obesity or obese or (autoimmune adj2 (disease* or illness* or condition* or morbid*)) or chronic pain or connective tissue disease or arthritis or paraylsed or paralysis or blind or visual impair* or deaf or ((renal or kidney) adj2 (disease* or illness* or condition* or morbid*)) or dialysis or chronic pancreatit* or arthritis or lupus).mp. [mp=title, abstract, heading word, drug trade name, original title, device manufacturer, drug manufacturer, device trade name, keyword heading word, floating subheading word, candidate term word] | 15672519 |
| 7 | (co-infect* or co infect* or coinfect* or HIV or PLWHIV or PLHIV or immunodeficiency virus* or immunodeficiency syndrome* or mixed infection* or malaria* or plasmodium infection* or ectoparasit* or hepatitis or liver disease* or liver infect* or ulcer* or pyloric infect* or pyloric disease* or schistosomiasis or syphilli*).mp. [mp=title, abstract, heading word, drug trade name, original title, device manufacturer, drug manufacturer, device trade name, keyword heading word, floating subheading word, candidate term word] | 1824316 |
| 8 | exp Coinfection/ | 25271 |
| 9 | exp blood-borne infection/ | 926332 |
| 10 | exp sexually transmitted disease/ | 112917 |
| 11 | 1 or 2 or 3 or 4 or 5 or 6 or 7 or 8 or 9 or 10 | 17113191 |
| 12 | lung mycosis/ or interstitial lung disease/ or lung complication/ or obstructive lung disease/ or pulmonary vascular disease/ | 51182 |
| 13 | exp chronic obstructive lung disease/ or exp interstitial lung disease/ | 314250 |
| 14 | (asthma* or aspergill* or bronchiecta* or bronchiolitis or (cavitation adj3 (lung or pulmonary or respiratory)) or chronic bronchitis or (chronic adj3 (lung or airway or pulmonary or respiratory)) or COPD or COAD or cor pulmonale or emphysema* or interstitial lung or lung disease or (obstructive adj3 (lung or airway or pulmonary or respiratory)) or pulmonary disease or pulmonary hypertension or pulmonary fibrosis or lung fibrosis or (restrictive adj3 (lung or airway or pulmonary or respiratory))).mp. [mp=title, abstract, heading word, drug trade name, original title, device manufacturer, drug manufacturer, device trade name, keyword heading word, floating subheading word, candidate term word] | 1021062 |
| 15 | (comput* tomography or chest x-ray or chest radiograph or spirometry or pulmonary function or respiratory function or lung function or six-minute walking test or six minute walking test or St George's respiratory questionnaire or forced vital capacity or forced expiratory volume).mp. [mp=title, abstract, heading word, drug trade name, original title, device manufacturer, drug manufacturer, device trade name, keyword heading word, floating subheading word, candidate term word] | 1058541 |
| 16 | exp x-ray computed tomography/ | 110273 |
| 17 | exp lung function test/ | 227287 |
| 18 | 12 or 13 or 14 or 15 or 16 or 17 | 2034504 |
| 19 | ((post* adj5 (TB or tuberc* or Mtb or PTB or EPTB)) or (sequela* adj5 (TB or tuberc* or Mtb or PTB or EPTB)) or (following adj5 (TB or tuberc* or Mtb or PTB or EPTB)) or ("residual damage*" adj5 (TB or tuberc* or Mtb or PTB or EPTB)) or (after* adj5 (TB or tuberc* or Mtb or PTB or EPTB)) or ("destroyed lung" adj5 (TB or tuberc* or Mtb or PTB or EPTB))).mp. | 22370 |
| 20 | 18 and 19 | 4611 |
| 21 | 20 and 11 | 4338 |

| **Ovid MEDLINE(R) ALL <1946 to March 07, 2024>** | |  |
| --- | --- | --- |
|  |  |  |
| **#** | **Search query** | **Results** |
| 1 | exp Comorbidity/ | 129177 |
| 2 | exp Chronic Disease/ | 639655 |
| 3 | (multimorbid* or multi-morbid* or "multi morbid*" or (multiple chronic adj3 (disease* or condition* or illness* or morbid*)) or (concurrent chronic adj3 (disease* or condition* or illness* or morbid*)) or "multiple comorbid*" or comorbid* or co-morbid* or polymorbid* or poly-morbid* polypatholog* or poly-patholog*).mp. [mp=title, book title, abstract, original title, name of substance word, subject heading word, floating sub-heading word, keyword heading word, organism supplementary concept word, protocol supplementary concept word, rare disease supplementary concept word, unique identifier, synonyms, population supplementary concept word, anatomy supplementary concept word] | 356210 |
| 4 | exp Noncommunicable Diseases/ | 3437 |
| 5 | (non communicable disease* or non-communicable disease* or noncommunicable disease* or noninfectious disease* or non infectious disease* or non-infectious disease*).mp. [mp=title, book title, abstract, original title, name of substance word, subject heading word, floating sub-heading word, keyword heading word, organism supplementary concept word, protocol supplementary concept word, rare disease supplementary concept word, unique identifier, synonyms, population supplementary concept word, anatomy supplementary concept word] | 19938 |
| 6 | (cardiovascular disease* or heart disease* or heart failure* or coronary infarction* or cardiac infarction* or heart infarction* or myocardial infarction* or angina* or arrythmia* or atrial fibrillation or hypertens* or high blood pressure* or hypercholesterol* or dyslipid$em* or peripheral arterial disease or venous thrombotic disease or chronic lung disease* or chronic respiratory disease* or chronic obstructive disease* or chronic obstructive airway disease* or chronic obstructive lung disease or asthma* or emphysema* or chronic obstructive pulmonary disease* or copd* or cystic fibrosis or bronchitis or pulmonary fibrosis or lung fibrosis or sarcoid* or bronchiectasis or silicosis or occupational lung disease or occupational pulmonary disease or (oncological adj2 (disease* or condition* or illness*)) or neoplasm* or cancer* or tumor* or tumour* or malignan* or leuk$em* or myeloma or anaemi* or anemi* or myelofibro* or diabetes* or diabetic* or addison's disease or thyroid disease* or stroke or cerebrovascular or transient isch$emic attack or epilepsy or (neurological adj2 (disease* or illness* or morbid* or condition*)) or parkinson's disease or polygenic disease* or polygenic disorder* or (mental adj2 (disorder* or health* or condition* or illness* or morbid*)) or depression* or (depressive adj2 disorder*) or personality disorder* or bipolar disease or schizo* or post-traumatic stress disorder* or PTSD* or anxiety* or anxious or dement* or inflammatory bowel disease* or chronic liver disease or cirrhosis or cirrhotic or alcohol* or smoking or smoker* or tobacco* or substance abuse or intravenous drug use* or drug abuse* or drug addict* or "body mass index" or "low BMI" or "high BMI" or malnutrition or malnourish* or obesity or obese or (autoimmune adj2 (disease* or illness* or condition* or morbid*)) or chronic pain or connective tissue disease or arthritis or paraylsed or paralysis or blind or visual impair* or deaf or ((renal or kidney) adj2 (disease* or illness* or condition* or morbid*)) or dialysis or chronic pancreatit* or arthritis or lupus).mp. [mp=title, book title, abstract, original title, name of substance word, subject heading word, floating sub-heading word, keyword heading word, organism supplementary concept word, protocol supplementary concept word, rare disease supplementary concept word, unique identifier, synonyms, population supplementary concept word, anatomy supplementary concept word] | 11466663 |
| 7 | (co-infect* or co infect* or coinfect* or HIV or PLWHIV or PLHIV or immunodeficiency virus* or immunodeficiency syndrome* or mixed infection* or malaria* or plasmodium infection* or ectoparasit* or hepatitis or liver disease* or liver infect* or ulcer* or pyloric infect* or pyloric disease* or schistosomiasis or syphilli*).mp. [mp=title, book title, abstract, original title, name of substance word, subject heading word, floating sub-heading word, keyword heading word, organism supplementary concept word, protocol supplementary concept word, rare disease supplementary concept word, unique identifier, synonyms, population supplementary concept word, anatomy supplementary concept word] | 1328879 |
| 8 | exp Coinfection/ | 15880 |
| 9 | exp blood-borne infections/ or exp hiv infections/ | 433263 |
| 10 | exp sexually transmitted diseases/ | 415073 |
| 11 | 1 or 2 or 3 or 4 or 5 or 6 or 7 or 8 or 9 or 10 | 12654431 |
| 12 | exp Lung diseases, obstructive/ or exp Lung Diseases, Interstitial/ or exp Pulmonary Arterial Hypertension/ or exp Pulmonary Aspergillosis/ | 327854 |
| 13 | (asthma* or aspergill* or bronchiecta* or bronchiolitis or (cavitation adj3 (lung or pulmonary or respiratory)) or chronic bronchitis or (chronic adj3 (lung or airway or pulmonary or respiratory)) or COPD or COAD or cor pulmonale or emphysema* or interstitial lung or lung disease or (obstructive adj3 (lung or airway or pulmonary or respiratory)) or pulmonary disease or pulmonary hypertension or pulmonary fibrosis or lung fibrosis or (restrictive adj3 (lung or airway or pulmonary or respiratory))).mp. [mp=title, book title, abstract, original title, name of substance word, subject heading word, floating sub-heading word, keyword heading word, organism supplementary concept word, protocol supplementary concept word, rare disease supplementary concept word, unique identifier, synonyms, population supplementary concept word, anatomy supplementary concept word] | 564211 |
| 14 | (comput* tomography or chest x-ray or chest radiograph or spirometry or pulmonary function or respiratory function or lung function or six-minute walking test or six minute walking test or St George's respiratory questionnaire or forced vital capacity or forced expiratory volume).mp. [mp=title, book title, abstract, original title, name of substance word, subject heading word, floating sub-heading word, keyword heading word, organism supplementary concept word, protocol supplementary concept word, rare disease supplementary concept word, unique identifier, synonyms, population supplementary concept word, anatomy supplementary concept word] | 577191 |
| 15 | exp Tomography, X-Ray Computed/ | 500165 |
| 16 | exp Respiratory Function Tests/ | 251971 |
| 17 | 12 or 13 or 14 or 15 or 16 | 1496540 |
| 18 | ((post* adj5 (TB or tuberc* or Mtb or PTB or EPTB)) or (sequela* adj5 (TB or tuberc* or Mtb or PTB or EPTB)) or (following adj5 (TB or tuberc* or Mtb or PTB or EPTB)) or ("residual damage*" adj5 (TB or tuberc* or Mtb or PTB or EPTB)) or (after* adj5 (TB or tuberc* or Mtb or PTB or EPTB)) or ("destroyed lung" adj5 (TB or tuberc* or Mtb or PTB or EPTB))).mp. | 17742 |
| 19 | 17 and 18 | 2860 |
| 20 | 19 and 11 | 1667 |

| **Web of Science** | | |
| --- | --- | --- |
|  |  |  |
| **#** | **Search Query** | **Results** |
| 1 | TS=(multimorbid* or multi-morbid* or “multi morbid*” or ("multiple chronic" near/3 (disease* or condition* or illness* or morbid*)) or ("concurrent chronic" near/3 (disease* or condition* or illness* or morbid*)) or "multiple comorbid*" or comorbid* or "co-morbid*" or polymorbid* or "poly-morbid*" or polypatholog* or "poly-patholog*" or "non-communicable disease*" OR "noncommunicable disease*" OR "noninfectious disease*" OR "non infectious disease*" OR "non-infectious disease*" "cardiovascular disease*" OR "heart disease*" OR "heart failure*" OR "coronary infarction*" OR "cardiac infarction*" OR "heart infarction*" OR "myocardial infarction*" OR angina* OR arrythmia* OR "atrial fibrillation" OR hypertens* OR "high blood pressure*" OR hypercholesterol* OR dyslipidaem* OR dyslipidemia OR "peripheral arterial disease" OR "venous thrombotic disease" OR "chronic lung disease*" OR "chronic respiratory disease*" OR "chronic obstructive disease*" OR "chronic obstructive airway disease*" OR "chronic obstructive lung disease" OR asthma* OR emphysema* OR "chronic obstructive pulmonary disease*" OR copd* OR "cystic fibrosis" OR bronchitis OR "pulmonary fibrosis" OR "lung fibrosis" OR sarcoid* OR bronchiectasis OR silicosis OR "occupational lung disease" OR "occupational pulmonary disease" OR (oncological near/2 (disease* OR condition* OR illness*)) OR neoplasm* OR cancer* OR tumor* OR tumour* OR malignan* OR leukaem* OR leukemia OR myeloma OR anaemi* OR anemi* OR myelofibro* OR diabetes* OR diabetic* OR "addison's disease" OR "thyroid disease*" OR stroke OR cerebrovascular OR "transient ischaemic attack" OR "transient ischemic attack" OR epilepsy OR (neurological near/2 (disease* OR illness* or morbid* OR condition*)) OR "parkinson's disease" OR "polygenic disease*" OR "polygenic disorder*" OR (mental near/2 (disorder* or health* or condition* or illness* or morbid*)) OR depression* OR (depressive near/2 disorder*) OR "personality disorder*" OR "bipolar disease" OR schizo* OR "post-traumatic stress disorder*" OR PTSD OR anxiety OR anxious OR dement* OR "inflammatory bowel disease*" OR "chronic liver disease" OR cirrhosis OR cirrhotic OR alcohol* OR smoking OR smoker* OR tobacco* OR substance abuse OR intravenous drug use* OR drug abuse* OR drug addict* OR “body mass index” OR “low BMI” OR “high BMI” OR malnutrition OR malnourish* OR obesity OR obese OR (autoimmune near/2 (disease* OR illness* OR condition* OR morbid*)) OR "chronic pain" OR "connective tissue disease" OR paralysed OR paralysis OR blind OR "visual impair*" OR deaf OR ((renal OR kidney) near/2 (disease* OR illness* OR condition* OR morbid*)) OR dialysis OR "chronic pancreatit*" OR arthritis OR lupus OR coinfection OR "HIV infection" OR "blood borne infection" OR "sexually transmitted disease*" or co-infect* or "co infect*" or coinfect* or PLWHIV or PLHIV or HIV* OR "immunodeficiency virus*" OR "immunodeficiency syndrome*" OR "mixed infection*" OR malaria* OR "plasmodium infection*" OR ectoparasit* OR hepatitis OR "liver disease*" OR "liver infect*" OR ulcer* OR "pyloric infect*" OR "pyloric disease*" OR schistosomiasis OR syphilli*) and Preprint Citation Index (Exclude – Database) | 27946441 |
| 2 | TS=((post* near/5 (TB or tuberc* or Mtb or PTB or EPTB)) or (sequela* near/5 (TB or tuberc* or Mtb or PTB or EPTB)) or (following near/5 (TB or tuberc* or Mtb or PTB or EPTB)) or ("residual damage*" near/5 (TB or tuberc* or Mtb or PTB or EPTB)) or (after* near/5 (TB or tuberc* or Mtb or PTB or EPTB)) or ("destroyed lung" near/5 (TB or tuberc* or Mtb or PTB or EPTB))) and Preprint Citation Index (Exclude – Database) | 37997 |
| 3 | TS=(asthma* or aspergill* or bronchiecta* or bronchiolitis or (cavitation near/3 (lung or pulmonary or respiratory)) or "chronic bronchitis" or (chronic near/3 (lung or airway or pulmonary or respiratory)) or COPD or COAD or emphysema* or "interstitial lung" or (obstructive near/3 (lung or airway or pulmonary or respiratory)) or "lung disease" or "pulmonary disease" or "pulmonary hypertension" or "pulmonary fibrosis" or "lung fibrosis" or (restrictive near/3 (lung or airway or pulmonary or respiratory)) or "cor pulmonale" OR "comput* tomography" OR "chest x-ray" OR "chest radiograph*" OR "forced expiratory volume" OR "forced vital capacity" OR spirometry OR "pulmonary function" Or "respiratory function" OR "lung function" OR "six-minute walking test" OR "six minute walking test" OR "St George’s respiratory questionnaire") and Preprint Citation Index (Exclude – Database) | 2266887 |
| 4 | #2 AND #3 and Preprint Citation Index (Exclude – Database) | 5111 |
| 5 | #4 AND #1 and Preprint Citation Index (Exclude – Database) | 3250 |

| **Scopus** |  |
| --- | --- |
|  |  |
| **Search Query** | **Results** |
| ((TITLE-ABS-KEY((post* W/5 (TB or tuberc* or Mtb or PTB or EPTB)) or (sequela* W/5 (TB or tuberc* or Mtb or PTB or EPTB)) or (following W/5 (TB or tuberc* or Mtb or PTB or EPTB)) or ("residual damage*" W/5 (TB or tuberc* or Mtb or PTB or EPTB)) or (after* W/5 (TB or tuberc* or Mtb or PTB or EPTB)) or ("destroyed lung" W/5 (TB or tuberc* or Mtb or PTB or EPTB)))) AND (TITLE-ABS-KEY(asthma* or aspergill* or bronchiecta* or bronchiolitis or ( cavitation w/3 ( lung or pulmonary or respiratory ) ) or "chronic bronchitis" or ( chronic pre/3 ( lung or airway or pulmonary or respiratory ) ) or copd or coad or emphysema* or "interstitial lung disease" or (obstructive pre/3 ( lung or airway or pulmonary or respiratory ) ) or "lung disease" OR "pulmonary disease" or "pulmonary hypertension" or "pulmonary fibrosis" or "lung fibrosis" or (restrictive pre/3 ( lung or airway or pulmonary or respiratory ) ) or "cor pulmonale" or "comput* tomography" or "chest x-ray" or "chest radiograph*" or "forced expiratory volume" or "forced vital capacity" or spirometry or "pulmonary function" or "respiratory function" or "lung function" or "six-minute walking test" or "six minute walking test" or "st george* respiratory questionnaire"))) AND (TITLE-ABS-KEY(multimorbid* OR multi-morbid* OR "multi morbid*" OR ( "multiple chronic" PRE/3 ( disease* OR condition* OR illness* OR morbid* ) ) OR ( "concurrent chronic" PRE/3 ( disease* OR condition* OR illness* OR morbid* ) ) OR "multiple comorbid*" OR comorbid* OR "co-morbid*" OR polymorbid* OR "poly-morbid*" OR polypatholog* OR "poly-patholog*" OR "non-communicable disease*" OR "noncommunicable disease*" OR "noninfectious disease*" OR "non infectious disease*" OR "non-infectious disease*" "cardiovascular disease*" OR "heart disease*" OR "heart failure*" OR "coronary infarction*" OR "cardiac infarction*" OR "heart infarction*" OR "myocardial infarction*" OR angina* OR arrythmia* OR "atrial fibrillation" OR hypertens* OR "high blood pressure*" OR hypercholesterol* OR dyslipidaem* OR dyslipidem* OR "peripheral arterial disease" OR "venous thrombotic disease" OR "chronic lung disease*" OR "chronic respiratory disease*" OR "chronic obstructive disease*" OR "chronic obstructive airway disease*" OR "chronic obstructive lung disease" OR asthma* OR emphysema* OR "chronic obstructive pulmonary disease*" OR copd* OR "cystic fibrosis" OR bronchitis OR "pulmonary fibrosis" OR "lung fibrosis" OR sarcoid* OR bronchiectasis OR silicosis OR "occupational lung disease" OR "occupational pulmonary disease" OR ( oncological PRE/2 ( disease* OR condition* OR illness* ) ) OR neoplasm* OR cancer* OR tumor* OR tumour* OR malignan* OR leukaem* OR leukemia OR myeloma OR anaemi* OR anemi* OR myelofibro* OR diabetes* OR diabetic* OR "addison's disease" OR "thyroid disease*" OR stroke OR cerebrovascular OR "transient ischaemic attack" OR "transient ischemic attack" OR epilepsy OR ( neurological PRE/2 ( disease* OR illness* OR morbid* OR condition* ) ) OR "polygenic disease*" OR "polygenic disorder*" OR "parkinson's disease" OR ( mental PRE/2 ( disorder* OR health* OR condition* OR illness* OR morbid* ) ) OR depression* OR ( depressive PRE/2 disorder* ) OR "personality disorder*" OR schizo* OR "post-traumatic stress disorder*" OR anxiety OR anxious OR dement* OR "inflammatory bowel disease*" OR "chronic liver disease" OR cirrhosis OR cirrhotic OR alcohol* OR smoking OR smoker* OR tobacco* OR "substance abuse" OR "intravenous drug use*" OR "drug abuse*" OR "drug addict*" OR "body mass index" OR "low bmi" OR "high bmi" OR malnutrition OR malnourish* OR ( autoimmune PRE/2 ( disease* OR illness* OR condition* OR morbid* ) ) OR "chronic pain" OR "connective tissue disease" OR paralysed OR paralysis OR blind OR "visual impair*" OR deaf OR ( ( renal OR kidney ) PRE/2 ( disease* OR illness* OR condition* OR morbid* ) ) OR dialysis OR "chronic pancreatit*" OR arthritis OR lupus OR obesity OR obese OR coinfection OR "hiv infection" OR "blood borne infection" OR "sexually transmitted disease*" OR co-infect* OR "co infect*" OR coinfect* OR plwhiv OR plhiv OR hiv* OR "immunodeficiency virus*" OR "immunodeficiency syndrome*" OR "mixed infection*" OR malaria* OR "plasmodium infection*" OR ectoparasit* OR hepatitis OR "liver disease*" OR "liver infect*" OR ulcer* OR "pyloric infect*" OR "pyloric disease*" OR schistosomiasis OR syphilli*)) | 194 |

| **Global health** | | |
| --- | --- | --- |
|  |  |  |
| **#** | **Search Query** | **Results** |
| 1 | TS=(multimorbid* or multi-morbid* or “multi morbid*” or ("multiple chronic" near/3 (disease* or condition* or illness* or morbid*)) or ("concurrent chronic" near/3 (disease* or condition* or illness* or morbid*)) or "multiple comorbid*" or comorbid* or "co-morbid*" or polymorbid* or "poly-morbid*" or polypatholog* or "poly-patholog*" or "non-communicable disease*" OR "noncommunicable disease*" OR "noninfectious disease*" OR "non infectious disease*" OR "non-infectious disease*" "cardiovascular disease*" OR "heart disease*" OR "heart failure*" OR "coronary infarction*" OR "cardiac infarction*" OR "heart infarction*" OR "myocardial infarction*" OR angina* OR arrythmia* OR "atrial fibrillation" OR hypertens* OR "high blood pressure*" OR hypercholesterol* OR dyslipidaem* OR dyslipidemia OR "peripheral arterial disease" OR "venous thrombotic disease" OR "chronic lung disease*" OR "chronic respiratory disease*" OR "chronic obstructive disease*" OR "chronic obstructive airway disease*" OR "chronic obstructive lung disease" OR asthma* OR emphysema* OR "chronic obstructive pulmonary disease*" OR copd* OR "cystic fibrosis" OR bronchitis OR "pulmonary fibrosis" OR "lung fibrosis" OR sarcoid* OR bronchiectasis OR silicosis OR "occupational lung disease" OR "occupational pulmonary disease" OR (oncological near/2 (disease* OR condition* OR illness*)) OR neoplasm* OR cancer* OR tumor* OR tumour* OR malignan* OR leukaem* OR leukemia OR myeloma OR anaemi* OR anemi* OR myelofibro* OR diabetes* OR diabetic* OR "addison's disease" OR "thyroid disease*" OR stroke OR cerebrovascular OR "transient ischaemic attack" OR "transient ischemic attack" OR epilepsy OR (neurological near/2 (disease* OR illness* or morbid* OR condition*)) OR "parkinson's disease" OR "polygenic disease*" OR "polygenic disorder*" OR (mental near/2 (disorder* or health* or condition* or illness* or morbid*)) OR depression* OR (depressive near/2 disorder*) OR "personality disorder*" OR "bipolar disease" OR schizo* OR "post-traumatic stress disorder*" OR PTSD OR anxiety OR anxious OR dement* OR "inflammatory bowel disease*" OR "chronic liver disease" OR cirrhosis OR cirrhotic OR alcohol* OR smoking OR smoker* OR tobacco* OR substance abuse OR intravenous drug use* OR drug abuse* OR drug addict* OR “body mass index” OR “low BMI” OR “high BMI” OR malnutrition OR malnourish* OR obesity OR obese OR (autoimmune near/2 (disease* OR illness* OR condition* OR morbid*)) OR "chronic pain" OR "connective tissue disease" OR paralysed OR paralysis OR blind OR "visual impair*" OR deaf OR ((renal OR kidney) near/2 (disease* OR illness* OR condition* OR morbid*)) OR dialysis OR "chronic pancreatit*" OR arthritis OR lupus OR coinfection OR "HIV infection" OR "blood borne infection" OR "sexually transmitted disease*" or co-infect* or "co infect*" or coinfect* or PLWHIV or PLHIV or HIV* OR "immunodeficiency virus*" OR "immunodeficiency syndrome*" OR "mixed infection*" OR malaria* OR "plasmodium infection*" OR ectoparasit* OR hepatitis OR "liver disease*" OR "liver infect*" OR ulcer* OR "pyloric infect*" OR "pyloric disease*" OR schistosomiasis OR syphilli*) and Preprint Citation Index (Exclude – Database) | 2,767,195 |
| 2 | TS=((post* near/5 (TB or tuberc* or Mtb or PTB or EPTB)) or (sequela* near/5 (TB or tuberc* or Mtb or PTB or EPTB)) or (following near/5 (TB or tuberc* or Mtb or PTB or EPTB)) or ("residual damage*" near/5 (TB or tuberc* or Mtb or PTB or EPTB)) or (after* near/5 (TB or tuberc* or Mtb or PTB or EPTB)) or ("destroyed lung" near/5 (TB or tuberc* or Mtb or PTB or EPTB))) and Preprint Citation Index (Exclude – Database) | 13,466 |
| 3 | TS=(asthma* or aspergill* or bronchiecta* or bronchiolitis or (cavitation near/3 (lung or pulmonary or respiratory)) or "chronic bronchitis" or (chronic near/3 (lung or airway or pulmonary or respiratory)) or COPD or COAD or emphysema* or "interstitial lung" or (obstructive near/3 (lung or airway or pulmonary or respiratory)) or "lung disease" or "pulmonary disease" or "pulmonary hypertension" or "pulmonary fibrosis" or "lung fibrosis" or (restrictive near/3 (lung or airway or pulmonary or respiratory)) or "cor pulmonale" OR "comput* tomography" OR "chest x-ray" OR "chest radiograph*" OR "forced expiratory volume" OR "forced vital capacity" OR spirometry OR "pulmonary function" Or "respiratory function" OR "lung function" OR "six-minute walking test" OR "six minute walking test" OR "St George’s respiratory questionnaire") and Preprint Citation Index (Exclude – Database) | 211,270 |
| 4 | #1 AND #2 AND #3 and Preprint Citation Index (Exclude – Database) | 869 |

## **Supplementary Table S2: Description of PTLD Measurement Tools**

As directed by Allwood et al^1^, the included PTLD measurement tools were as follows:

| **Parameter** | **PTLD Measurement Tool** | **Description** |
| --- | --- | --- |
| Self-reported symptoms | Shortness of breath (dyspnoea), cough, sputum, wheeze, chest pain, haemoptysis, fatigue | Shortness of breath is frequently quantified using a scale such a modified medical research council (mMRC) dyspnoea scale. This scale rates dyspnoea from 0 (breathless only on strenuous exercise) to 4 (too breathless to leave the house, or breathless when dressing or undressing). |
| Lung function | Spirometry | Spirometry measures the volume of air exhaled at specific time points during forced exhalation. Variables reported include forced expiratory volume in 1 second (FEV1), forced vital capacity (FVC) and their ratio (FEV1/FVC). |
| Radiology | Chest X-ray | 2D X-ray image |
|  | Computer tomography (CT) | Takes a series of cross-sectional X-ray images, giving more detail and allowing 3D reconstruction. |
| Functional capacity | Six-minute walk test (6MWT) | The 6MWT is a simple and easy to perform measure of lung functional capacity. Patients are asked to walk as far as possible in 6 minutes around a track of defined distance. The primary outcome is total distance covered at the end of 6 minutes is recorded and this has been predictive of morbidity and mortality in COPD, pulmonary arterial hypertension and idiopathic pulmonary fibrosis. |
|  | Incremental shuttle walk test (ISWT) | The ISWT also assesses lung functional capacity. Patients are asked to walk around a 10m track in time to a set of auditory beeps. Initially the walking speed is very slow but each minute the walking speed progressively increases. The patients continue to walk until they are either too breathless or can no longer keep up with the beeps. The primary outcome is the number of shuttles achieved. |
| Health-related quality of life | St. George’s Respiratory Questionnaire (SGRQ) | SGRQ is a 50-item questionnaire that assesses symptoms, activity and psychosocial impact in chronic lung disease and has been used in the assessment of post-TB lung disease. Scores range from 0 to 100, with higher scores indicating worse health. |
|  | Chronic Obstructive Pulmonary Disorder (COPD) Assessment Test (CAT) | CAT is a short, simple questionnaire for assessing and monitoring the impact of COPD on health. Increasing CAT scores have been shown to correlate with higher mortality in COPD. |
| Disease behaviour | Echocardiography | A type of ultrasound scan which looks at the structure and function of the heart and can detect evidence of pulmonary hypertension. |
|  | Aspergillus IgG | An antibody test that looks for evidence of aspergillus-related lung disease. |

## **Supplementary Table S3: Inclusion and exclusion criteria**

|  | **Inclusion** | **Exclusion** |
| --- | --- | --- |
| **Population** | Patients of any age with a clinical, radiological or microbiological diagnosis of TB disease involving the respiratory system – pulmonary, pleural, airway or miliary TB. | Patients with TB disease not involving the respiratory system. Patients with latent TB. |
| **Exposure** | Any morbidity in addition to TB. Inclusive of:  - Heart failure  - Ischaemic heart disease  - Cardiac arrythmia  - Hypertension  - Dyslipidaemia  - Peripheral vascular disease  - Venous thromboembolism  - Chronic obstructive pulmonary disease  - Asthma  - Cystic fibrosis  - Interstitial lung disease  - Bronchiectasis  - Malignancy  - Diabetes mellitus  - Autoimmune conditions  - Cerebrovascular disease  - Epilepsy  - Chronic neurological disease  - Mental health disorders  - Inflammatory bowel disease  - Chronic liver disease  - Alcohol use disorder (or measurement of alcohol consumption)  - Substance dependency  - Malnutrition (or measurement of nutritional status)  - Chronic pain  - Connective tissue disease  - Physical disability  - Visual impairment  - Hearing impairment  - Chronic kidney disease  - Anaemia (or measurement of serum haemoglobin)  - Hepatitis  - HIV  - Schistosomiasis  - Malaira  - Peptic ulcer disease  Morbidity can be measured before, during or after TB treatment. | Studies that have assessed TB and lung disease at or beyond end of treatment, but no other morbidities. |
| **Comparator** | Studies with participants assessed and found to not have the morbidity or morbidities assessed, in addition to TB or PTLD. |  |
| **Outcome** | International consensus definition of PTLD: evidence of chronic respiratory abnormality, with or without symptoms, attributable at least in part to previous tuberculosis  Assessed through respiratory symptoms, breathlessness scales, pulmonary function, lung imaging, gas exchange, functional capacity (e.g. 6MWT), cor pulmonale on echo | No assessment of respiratory system |
| **Types of studies** | Case-control, cohort and cross-sectional studies. Randomised controlled trials describing differences in morbidities within intervention arms will also be included, but only data from the control arm included in the analysis.  Abstracts and unpublished studies if sufficient detail and clarity to the data presented. | Case reports, case series, reviews, qualitative studies, and editorials.  Abstracts and pre-print papers if insufficient data reported and study not yet published in greater detail elsewhere. |
| **Country of study** | Studies involving participants from one or more LMIC as per World Bank | Participants only from HIC as per World Bank |
| **Other** | Human studies | Animal studies |

6MWT = six-minute walk test, HIC = high-income country, LMIC = low- and middle- income country, PTLD = post-tuberculosis lung disease, TB = tuberculosis.

## **Supplementary Table S4: Grading of Recommendations Assessment, Development and Evaluation (GRADE) Framework Methods**

The GRADE framework was used to assess the certainty of evidence for each meta-analysed association. The guidance laid out in the Journal of Clinical Epidemiology series was followed.^2^

Risk of bias was assessed using the Risk of Bias in Non-randomised Studies (ROBINS) tool, therefore randomised and non-randomised studies were initially rated as high certainty.^3^

The five domains which can downgrade the certainty of evidence in the GRADE framework are risk of bias, inconsistency, indirectness, imprecision and publication bias. The definition of these domains, described in further detail in referenced articles, were implemented in this review as per table S4a.^4-8^ The three domains which can upgrade the certainty of evidence are large effect, dose-response gradient and residual confounding which draws the effect estimate towards the null. These are described in further detail in referenced articles and implemented in this review as per table S4b.^9^

Table S4a: Definition of GRADE domains which may downgrade level of certainty

|  | **No serious limitation, do not downgrade** | **Serious limitation, downgrade one level** | **Very serious limitation, downgrade two levels** |
| --- | --- | --- | --- |
| **Risk of bias** | All studies are from low risk of bias or some concerns. Potential limitations are unlikely to lower confidence in the estimate of effect. | High risk or very high risk of bias in one study or some concerns in multiple studies, sufficient to lower confidence in the effect estimate. | Majority of studies were at high or very high risk of bias OR a single study which accounts for the majority of participants was at high or very high risk of bias. |
| **Inconsistency** | Neither I^2^ nor its 95% CI ≥50% | Either I^2^ ≥50%  OR upper limit of 95% CI ≥50% | Either I^2^ ≥50%  AND upper limit of 95% CI ≥50% |
| **Indirectness** | No indirectness in study population, co-morbidity definition, outcome assessment or comparison. | Indirectness in ONE of study population, co-morbidity definition, outcome assessment or comparison. | Indirectness in TWO OR MORE of study population, co-morbidity definition, outcome assessment or comparison. |
| **Imprecision** | 95% CI does not cross the null | 95% CI includes the possibility of both an appreciable association (OR <0.8 or OR >1.2) and crosses the null | 95% CI includes the possibility of both an appreciable harm (OR >1.2) and benefit (OR 0.8) |
| **Publication bias** | No evidence of publication bias on funnel plot or Egger’s test | Evidence of publication bias on funnel plot OR Egger’s test result suggestive of publication bias | Evidence of publication bias on funnel plot AND Egger’s test result suggestive of publication bias |

Table S4b: Definition of GRADE domains which may upgrade level of certainty

|  | **Absent, do not upgrade** | **Present, upgrade one level** |
| --- | --- | --- |
| **Large effect** | OR<2 or >0.5 | OR ≥2 or ≤0.5 and the minimum confounder set have been adjusted for in all studies |
| **Dose-response gradient** | Not applicable as all studies included in GRADE assessments handled exposures as binary categories | |
| **Residual confounding** | There is no residual confounding, or any residual confounding would not be expected to draw the effect estimate towards the null | Residual confounding is anticipated to draw the effect estimate towards the null |

## **Supplementary Table S5: R code for included meta-analyses**

| **HIV Meta-analysis**  ###Meta-analysis - adults only, spirometry only  #Select spirometry only studies  HIV_stratified_analysis_spirometry <- HIV_stratified_analysis %>%  filter(str_detect(odds_ratio_based_on_what_ptld_assessment, regex("spirometry", ignore_case = TRUE)))  #Select only adult studies  HIV_stratified_analysis_spirometry_adults <- HIV_stratified_analysis %>%  filter(str_detect(odds_ratio_based_on_what_ptld_assessment, regex("spirometry", ignore_case = TRUE))) %>%  filter(x1 != "Nkereuwem 2024")  #Run the meta-analysis  hiv_meta_analysis_spiro_adults <- metagen(  TE = log(HIV_stratified_analysis_spirometry_adults$hiv_composite_or),  lower = log(HIV_stratified_analysis_spirometry_adults$hiv_composite_lower_95_ci),  upper = log(HIV_stratified_analysis_spirometry_adults$hiv_composite_upper_95_ci),  studlab = HIV_stratified_analysis_spirometry_adults$x1,  sm = "OR",  method.tau="REML",  HAKN = FALSE,  comb.fixed = FALSE,  comb.random = TRUE,  n.e = HIV_stratified_analysis_spirometry_adults$number_of_participants_included_in_analysis,  data = HIV_stratified_analysis_spirometry_adults)  png("HIV_ma_forest_spiro_adults.png", width = 2000, height = 1400, res = 200)  forest(  hiv_meta_analysis_spiro_adults,  xlim = c(0.01, 100), # Adjust x-axis range  xlab = "Odds Ratio", # Label for x-axis  ref = 1, # Reference line at OR = 1  col.study = "black", # Customize study point color  print.tau2 = FALSE, # Suppress tau² statistic  print.Q = FALSE, # Suppress Q-statistic  print.pval.Q = FALSE, # Suppress Q-statistic p-value  print.I2 = TRUE, # Show I² statistic only  print.I2.ci = TRUE, # Show the I2 CI  smlab = "", # Suppress summary measure label  leftcols = c("studlab","n.e","hiv_prevalence_percent"), # Only display study labels  leftlabs = c("Study","Sample \nsize ", "HIV \n prevalence (%)"),  rightcols = c("effect", "ci") # Only display the odds ratio and confidence interval  )  dev.off()  ##### Funnel plot and Egger's test  png("funnel_hiv.png", width = 2000, height = 1400, res = 200)  HIV_stratified_analysis_spirometry_adults$SE <- (log(HIV_stratified_analysis_spirometry_adults$hiv_composite_upper_95_ci) -  log(HIV_stratified_analysis_spirometry_adults$hiv_composite_lower_95_ci)) / (2 * 1.96)  hiv_meta_analysis_spiro_adults <- rma(yi = log(HIV_stratified_analysis_spirometry_adults$hiv_composite_or), sei = SE, data = HIV_stratified_analysis_spirometry_adults)  funnel(hiv_meta_analysis_spiro_adults,  main = "Funnel Plot for association of abnormal spirometry in adults living with HIV",  cex.main = 0.8,  xlab= "Log Odds Ratio")  dev.off()  eggers_test <- metabias(hiv_meta_analysis_spiro_adults, method.bias = "linreg")  print(eggers_test)  intercept = as.numeric(eggers_test$estimate[1])  se = as.numeric(eggers_test$estimate[2])  llci = intercept - qnorm(0.975) * se  ulci = intercept + qnorm(0.975) * se  ####Stratified by OR adjustment (Sensitivity analysis)  # Split data frame by the `analysis_subgroup` variable  HIV_stratified_groups_spirometry_adults <- split(HIV_stratified_analysis_spirometry_adults, HIV_stratified_analysis_spirometry_adults$analysis_subgroup)  # Apply `metagen()` to each subgroup  hiv_stratified_meta_analysis_spiro_adults <- map(HIV_stratified_groups_spirometry_adults,  ~ metagen(  TE = log(.x$hiv_composite_or),  lower = log(.x$hiv_composite_lower_95_ci),  upper = log(.x$hiv_composite_upper_95_ci),  studlab = .x$x1,  sm = "OR",  comb.fixed = FALSE,  comb.random = TRUE,  method.tau="REML",  HAKN = FALSE,  n.e = .x$number_of_participants_included_in_analysis))  walk2(hiv_stratified_meta_analysis_spiro_adults, seq_along(hiv_stratified_meta_analysis_spiro_adults), function(model, i) {  # Define a unique filename for each subgroup plot  filename <- paste0("HIV_ma_stratified_forest_spiro_adults_", i, ".png")    # Open a PNG device  png(filename, width = 2000, height = 1400, res = 200)    # Generate the forest plot for the current model  forest(model,  xlim = c(0.1,10))    # Close the device to save the plot  dev.off()  }) |
| --- |
| **Undernutrition Meta-analysis**  ### Meta-analysis - spirometry only, adults only  # Select spirometry papers  low_bmi_stratified_analysis_spirometry <- low_bmi_stratified_analysis %>%  filter(str_detect(odds_ratio_based_on_what_ptld_assessment, regex("spirometry", ignore_case = TRUE)))  #Select adult papers. Remove Gupte 2019 (PLoS ONE) as only groups as RSP or AO, not whole abnormal spiro. Remove Allwood 2021 as it only describes AO.  low_bmi_stratified_analysis_spiro_adults <- low_bmi_stratified_analysis_spirometry %>%  filter(x1 != "Nkereuwem 2024",  x1 !="Allwood 2021",  x1 !="Gupte 2019 (PLoS ONE)")  #Run the meta-analysis  low_bmi_meta_analysis_spiro_adults <- metagen(  TE = log(low_bmi_stratified_analysis_spiro_adults$low_bmi_composite_or),  lower = log(low_bmi_stratified_analysis_spiro_adults$low_bmi_composite_lower_95_ci),  upper = log(low_bmi_stratified_analysis_spiro_adults$low_bmi_composite_upper_95_ci),  studlab = low_bmi_stratified_analysis_spiro_adults$x1,  sm = "OR",  method.tau="REML",  HAKN = FALSE,  comb.fixed = FALSE,  comb.random = TRUE,  n.e = low_bmi_stratified_analysis_spiro_adults$number_of_participants_included_in_analysis,  data = low_bmi_stratified_analysis_spiro_adults)  png("low_bmi_ma_forest_spiro_adults.png",width=2000, height=1400, res=200)  low_bmi_ma_forest_sprio_adults <- forest(low_bmi_meta_analysis_spiro_adults,  xlim = c(0.01, 100),  xlab = "Odds Ratio",  ref = 1,  col.study = "black",  print.tau2 = FALSE,  print.Q = FALSE,  print.pval.Q = FALSE,  print.I2 = TRUE,  print.I2.ci = TRUE,  smlab = "",  leftcols = c("studlab","n.e","low_bmi_prevalence_percent"),  leftlabs = c("Study", "Sample \nsize ", "Undernourished \n prevalence (%)"),  rightcols = c("effect", "ci"))  dev.off()  forest(low_bmi_meta_analysis_spiro_adults,  xlim = c(0.01,100))  #calculate the log(OR) and SE for funnel plot  low_bmi_stratified_analysis_spiro_adults$SE <- (log(low_bmi_stratified_analysis_spiro_adults$low_bmi_composite_upper_95_ci) -  log(low_bmi_stratified_analysis_spiro_adults$low_bmi_composite_lower_95_ci)) / (2 * 1.96)  low_bmi_meta_analysis_spiro_adults <- rma(yi = log(low_bmi_stratified_analysis_spiro_adults$low_bmi_composite_or), sei = SE, data = low_bmi_stratified_analysis_spiro_adults)  #Make the funnel plot  png("funnel_undern.png", width=2000, height=1400, res=200)  funnel(low_bmi_meta_analysis_spiro_adults,  main = "Funnel plot for undernutrition in adults meta-analysis",  xlab = "Log Odds Ratio",)  dev.off() |
| **Diabetes Meta-analysis**  ### Meta-analysis - spirometry only (all adult papers anyway)  diabetes_stratified_analysis_spirometry <- diabetes_stratified_analysis %>%  filter(str_detect(odds_ratio_based_on_what_ptld_assessment, regex("spirometry", ignore_case = TRUE)))  #First remove Gupte when doing the overall meta-analysis  diabetes_stratified_analysis_spirometry <-diabetes_stratified_analysis_spirometry %>%  filter(x1 != "Gupte 2019 (PLoS ONE)")    #Then do the meta-analysis  diabetes_meta_analysis_spiro <- metagen(  TE = log(diabetes_stratified_analysis_spirometry$diabetes_composite_or),  lower = log(diabetes_stratified_analysis_spirometry$diabetes_composite_lower_95_ci),  upper = log(diabetes_stratified_analysis_spirometry$diabetes_composite_upper_95_ci),  studlab = diabetes_stratified_analysis_spirometry$x1,  sm = "OR",  method.tau="REML",  HAKN = FALSE,  comb.fixed = FALSE,  comb.random = TRUE,  n.e = diabetes_stratified_analysis_spirometry$number_of_participants_included_in_analysis,  data = diabetes_stratified_analysis_spirometry)  png("diabetes_ma_forest_spiro.png",width=2000, height=1400, res=200)  diabetes_ma_forest_spiro <- forest(diabetes_meta_analysis_spiro,  xlim = c(0.01, 100),  xlab = "Odds Ratio",  ref = 1,  col.study = "black",  print.tau2 = FALSE,  print.Q = FALSE,  print.pval.Q = FALSE,  print.I2 = TRUE,  print.I2.ci = TRUE,  smlab = "",  leftcols = c("studlab","n.e","diabetes_prevalence_percent"),  leftlabs = c("Study", "Sample \nsize ", "Diabetes \n prevalence (%)"),  rightcols = c("effect", "ci"))  dev.off()  forest(diabetes_meta_analysis_spiro)  #calculate the log(OR) and SE for funnel plot  diabetes_stratified_analysis_spirometry$SE <- (log(diabetes_stratified_analysis_spirometry$diabetes_composite_upper_95_ci) -  log(diabetes_stratified_analysis_spirometry$diabetes_composite_lower_95_ci)) / (2 * 1.96)  diabetes_meta_analysis_spiro <- rma(yi = log(diabetes_stratified_analysis_spirometry$diabetes_composite_or), sei = SE, data = diabetes_stratified_analysis_spirometry)  #Then make the funnel plot  png("funnel_diabetes.png", width=2000, height =1400, res=200)  funnel(diabetes_meta_analysis_spiro,  main = "Funnel Plot for Association of Abnormal Spirometry in Diabetes",  cex.main = 0.8,  xlab = "Log Odds Ratio")  dev.off() |

## **Supplementary Table S6: Extended Summary of Included Papers**

| **Paper name** | **Study type** | **Country** | **Sample size**^*^ | **Female (%)** | **TB Diagnosis Method** | **TB Sensitivity** | **Timepoint of Co-morbidity Assessment** | **Co-morbidities Measured (Number: Which)** | **Timepoint of PTLD Assessment** | **PTLD Assessment Tool Used in Analysis** | **Risk of Bias**  Full results shown in table S4 |
| --- | --- | --- | --- | --- | --- | --- | --- | --- | --- | --- | --- |
| Allwood 2023^10^ | X | South Africa | 100 | 42·0 | M | Not stated | At PTLD assessment | 3: N, HIV, Htn | EOT | Echocardiography | Very high |
| Allwood 2021^11^ | X | South Africa | 107 | 38·0 | ATT | Not stated | At PTLD assessment | 5: N, Al, Dr, HIV, RD | ≤ 5 years after EOT | Spirometry and 6MWT | Very high |
| Andrea 2020^12^ | X | Malaysia | 82 | 43·9 | ATT | Not stated | At PTLD assessment | 1: D | ≤ 5 years after EOT | Spirometry | Very high |
| Auld 2021^13^ | PC | South Africa | 92 | 43·0 | M | DS only | TB diagnosis | 1: D | ≤ 1 year after EOT | Spirometry | Very high |
| Chin 2019^14^ | PC | Zimbabwe | 175 | 41·7 | M or C | All | During treatment | 2: HIV, MH | Variable | Composite | High |
| Gandhi 2016^15^ | X^‡^ | India | 146 | 34·2 | Not stated | Not stated | At PTLD assessment | 1: N | EOT | Spirometry | Very high |
| Gupte 2019^16^ | PC | India | 172 | 48·0 | M or C | DS only | TB diagnosis | 4: N, Al, HIV, D | ≤ 1 year after EOT | Spirometry | Some concerns |
| Gupte 2019^17^ | PC | India | 377 | 35·0 | M or C | DS only | TB diagnosis | 3: N, HIV, D | EOT | SGRQ | Very high |
| Khosa 2020^18^ | PC | Mozambique | 62 | 32·3 | M | All | TB diagnosis | 4: N, Al, HIV, An | ≤ 1 year after EOT | Spirometry | Very high |
| Kumar Rai 2020^19^ | PC | India | 128 | 31·2 | M, C or R | Not stated | TB diagnosis | 1: D | EOT | CXR | Very high |
| Lin 2021^20^ | X | China | 115 | 39·0 | Not stated | All | At PTLD assessment | 1: D | EOT | Symptoms, CXR and 6MWT | Very high |
| Lisha 2012^21^ | X | India | 224 | 19·0 | M | DS only | TB diagnosis & PTLD assessment | 1: D | ≤ 5 years after EOT | CXR | Very high |
| Louw 2023^22^ | X | South Africa | 100 | 29·0 | ATT | Not stated | At PTLD assessment | 4: HIV, D, Htn, RD | ≤ 5 years after EOT | Echocardiography | Some concerns |
| Mancuzo 2020^23^ | X | Brazil | 378 | 47·9 | M | Not stated | At PTLD assessment | 8: Al, HIV, D, Htn, RD, K, Ma, Cv | ≤ 5 years after EOT | Spirometry | High |
| Manji 2016^24^ | X | Tanzania | 501 | 39·5 | ATT | Not stated^¶^ | TB diagnosis | 1: HIV | EOT^††^ | Spirometry | High |
| Mbatchou Ngahane 2016^25^ | X | Cameroon | 269 | 45·7 | M plus R | Not stated^¶^ | TB diagnosis | 2: N, HIV | ≤ 5 years after EOT | Spirometry | Very high |
| Meghji 2020^26^† | PC | Malawi | 368 | 32·1 | ATT^§^ | DS only | TB treatment completion | 3: N, HIV, An | ≤ 1 year after EOT | Spirometry and symptoms | Some concerns |
| Mily 2020^27^ | C-C | Bangladesh | 71 | 15·0 | M | DS only | TB diagnosis | 1: D | EOT | CXR | High |
| Mpagama 2021^28^ | X | Tanzania | 219 | 12·0 | M | DS only | Unclear | 3: Al, Dr, HIV | ≤ 5 years after EOT | Composite^‡‡^ | Some concerns |
| Mugo 2018^29^ | X | Kenya | 183 | 42·0 | M | Not stated | At PTLD assessment | 2: N, HIV | ≤ 5 years after EOT | Spirometry | High |
| Namusobya 2023^30^ | X | Uganda | 326 | 44·2 | ATT | All | At PTLD assessment | 2: Al, I | ≤ 5 years after EOT | Symptoms | High |
| Nightingale 2022^31^† | PC | Malawi | 301 | 33·5 | ATT^§^ | DS only | TB treatment completion | 3: N, HIV, RD | ≤ 5 years after EOT | Spirometry | Some concerns |
| Nihues 2015^32^ | X | Brazil | 121 | 47·9 | NR | Not stated | At PTLD assessment | 4: N, Al, Dr, RD | 1-12 years after EOT | Symptoms | High |
| Nkereuwem 2024^33^ | X | The Gambia | 79 | 51·9 | M or C | DS only | At PTLD assessment | 2: N, HIV | EOT | Spirometry | High |
| Nuwagira 2020^34^ | X | Uganda | 95 | 40·0 | NR | MDR only | At PTLD assessment | 1: HIV | ≤ 5 years after EOT | Spirometry | High |
| Osman 2019^35^ | X | South Africa | 51 | 37·0 | M | DS only | TB treatment completion | 1: HIV | ≤ 5 years after EOT | Symptoms | Very high |
| Page 2019^36^ | PC | Uganda | 284 | 38·9 | M or C | Not stated | Unclear | 1: HIV | ≤ 10 years after EOT | Chronic pulmonary aspergillosis^§§^ | Very high |
| Perfura-Yone 2014^37^ | X | Cameroon | 177 | 42·9 | M | DS only | TB treatment completion | 1: HIV | EOT | Symptoms | Very high |
| Pydipalli 2022^38^ | X | India | 118 | 26·3 | ATT | DS only | At PTLD assessment | 3: Al, D, HTN | ≤ 1 year after EOT | Spirometry | Very high |
| Ralph 2013^39^ | PC | Indonesia | 200 | 34·5 | M | All | TB diagnosis | 1: HIV | EOT | SGRQ and 6MWT | Some concerns |
| Ross 2010^40^ | RC | South Africa | 185 | 0·0 | M, C or R | Al | TB diagnosis | 1: HIV | ≤ 5 years after EOT | Spirometry | Some concerns |
| Salzer 2021^41^ | PC | Mozambique | 20 | 36·1 | M | All | TB diagnosis | 1: HIV | EOT | CXR and Aspergillus IgG | Very high |
| Santamaria-Alza 2017^42^ | RC | Columbia | 141 | 34·0 | M | All | TB diagnosis & PTLD assessment | 2: D, Dr | Variable | Imaging (CXR or CT) | Very high |
| Soemarwoto 2021^43^ | C-C | Indonesia | 64 | 39·0 | Not stated | Not stated | Not stated | Multiple^**^ | Not stated | Not stated | Very high |
| Swaminathan 2007^44^ | PC | India | 162 | 14·4 | M | DS only | TB diagnosis | 1: HIV | EOT | CXR | Very high |
| Tandon 2021^45^ | RC | India | 60 | 16·7 | M or C, plus R | DS only | TB diagnosis | 6: Al, D, HTN, An, K, Ma | EOT | CXR | Very high |
| Vashakidze 2019^46^ | X | Georgia | 58 | 43·0 | M | MDR or XDR | At PTLD assessment | 3: N, Al, HCV | ≤ 5 years after EOT | Spirometry and SGRQ | High |
| Wu 2016^47^ | PC | China | 71 | 33·8 | Not stated | Not stated | TB diagnosis | 1: D | EOT | HRCT and Symptoms | Very high |
| Wu 2022^48^ | X | China | 975 | 33·1 | NR | Not stated | At PTLD assessment | 3: Al, RD, Me | ≤ 5 years after EOT | CAT | Very high |
| Zawedde 2024^49^ | X | Uganda | 162 | 45·1 | M or C | DS only | TB diagnosis | 1: HIV | EOT | Spirometry | Very high |
| Zubair 2022^50^ | RC | Pakistan | 321 | 39·5 | M or C | All | Not stated | 1: D | EOT | CXR | Very high |

*Number of participants included in analysis. †Nightingale is the same cohort as Meghji, assessed 2 years later. ‡Reported as case-control but appears cross-sectional. § >77.3% microbiologically proven. ¶ DS treatment described. **Definition of “multiple diseases” - likely TB or destroyed lung accompanied by ≥1 other disease versus none - did not fit our definition of multimorbidity. ††Participants enrolled into study after 20 weeks of anti-TB treatment. ‡‡Symptoms plus either abnormal spirometry or CXR. §§Based on symptoms, CXR changes and positive aspergillus IgG. 6MWT=six-minute walk test, Al=alcohol use disorder, An=anaemia, ATT=received anti-tuberculosis treatment, C=clinical diagnosis of TB, CAT=chronic obstructive pulmonary disease assessment test, C-C=case-control, Cv=cardiovascular disease, CXR=chest X-ray, D=diabetes mellitus, Dr=recreational drug use, DS=drug-sensitive, EOT=end of TB treatment, HCV=chronic hepatitis C infection, HIV=human immunodeficiency virus, HRCT=high-resolution CT, Htn=hypertension, I=immunosuppressive conditions other than HIV, K=chronic kidney disease, M=microbiologically confirmed, Ma=malignancy, MDR=multidrug-resistant TB, Me=“chronic metabolic disorder”, MH=mental health condition, N=nutritional status, NR=registered on national TB registry, PC=prospective cohort, PTLD=post-TB lung disease, R=radiological diagnosis, RC=retrospective cohort, RD=chronic respiratory disease, SGRQ=St. George’s Respiratory Questionnaire, TB=tuberculosis, X=cross-sectional, XDR=extensively drug-resistant TB.

## **Supplementary Table S7: Risk of bias assessment**

A preliminary assessment was conducted to assess if characteristics of the study or study result may have led directly to the result being at very high risk of bias, therefore making a detailed risk-of-bias assessment unnecessary. Where this was the case, the overall risk of bias was marked as very high and no further assessment of bias conducted. For any other result of the preliminary risk assessment, assessors proceeded to the following domains:

| Domain 1: Risk of bias due to confounding |
| --- |
| Domain 2: Risk of bias arising from measurement of the exposure |
| Domain 3: Risk of bias in selection of participants into the study, or the analysis |
| Domain 4: Risk of bias due to post-exposure interventions |
| Domain 5: Risk of bias due to missing data |
| Domain 6: Risk of bias arising from measurement of the outcome |
| Domain 7: Risk of bias in selection of the reported result |

| **Judgement** | **Interpretation** |
| --- | --- |
| *Low risk of bias** | There is little or no concern about bias with regard to this domain |
| *Some concerns* | There is some concern about bias with regard to this domain, although it is not clear that there is an important risk of bias |
| *High risk of bias* | The study has some important problems in this domain: characteristics of the study give rise to a high risk of bias |
| *Very high risk of bias* | The study is very problematic in this domain: characteristics of the study give rise to a very high risk of bias |

Further details regarding ROBINS-E can be found at:

Higgins JPT, Morgan RL, Rooney AA, Taylor KW, Thayer KA, Silva RA, Lemeris C, Akl EA, Bateson TF, Berkman ND, Glenn BS, Hróbjartsson A, LaKind JS, McAleenan A, Meerpohl JJ, Nachman RM, Obbagy JE, O'Connor A, Radke EG, Savović J, Schünemann HJ, Shea B, Tilling K, Verbeek J, Viswanathan M, Sterne JAC. A tool to assess risk of bias in non-randomized follow-up studies of exposure effects (ROBINS-E). *Environment International* 2024 (Apr); **186**: 108602. doi: 10.1016/j.envint.2024.108602.

https://www.riskofbias.info/welcome/robins-e-tool

| **Paper** | **Overall risk of bias** | **Domain 1 (Confounding)** | **Domain 2  (Exposure measurement)** | **Domain 3  (Participant selection)** | **Domain 4  (Post-exp intervention)** | **Domain 5  (Missing data)** | **Domain 6  (Outcome measurement)** | **Domain 7  (Reporting)** |
| --- | --- | --- | --- | --- | --- | --- | --- | --- |
| Allwood 2021^11^ | Very high risk | Low risk | Low risk | Some concerns | Low risk | Very high | Low risk | Low risk |
| Allwood 2023^10^ | Very high risk |  |  |  |  |  |  |  |
| Andrea 2020^12^ | Very high risk |  |  |  |  |  |  |  |
| Auld 2021^13^ | Very high risk |  |  |  |  |  |  |  |
| Chin 2019^14^ | High risk | Low risk | Low risk | High risk | Low risk | Low risk | Low risk | Low risk |
| Gandhi 2016^15^ | Very high risk |  |  |  |  |  |  |  |
| Gupte 2019^17^ | Very high risk |  |  |  |  |  |  |  |
| Gupte 2019^16^ | Some concerns | Low risk | Low risk | Some concerns | Low risk | Low risk | Low risk | Low risk |
| Khosa 2020^18^ | Very high risk |  |  |  |  |  |  |  |
| Kumar Rai 2020^19^ | Very high risk |  |  |  |  |  |  |  |
| Lin 2021^20^ | Very high risk |  |  |  |  |  |  |  |
| Lisha 2012^21^ | Very high risk |  |  |  |  |  |  |  |
| Louw 2023^22^ | Some concerns | Low risk | Low risk | Some concerns | Low risk | Low risk | Low risk | Low risk |
| Mancuzo 2020^23^ | High risk | High risk | Low risk | Some concerns | Low risk | High risk | Low risk | Low risk |
| Manji 2016^24^ | High risk | High risk | Low risk | Some concerns | Low risk | Low risk | Low risk | Low risk |
| Mbatchou Ngahane 2016^25^ | Very high risk | High risk | Low risk | Some concerns | Low risk | Low risk | Low risk | Low risk |
| Meghi 2020^26^ | Some concerns | Low risk | Low risk | Low risk | Low risk | Some concerns | Low risk | Low risk |
| Mily 2020^27^ | High risk | High risk | Low risk | Low risk | Low risk | Low risk | Low risk | Low risk |
| Mpagama 2021^28^ | Some concerns | Low risk | Low risk | Some concerns | Low risk | Low risk | Low risk | Low risk |
| Mugo 2018^29^ | High risk | Low risk | Low risk | High risk | Low risk | Low risk | Low risk | Low risk |
| Namusobya 2023^30^ | High risk | Low risk | Some concerns | Some concerns | Low risk | Low risk | Some concerns | Low risk |
| Nightingale 2022^31^ | Some concerns | Low risk | Low risk | Some concerns | Low risk | Some concerns | Low risk | Low risk |
| Nihues 2015^32^ | High risk | Some concerns | Low risk | High risk | Low risk | Low risk | Low risk | Low risk |
| Nkereuwem 2023^33^ | High risk | High risk | Low risk | Some concerns | Low risk | Low risk | Low risk | Low risk |
| Nuwagira 2020^34^ | High risk | High risk | Low risk | Some concerns | Low risk | Low risk | Low risk | Low risk |
| Osman 2019^35^ | Very high risk |  |  |  |  |  |  |  |
| Page 2019^36^ | Very high risk |  |  |  |  |  |  |  |
| Perfura-Yone 2014^37^ | Very high risk |  |  |  |  |  |  |  |
| Pydipalli 2022^38^ | Very high risk |  |  |  |  |  |  |  |
| Ralph 2013^39^ | Some concerns | Some concerns | Low risk | Some concerns | Low risk | Some concerns | Low risk | Low risk |
| Ross 2010^40^ | Some concerns | Low risk | Low risk | Some concerns | Low risk | Low risk | Low risk | Low risk |
| Salzer 2021^41^ | Very high risk |  |  |  |  |  |  |  |
| Santamaria-Alza 2017^42^ | Very high risk |  |  |  |  |  |  |  |
| Soemwaroto 2021^43^ | Very high risk |  |  |  |  |  |  |  |
| Swaminathan 2007^44^ | Very high risk |  |  |  |  |  |  |  |
| Tandon 2021^45^ | Very high risk |  |  |  |  |  |  |  |
| Vashakidze 2019^46^ | High risk | High risk | Low risk | High risk | Low risk | Low risk | Low risk | Low risk |
| Wu 2016^47^ | Very high risk |  |  |  |  |  |  |  |
| Wu 2022^48^ | Very high risk | High risk | Some concerns | High risk | Low risk | Low risk | High risk | Low risk |
| Zawedde 2024^49^ | Very high risk |  |  |  |  |  |  |  |
| Zubair 2022^50^ | Very high risk |  |  |  |  |  |  |  |

## **Supplementary Figure S1: Geographical distribution of papers by WHO region**


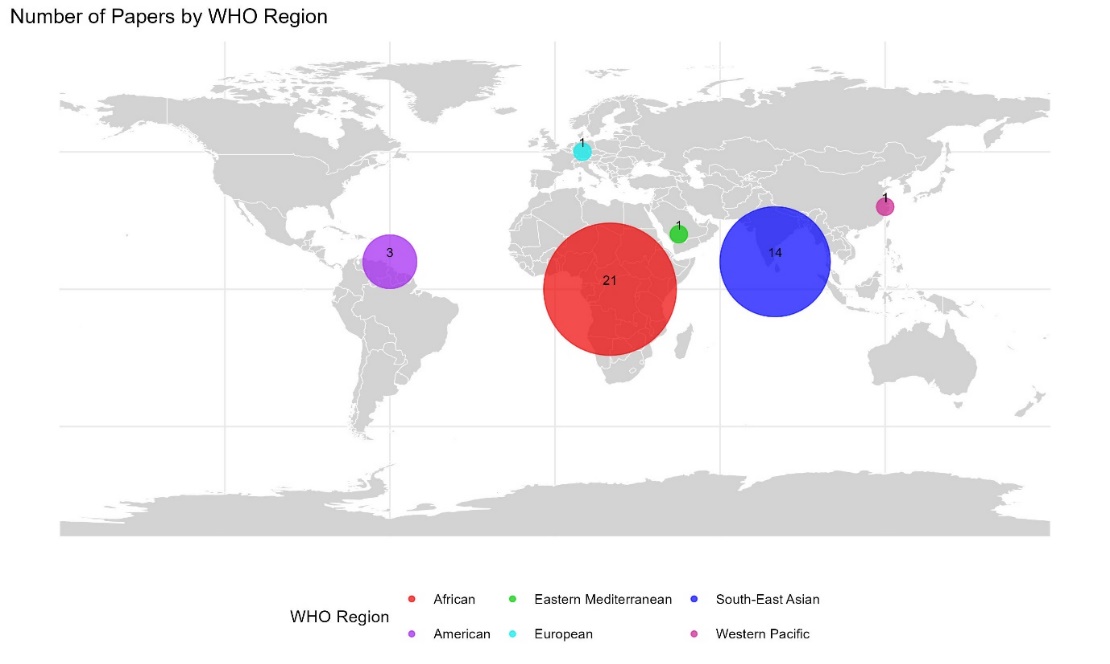


## **Supplementary Table S8: Summary of included HIV papers**

| **Study Name** | **Study Type** | **Country** | **Sample Size** | **TB Drug Sensitivity** | **Current Smoker (%)** | **HIV Prevalence (%)** | **Timepoint of PTLD Assessment** | **PTLD Assessment Tool Used** | **Analysis** | Risk of Bias |
| --- | --- | --- | --- | --- | --- | --- | --- | --- | --- | --- |
| Allwood 2023^10^ | Cross-sectional | South Africa | 100 | Not stated | 15.0 | 46.0 | EOT | Echo | uOR 2.355 (0.08 to 71.9) | Very high |
| Allwood 2021^11^ | Cross-sectional | South Africa | 107 | Not stated | 59.0 | 17.0 | ≤5 years after EOT | Spirometry  6MWT | FEV1/FVC below LLN uOR 1.34 (0.42 to 4.01)  6MWT adjusted coefficient estimate (95% CI): 49.55 (9.31 to 89.79), p value 0.02 | Very high |
| Chin 2019^14^ | Prospective cohort | Zimbabwe | 175 | All | 5.7 | 64.6 | Variable | Composite^b^ | Adjusted Relative Risk (aRR) = 0.6 (0.3 to 1.3) | High |
| Gupte 2019 ^16^ | Prospective cohort | India | 172 | DS only | Not stated  (20% ever) | 4.1 | ≤1 year after EOT | Spirometry | Median FEV1 (% predicted): +HIV 73 (IQR 39 to 87) vs -HIV 70 (IQR 58 to 81), p value 0.71  Median FVC (% predicted): +HIV 71 (IQR 44 to 96) vs -HIV 74 (IQR 64 to 85), p value 0.71  Median PEFR (% predicted): +HIV 93 (IQR 59 to 111) vs -HIV 73 (IQR 56 to 90), p value 0.14 | Some concerns |
| Gupte 2019 ^17^ | Prospective cohort | India | 377 | DS only | 14.0 | 4.0 | EOT | SGRQ | Median total SGRQ +HIV 9 (IQR 3 to 19) vs -HIV 7 (IQR 2 to 23), p value = 0.75  % change in SGRQ +HIV -69 (IQR -119 to -20) vs -HIV -63 (-68 to -58), p value = 0.59 | Very high |
| Khosa 2020^18^ | Prospective cohort | Mozambique | 62 | All | Not stated (35.5% ever) | 62.9 | ≤1 year after EOT | Spirometry | uOR 1.29 (0.441 to 3.748) | Very high |
| Louw 2023^22^ | Cross-sectional | South Africa | 100 | Not stated | 72.0 | 10.0 | ≤5 years after EOT | Echo | uOR 3.67 (0.54 to 18.55), aOR 2.23 (0.28 to 17.9) | Some concerns |
| Mancuzo 2020^23^ | Cross-sectional | Brazil | 378 | Not stated | 18.8 | 4.6 | ≤5 years after EOT | Spirometry | uOR 2.17 (0.23 to 20.67) | High |
| Manji 2016^24^ | Cross-sectional | Tanzania | 501 | Not stated^a^ | Not stated  (29.3% ever) | 30.3 | EOT | Spirometry | uOR 0.61 (0.40 to 0.93), aOR 0.61 (0.08 to 0.95) | High |
| Mbatchou Ngahane 2016^25^ | Cross-sectional | Cameroon | 269 | Not stated^a^ | 2.2 | 17.8 | ≤5 years after EOT | Spirometry | uOR 0.92 (0.49 to 1.73) | Very high |
| Meghji 2020^26^ | Prospective cohort | Malawi | 368 | DS only | Not stated  (29.6% ever) | 60.5 | ≤1 year after EOT | Spirometry  Symptoms  HRCT | All uOR (95% CI):  Abnormal spirometry=0.49 (0.31 to 0.79), Obstruction = 0.629 (0.357 to 1.109), Restriction = 0.488 (0.256 to 0.931)  Symptoms at 1 year = 0.33 to 0.40 (0.18 to 0.98) (across CD4 groups and models)  At least one respiratory event = 0.33 to 0.43 (0.13 to 0.90) (across CD4 groups and models)  mMRC grade >=2 = 0.742 (0.432 to 1.276) | Some concerns |
| Mpagama 2021^28^ | Cross-sectional | Tanzania | 219 | DS only | 47.0 | 16.0 | ≤5 years after EOT | Composite^c^ | uOR 0.6 (0.3 to 1.2), aOR 1.10 (0.4 to 2.9) | Some concerns |
| Mugo 2018^29^ | Cross-sectional | Kenya | 183 | Not stated | Not stated  (22% ever) | 33.0 | ≤5 years after EOT | Spirometry | uOR 0.43 (0.20 to 0.92), aOR 0.5 (0.2 to 0.6) | High |
| Nightingale 2022^e31^ | Prospective cohort | Malawi | 301 | DS only | Not stated (28.9% ever) | 60.4 | ≤5 years after EOT | Spirometry | Unadjusted linear mixed effects regression coefficients:  FEV1 (L) = 0.185 (0.058 to 0.312), FVC (L) = 0.177 (0.031 to 0.323) | Some concerns |
| Nkereuwem 2024^33^ | Cross-sectional | The Gambia | 79 | DS only | Not stated (2.5% ever) | 10.1 | EOT | Spirometry | uOR 0.9 (0.8 to 1.1) | High |
| Nuwagira 2020^34^ | Cross-sectional | Uganda | 95 | MDR only | 1.0 | 58.0 | ≤5 years after EOT | Spirometry | aOR 0.52 (0.17 to 1.62) | High |
| Osman 2019^35^ | Cross-sectional | South Africa | 51 | DS only | Not stated (63% ever) | 49.0 | ≤5 years after EOT | Symptoms | uOR: Cough = 0.64 (0.18 to 2.20), Severe chest illness last 12 months = 1.14 (0.34 to 3.82).  mMRC grade 2 or more =0.92 (0.31 to 2.77) | Very high |
| Page 2019^36^ | Prospective cohort | Uganda | 284 | Not stated | 39.0 | 50.0 | ≤10 years after EOT | Chronic pulmonary aspergillosis^d^ | uOR 0.43 (0.13 to 1.40) | Very high |
| Perfura-Yone 2014^37^ | Cross-sectional | Cameroon | 177 | DS only | Not stated (19.8% ever) | 26.6 | EOT | Symptoms | uOR 0.76 (0.39 to 1.51) | Very high |
| Ralph 2013^39^ | Prospective cohort | Indonesia | 200 | All | Not stated (55% ever) | 13.0 | EOT | SGRQ  6MWT | P values from multivariable logistic regression given:  Median SGRQ +HIV 15.8 (IQR 7.01 to 29.2) vs -HIV 3.25 (IQR 1.72 to 7.18), p value = 0.02  Median 6MWT +HIV 440m (IQR 420 to 475) vs -HIV 480m (IQR 425 to 520), p value = 0.6 | Some concerns |
| Ross 2010^40^ | Retrospective cohort | South Africa | 185 | All | 47.5 | 52.9 | ≤5 years after EOT | Spirometry | Mean height adjusted decline in FEV1 (ml/year) +HIV vs -HIV: 3.1 (-21.2 to 27.4)  Mean height-adjusted decline in FVC (ml/year) +HIV vs -HIV: -16.2 (-41.7 to 9.4) | Some concerns |
| Salzer 2021^41^ | Prospective cohort | Mozambique | 20 | All | Not stated | 65.6 | EOT | CXR  Aspergillus-specific IgG | Residual cavitary lesions in 5 +HIV vs 4 -HIV patients.  Median Aspergillus-specific IgG antibody level +HIV = 7.55 mg/L (IQR 2.05-20.6) vs -HIV 16.1 mg/L (IQR 3.09-174.0) | Very high |
| Swaminathan 2007^44^ | Prospective cohort | India | 162 | DS only | Not stated | 49.4 | EOT | CXR | uOR 0.016 (0.0022 to 0.1220) | Very high |
| Zawedde 2024^49^ | Cross-sectional | Uganda | 162 | DS only | Not stated (15.4% ever) | 40.1 | EOT | Spirometry  SGRQ | Abnormal spirometry: uOR 0.64 (0.34 to 1.23)  Median SGRQ +HIV 4 (IQR 0 to 16) vs -HIV 5 (IQR 0 to 20), p value = 0.49 | Very high |

^a^DS treatment described. ^b^ 1. Radiographic evidence of volume loss, bronchiectasis, fibrosis or hyperexpansion;and 2. Respiratory symptoms (CAT>= 10) and/or at least two respiratory exacerbation in the previous 12 months;and 3. Atleast one of i. spirometry abnormality, ii. ISWT <50% predicted, or iii. oxygen desaturation to <=88% during exercise testing.^c^ Symptoms plus either abnormal spirometry or CXR. ^d^Based on symptoms, CXR changes and positive aspergillus IgG. ^e^Same cohort as Meghji 2020 but assessed 2 years later. 6MWT = six-minute walk test, aOR = adjusted odds ratio, CXR = chest x-ray, DS = drug-sensitive, EOT = end of treatment, FEV1 = forced expiratory volume in 1 second, FVC = forced vital capacity, HRCT = high-resolution CT, IgG = immunoglobulin G, IQR = inter-quartile range, LLN = lower limit of normal, MDR = multi-drug resistant, mMRC = modified medical research council dyspnoea scale, PEFR = peak expiratory flow rate, SE = standard error, SGRQ = St.George's Respiratory Questionnaire, uOR = unadjusted odds ratio. Unless stated otherwise, numbers in brackets after odds ratios are 95% confidence intervals. Stated study sample size is number of participants included in the analysis.

## **Supplementary Table S9: Summary of included diabetes papers**

| **Study Name** | **Study Type** | **Country** | **Sample Size** | **TB Drug Sensitivity** | **Current Smokers (%)** | **Definition of Diabetes Diagnosis** | **Diabetes Prevalence (%)** | **Timepoint of PTLD Assessment** | **PTLD Assessment Tool** | **Analysis** | **Risk of Bias** |
| --- | --- | --- | --- | --- | --- | --- | --- | --- | --- | --- | --- |
| Andrea 2020^12^ | Cross-sectional | Malaysia | 82 | Not stated | Not stated  (35.4% ever smokers) | Pre-existing diagnosis | 30.5 | ≤5 years after EOT | Spirometry | uOR 1.71 (0.66 to 4.44) | Very high |
| Auld 2021^13^ | Prospective cohort | South Africa | 92 | DS only | 16 | HbA1c prior to ART initiation | Not stated  Median HbA1C = 6.1 (IQR 5.8 - 6.4) | ≤1 year after EOT | Spirometry | uOR 1.13 (0.76 to 1.69) with each category increase in baseline HbA1C (grouped into <5.7, 5.7-5.9, 6.0-6.2, ≥6.3) | Very high |
| Gupte 2019^16^ | Prospective cohort | India | 172 | DS only | Not stated  (20% ever smokers) | Pre-existing diagnosis or HbA1c ≥6.5% at ATT initiation | 13 | ≤1 year after EOT | Spirometry | Airway obstruction aOR 0.67 (0.16 to 2.69)  Restrictive spirometry pattern aOR 4.06 (1.14 to 14.43) | Some concerns |
| Gupte 2019^17^ | Prospective cohort | India | 377 | DS only | 14 | Pre-existing diagnosis or HbA1c ≥6.5% at ATT initiation | 25 | EOT | SGRQ | Median (IQR):  Total SGRQ score DM 10 (2–19) vs no DM 8 (3-20), p-value 0.49  Change in SGRQ % DM -60 (-69 to -51) vs -65 (-72 to -58), p-value 0.46 | Very high |
| Kumar Rai 2020^19^ | Prospective cohort | India | 128 | Not stated | 6.25 | Not stated | 3.12 | EOT | CXR | uOR 1.18 (0.12 to 11.73) | Very high |
| Lin 2021^20^ | Cross-sectional | China | 115 | All:  MDR 6 (5%) | 10 | Pre-existing diagnosis | 19.1 | EOT | Symptoms  CXR  6MWT | Symptoms: continuation more common in DM patients (p=0.027)  CXR: no difference in proportion of patients with CXR abnormalities p-value 0.52  6MWT: "No significant difference"^b^ | Very high |
| Lisha 2012^21^ | Cross-sectional | India | 224 | DS only | Not stated | Pre-existing diagnosis | 10.2 | ≤5 years after EOT | CXR | Diabetes not association with radiological sequelae p-value 0.107 | Very high |
| Louw 2023^22^ | Cross-sectional | South Africa | 100 | Not stated | 72 | Pre-existing diagnosis | 16 | ≤5 years after EOT | Echocardiography | uOR 1.57 (0.30 to 8.36) | Some concerns |
| Mancuzo 2020^23^ | Cross-sectional | Brazil | 378 | Not stated | 18.8 | Pre-existing diagnosis | 10.9 | ≤5 years after EOT | Spirometry | uOR1.92 (0.50 to 7.35) | High |
| Mily 2020^27^ | Case-control | Bangladesh | 71 | DS only | Not stated | Pre-existing diagnosis | Case-control | EOT | CXR | Mean difference in % whole lung involvement -1.00 (95% CI -4.72 to 2.72)  Multivariable regression^a^ p value 0.584. | High |
| Pydipalli 2022^38^ | Cross-sectional | India | 118 | DS only | Not stated  (61% ever smokers) | Pre-existing diagnosis | 50 | ≤1 year after EOT | Spirometry | uOR 1.55 (0.73 to 3.28) | Very high |
| Santamaria-Alza 2017^42^ | Retrospective cohort | Columbia | 141 | All | 46 | Pre-existing diagnosis | 5 | Variable | Imaging (CXR or CT) | uOR 0.29 (0.05 to 1.65) | Very high |
| Tandon 2021^45^ | Retrospective cohort | India | 60 | DS only | 40 | Pre-existing diagnosis | 11.7 | EOT | CXR | uOR 0.75 (0.15 to 3.67) | Very high |
| Wu 2016^47^ | Prospective cohort | China | 71 | Not stated | Not stated | Pre-existing diagnosis OR  RBS ≥11.1/FBS ≥7.0mmol/L and commenced on anti-diabetic medication | 42.3 | EOT | HRCT  Symptoms | HRCT uOR:  Consolidation = 6.14 (2.15 to 17.51),  Bronchiectasis = 0.70 (0.27 to 1.82),  Cavitation = 7.33 (1.83 to 29.45)  No difference according to specific symptoms (all p-values >0.05):  Fever = DM 67.7% vs no DM 63.4%,  Cough = DM 83.3% vs no DM 80.5%,  Sputum = DM 53.3% vs no DM 65.9%,  Dsypnoea = DM 33.3% vs no DM 46.3% | Very high |
| Zubair 2022^50^ | Retrospective cohort | Pakistan | 321 | All | Not stated | Pre-existing diagnosis | 11.8 | EOT | CXR | uOR 0.75 (0.32 to 1.73) | Very high |

^a^Adjusted for age, sex, baseline BMI, SES score, BCG status and baseline lung involvement. ^b^Unclear in paper text if “significant” referred to clinical or statistical significance. 6MWT = six-minute walk test, ART = anti-retroviral therapy, aOR = adjusted odds ratio, ATT = anti-tuberculosis therapy, CXR = chest x-ray, DS = drug-sensitive, EOT = end of treatment, FBS = fasting blood glucose, HbA1c = glycated haemaglobin, HRCT = high-resolution CT, IQR = inter-quartile range, MDR = multi-drug resistant, RBS = random blood glucose, SGRQ = St.George's Respiratory Questionnaire, uOR = unadjusted odds ratio. Unless stated otherwise, numbers in brackets after odds ratios are 95% confidence intervals. Stated study sample size is number of participants included in the analysis

## **Supplementary Table S10: Summary of included nutritional status papers**

| **Study Name** | **Study Type** | **Country** | **Sample Size** | **TB Drug Sensitivity** | **Current smokers (%)** | **Nutrition Category Definition** | **Nutrition Category**  **Prevalence (%)** | **Timepoint of PTLD Assessment** | **PTLD Assessment Tool** | **Analysis** | **Risk of Bias** |
| --- | --- | --- | --- | --- | --- | --- | --- | --- | --- | --- | --- |
| CONTINUOUS BMI PAPERS – all reported analyses are for each unit increase in BMI | | | | | | | | | | |  |
| Allwood 2023^10^ | Cross-sectional | South Africa | 100 | Not stated | 15 | - | Not stated. Mean BMI 24.3 (SD 5.2) | EOT | Echocardiography | aOR 0.76 (0.54 to 2.44) | Very high |
| Allwood 2021^11^ | Cross-sectional | South Africa | 107 | Not stated | 59 | - | Not stated. Median BMI 20.7 (IQR 18.5 - 25.6) | ≤5 years after EOT | Spirometry  6MWT | FEV1/FVC below LLN: uOR 1.10 (1.01 to 1.20), aOR 1.14 (1.04 to 1.27)  6MWT distance: unadjusted coefficient -4.72 (-7.20 to -2.23), adjusted coefficient -4.30 ( -6.61 to -1.99) | Very high |
| Meghji 2020^26^ | Prospective cohort | Malawi | 368 | DS only | Not stated (29.6% ever) | - | Not stated. Median BMI 20.5 (IQR 19.0 - 22.3) | ≤1 year after EOT | Spirometry  Symptoms | FEV (absolute, in ml) 18.32 (9.26 to 27.38)  FVC (absolute, in ml) 40.58 (29.97 to 51.19)  Respiratory symptoms at 1 year: uOR 1.01 (0.93 to 1.10)  Acute respiratory event at 1 year: uOR0.98 (0.89 to 1.09) | Some concerns |
| Nightingale 2022^c31^ | Prospective cohort | Malawi | 301 | DS only | Not stated (28.8% ever) | - | Not stated. Median BMI 20.5 (IQR 19 – 22.3) | ≤5 years after EOT | Spirometry | Linear mixed effect regression co-efficients:  FEV1 (L)= 0.093 (0.031 to 0.155), FVC (L) = 0.127 (0.056 to 0.199) | Some concerns |
| Nihues 2015^32^ | Cross-sectional | Brazil | 100 | Not stated | 36 | - | Not stated | 1-12 years after EOT | Symptoms | uOR 1.04 (0.95 to 1.14) | High |
| Vashakidze 2019^46^ | Cross-sectional | Georgia | 58 | MDR or XDR only | 40 | - | Not stated. Median BMI 23.9 (IQR 21-27) | ≤5 years after EOT | Spirometry  SGRQ | Linear regression co-efficients:  FEV1% predicted -0.09, FVC% pred -0.39, FEV1/FVC 0.17  SGRQ score 0.57, p-values all >0.05 | High |
| UNDERNUTRITION PAPERS | | | | | | | | | | |  |
| Gandhi 2016^15^ | Cross-sectional^b^ | India | 146 | Not stated | 28 | Not specified | 58 | EOT | Spirometry | uOR 1.31 (0.62 to 2.75) | Very high |
| Gupte 2019^16^ | Prospective cohort | India | 172 | DS only | Not stated  (20% ever) | BMI <18.5 | 51 | ≤1 year after EOT | Spirometry | Airflow obstruction:  BMI 16-18.5 : aOR 1.55 (0.58 to 4.09), BMI <16 : aOR 1.71 (0.57 to 5.10)  Restrictive spirometry pattern:  BMI 16-18.5 : aOR 0.46 (0.19-1.11), BMI <16 : aOR 1.61 (0.60-4.30) | Some concerns |
| Gupte 2019^17^ | Prospective cohort | India | 377 | DS only | 14 | BMI <18.5 | 56 | EOT | SGRQ | Median SGRQ:  BMI <18.5: 10 (4-22), BMI 18.5-25: 6 (2-17), BMI≥: 10 (4-22), p value 0.04.  Change in SGRQ:  BMI <18.5:-62 (-70 to -54), BMI 18.5-25: -68 (-76 to -61) and BMI≥25: -51 (-73 to -28), p value 0.29 | Very high |
| Khosa 2020^18^ | Prospective cohort | Mozambique | 62 | All | Not stated (35.5% ever) | BMI <18.5 | 42 | ≤1 year after EOT | Spirometry | uOR 1.43 (0.49 to 4.17) | Very high |
| Mugo 2018^29^ | Cross-sectional | Kenya | 183 | Not stated | Not stated (22% ever) | BMI <18.5 | 17 | ≤5 years after EOT | Spirometry | uOR 5.96 (2.64 to 13.44), aOR 3.33 (1.7 to 5.0) | High |
| Nkereuwem 2024^33^ | Cross-sectional | The Gambia | 79 | DS only | Not stated (2.5% ever) | BMI-for-age Z score < -2SD | 26% "underweight" | EOT | Spirometry | uOR 4.60 (1.4 to 15.2), aOR 8.30 (2.0 to 35.2) | High |
| OVERWEIGHT/OBESITY PAPERS | | | | | | | | | | |  |
| Gandhi 2016^15^ | Cross-sectional^b^ | India | 146 | Not stated | 28.1 | “Overnourished” not specified | 6.85 | EOT | Spirometry | “Overnourished” uOR 0.2 (0.055 to 0.772) | Very high |
| Mbatchou Ngahane 2016^25^ | Cross-sectional | Cameroon | 269 | Not stated^a^ | 2.2 | Overweight, BMI ≥ 25.0  Obese, BMI ≥ 30.0 | Overweight: 22.3%, Obese: 11.5% | ≤ 5 years after EOT | Spirometry | Obese uOR 0.7 (0.41 to 1.17) | Very high |
| Mugo 2018^29^ | Cross-sectional | Kenya | 183 | Not stated | Not stated (22% ever) | Overweight, BMI ≥ 25.0 | 15.85 | ≤ 5 years after EOT | Spirometry | Overweight uOR 0.5 (0.165 to 1.596) | High |

^a^ DS treatment described. ^b^Reported as case-control but appears cross-sectional. ^c^ Same cohort as Meghji 2020 but assessed 2 years later. 6MWT = six-minute walk test, aOR = adjusted odds ratio, BMI = body mass index, CXR = chest x-ray, DS = drug-sensitive, EOT = end of treatment, FEV1 = forced expiratory volume in 1 second, FVC = forced vital capacity, HRCT = high-resolution CT, IgG = immunoglobulin G, IQR = inter-quartile range, LLN = lower limit of normal, MDR = multi-drug resistant, mMRC = modified medical research council dyspnoea scale, PEFR = peak expiratory flow rate, SD = standard deviation, SE = standard error, SGRQ = St.George's Respiratory Questionnaire, uOR = unadjusted odds ratio, XDR = extensively drug-resistant TB. Unless stated otherwise, numbers in brackets after odds ratios are 95% confidence intervals. Stated study sample size is number of participants included in the analysis.

## **Supplementary Table S11: Summary of included Alcohol Papers**

| **Study Name** | **Study Type** | **Country** | **Sample Size** | **TB Drug Sensitivity** | **Current Smokers (%)** | **Alcohol Use Category Definition** | **Alcohol Category Prevalence (%)** | **Timepoint of PTLD Assessment** | **PTLD Assessment Tool** | **Analysis** | **Risk of Bias** |
| --- | --- | --- | --- | --- | --- | --- | --- | --- | --- | --- | --- |
| ALCOHOL USE DISORDER | | | | | | | | | | |  |
|  |  |  |  |  |  |  |  |  |  |  |  |
| Gupte 2019^16^ | Prospective cohort | India | 172 | DS only | Not stated (20% ever) | AUDIT of 8 or more | 22.0 | ≤ 1 year after EOT | Spirometry | % predicted median (IQR) for alcohol use disorder vs no alcohol use disorder:  FEV1 (%) 69 (51 to 80) vs 70 (59 to 82), p-value 0.59  FVC (%) 74 (64 to 85) vs 72 (64 to 85), p-value 0.63  PEFR (%) 57 (44 to 75) vs 78 (60 to 93), p-value 0.003 | Some concerns |
| Khosa 2020^18^ | Prospective cohort | Mozambique | 62 | All | Not stated (35.5% ever) | Critical alcohol consumption:  60g (30g women) per occasion of alcohol drinking  and/or >150g (80g women) per week | 53.3 | ≤ 1 year after EOT | Spirometry | uOR 0.6 (0.219 to 1.794) | Very high |
| Mancuzo 2020^23^ | Cross-sectional | Brazil | 378 | Not stated | 18.8 | CAGE assessment, exact definition not given | 27.0 | ≤ 5 years after EOT | Spirometry | uOR 0.93 (0.30 to 2.91) | High |
| Namusobya 2023^30^ | Cross-sectional | Uganda | 326 | All | Not stated (20.2% ever) | "Alcoholism" not defined further | 44.5 | ≤ 5 years after EOT | Symptoms | aOR 0.26 (0.12 to 0.57) | High |
| Nihues 2015^32^ | Cross-sectional | Brazil | 100 | Not stated | 35.5 | "Alcoholism" not defined further | 19.8 | 1 - 12 years after EOT | Symptoms | uOR 2.4 (0.94 to 5.95), aOR 3.1 (1.16 to 8.30) | High |
| Tandon 2021^45^ | Retrospective cohort | India | 60 | DS only | 40 | "Alcohol addiction" - not further defined | 18.3 | EOT | CXR | uOR 0.3 (0.08 to 1.39) | Very high |
| Wu 2022^48^ | Cross-sectional | China | 975 | Not stated | 13.7 | Drinks = 21 times/week for more than 6 consecutive months  (vs doesn't drink or quit drinking) | 10.2 | ≤ 5 years after EOT | CAT | Mean CAT scores out of 40 (±SD):  Drinks alcohol = 13.14 ± 6.10,  Doesn't drink = 13.29 ± 6.10,  Quit drinking = 14.27 ± 5.44. p value 0.29. | Very high |
| ANY ALCOHOL CONSUMPTION | | | | | | | | | | |  |
| Allwood 2021^11^ | Cross-sectional | South Africa | 107 | Not stated | 59 | Consumed alcohol yes/no, also unit of alcohol consumed/week | 49.0 | ≤ 5 years after EOT | Spirometry,  6MWT | FEV1/FVC below LLN:  uOR 0.70 (0.30 to 1.51), aOR 0.85 (0.17 to 4.25)  % predicted FVC:  adjusted linear regression coefficient 9.22 (3.06 to 15.38)  6MWT distance:   adjusted linear regression coefficient -23.30 (-51.05 to 4.45) | Very high |
| Mpagama 2021^28^ | Cross-sectional | Tanzania | 219 | DS only | 47 | Drinks alcohol | 59.0 | ≤ 5 years after EOT | Composite: spirometry, symptoms and CXR | uOR 1.8 (1.0 to 3.2), aOR 1.4 (0.6 to 2.9) | Some concerns |
| Pydipalli 2022^38^ | Cross-sectional | India | 118 | DS only | Not stated (61.0% ever) | History of alcohol use in the past year | 44.9 | ≤ 1 year after EOT | Spirometry | uOR 0.70 (0.34 to 1.53) | Very high |
|  |  |  |  |  |  |  |  |  |  |  |  |
| Vashakidze 2019^46^ | Cross-sectional | Georgia | 58 | MDR or XDR only | 40 | "Alcohol use" - not further defined | 19.0 | ≤ 5 years after EOT | Spirometry, SGRQ | Linear regression coefficients:  FEV1% pred -17.22, p-value >0.05  FVC% pred -13.89, p-value >0.05  FEV1/FVC -8.81, p-value between 0.01 to 0.05  SGRQ score -8.76, p-value >0.05. P values are reporting style of the paper. | High |
|  |  |  |  |  |  |  |  |  |  |  |  |

6MWT = six-minute walk test, aOR = adjusted odds ratio, AUDIT = alcohol use disorders identification test, CAT = COPD assessment test, CAGE = Cut down Annoyed Guilty Eye-opener alcohol screening tool, CXR = chest x-ray, DS = drug-sensitive, EOT = end of treatment, FEV1 = forced expiratory volume in 1 second, FVC = forced vital capacity, HRCT = high-resolution CT, IQR = inter-quartile range, LLN = lower limit of normal, MDR = multi-drug resistant, PEFR = peak expiratory flow rate, SGRQ = St.George's Respiratory Questionnaire, uOR = unadjusted odds ratio, XDR = extensively drug-resistant TB. Unless stated otherwise, numbers in brackets after odds ratios are 95% confidence intervals. Stated study sample size is number of participants included in the analysis.

## **Supplementary Table S12: Summary of other included papers**

| **Study Name** | **Study Type** | **Country** | **Sample Size** | **TB Drug Sensitivity** | **Current smokers (%)** | **Co-morbidity Definition, if stated** | **Co-morbidity Prevalence (%)** | **Timepoint of PTLD Assessment** | **PTLD Assessment Tool(s)** | **Analysis** | **Risk of Bias** |
| --- | --- | --- | --- | --- | --- | --- | --- | --- | --- | --- | --- |
| **Recreational Drug Use Papers** | | | | | | | | | | |  |
| Allwood 2021^11^ | Cross-sectional | South Africa | 107 | Not stated | 59.0 | Smoked subtance other than cigarettes^a^ | 35.0 | ≤ 5 years after EOT | Spirometry, 6MWT | Airway obstruction: uOR 1.79 (0.78 to 4.13)  6MWT distance: adjusted linear regression coefficient 23.91 (–7.41 to 55.23) | Very high |
| Mpagama 2021^28^ | Cross-sectional | Tanzania | 219 | DS only | 47.0 | Smoked subtance other than cigarettes^b^ | 22.0 | ≤ 5 years after EOT | Composite: spirometry, symptoms and CXR | uOR 1.5 (0.8 to 2.9), aOR 0.8 (0.3 to 2.0) | Some concerns |
| Nihues 2015^32^ | Cross-sectional | Brazil | 121 | Not stated | 35.5 | Not defined | 10.7 | 1-12 years after EOT | Symptoms | uOR 2.22 (0.75 to 6.56) | High |
| Santamaria-Alza 2017^42^ | Retrospective cohort | Columbia | 141 | All | 46.0 | Pharmacodependence, cocaine inhalation | 18.0 | Variable | Imaging (CXR or CT) | Pharmacodependence more frequent in those with lesions | Very high |
| **Respiratory Condition Papers** | | | | | | | | | | |  |
| Allwood 2021^11^ | Cross-sectional | South Africa | 107 | Not stated | 59 | Any^c^ | 2.8 | ≤ 5 years after EOT | Spirometry, 6MWT | FVC% predicted: unadjusted linear regression coefficient = -9.61 (-21.86 to 2.64) 6MWT distance: unadjusted linear regression coefficient = -26.76 (-90.64 to 37.13) | Very high |
| Louw 2023^22^ | Cross-sectional | South Africa | 100 | Not stated | 72 | Asthma and COPD combined | 5.0 | ≤ 5 years after EOT | Echocardiography | No PH = 5 (5.5%) vs probable PH = 0 (0%), p value=1.0 | Some concerns |
| Mancuzo 2020^23^ | Cross-sectional | Brazil | 378 | Not stated | 19 | History of lung disease^d^ or smoking prior to TB treatment | 46.0 | ≤ 5 years after EOT | Spirometry | Abnormal spirometry: uOR 2.20 (1.42 to 3.38) mMRC score 2-4 = uOR 2.65 (1.32 to 5.32) | High |
| Nightingale 2022^31^ | Prospective cohort | Malawi | 301 | DS only | Ever: 28.8 | "Self-reported respiratory condition prior to TB diagnosis" | 43.3 | ≤ 5 years after EOT | Spirometry | Linear mixed effects regression coefficient: FEV1(L) = -0.103 (-0.221 to 0.016), FVC (L) = -0.129 (-0.266 to 0.007) | Some concerns |
| Nihues 2015^32^ | Cross-sectional | Brazil | 121 | Not stated | 36 | Bronchitis, emphysema, pleural effusion | 15.7 | 1-12 years after EOT | Symptoms | uOR 4.09 (1.37 to 12.25), aOR 5.42 (1.69 - 17.34) | High |
| Wu 2022^48^ | Cross-sectional | China | 975 | Not stated | 14 | Has lung disease, including COPD | 5.8 | ≤ 5 years after EOT | CAT | uOR 6.57 (2.86 to 15.07 | Very high |
| **Hypertension Papers** | | | | | | | | | | |  |
| Allwood 2023^10^ | Cross-sectional | South Africa | 100 | Not stated | 15 | - | 2.0 | EOT | Echocardiography | 2 patients with hypertension, neither had pulmonary hypertension. | Very high |
| Louw 2023^22^ | Cross-sectional | South Africa | 100 | Not stated | 72 | - | 21.0 | ≤ 5 years after EOT | Echocardiography | uOR 1.08 (0.21 to 5.69) | Some concerns |
| Mancuzo 2020^23^ | Cross-sectional | Brazil | 378 | Not stated | 19 | - | 24.3 | ≤ 5 years after EOT | Spirometry | uOR 1.70 (0.62 to 4.64) | High |
| Pydipalli 2022^38^ | Cross-sectional | India | 118 | DS only | Ever: 61 | - | 25.4 | ≤ 1 year after EOT | Spirometry | uOR 1.90 (0.76 to 4.75) | Very high |
| Tandon 2021^45^ | Retrospective cohort | India | 60 | DS only | 40 | - | 13.3 | EOT | CXR | uOR 0.33 (0.06 to 1.71) | Very high |
| **Anaemia and Haemoglobin Papers** | | | | | | | | | | |  |
|  |  |  |  |  |  |  |  |  |  |  |  |
| ANAEMIA | | | | | | | | | | | |
| Tandon 2021^45^ | Retrospective cohort | India | 60 | DS only | 40 | Hb <11mg/dL in women or <12mg/dL in men at start of TB treatment | 85 | EOT | CXR | uOR for anaemia vs no-anaemia 2.09 (0.48 to 9.12) | Very high |
| CONTINUOUS HAEMOGLOBIN | | | | | | | | | | | |
| Meghji 2020^26^ | Prospective cohort | Malawi | 368 | DS only | Ever: 29.6 | Hb at EOT as a continuous variable | Not stated | ≤ 1 year after EOT | Symptoms | Chronic respiratory symptoms at 1-year: uOR 0.88 (0.78 to 0.995) Acute respiratory event within 1-year: uOR 0.90 (0.78 to 1.04) | Some concerns |
| Khosa 2020^18^ | Prospective cohort | Mozambique | 62 | All | Ever: 35.5 | Hb <12mg/dL in women or <13mg/dL in men at start of TB treatment | 83.9 | ≤ 1 year after EOT | Spirometry | RRR for each 1g/dl increase in Hb: mild lung impairment (LI) 0.67 (0.47 to 0.95), moderate/severe LI 0.69 (0.49 to 0.96) | Very high |
| **Chronic Kidney Disease (CKD) Papers** | | | | | | | | | | |  |
| Mancuzo 2020^23^ | Cross-sectional | Brazil | 378 | Not stated | 18.8 | - | 5.6 | ≤ 5 years after EOT | Spirometry | No CKD patients had PTLD | High |
| Tandon 2021^45^ | Retrospective cohort | India | 60 | DS only | 40.0 | - | 5.0 | EOT | CXR | uOR 1.73 (0.16 to 18.92) | Very high |
| **Malignancy Papers** | | | | | | | | | | |  |
| Mancuzo 2020^23^ | Cross-sectional | Brazil | 378 | Not stated | 18.8 | - | 2.7 | ≤ 5 years after EOT | Spirometry | uOR 3.69 (0.32 to 42.23) | High |
| Tandon 2021^45^ | Retrospective cohort | India | 60 | DS only | 40.0 | - | 1.7 | EOT | CXR | uOR 0.32 (0.003 to 30.664) | Very high |
| **Other Condition Papers** | | | | | | | | | | |  |
| Chin 2019^14^ | Prospective cohort | Zimbabwe | 175 | Any | 5.7 | Major depression, PTSD | Major depression 13.1%, PTSD 4% | Variable | Composite | Not associated with PTLD | High |
| Mancuzo 2020^23^ | Cross-sectional | Brazil | 378 | Not stated | 18.8 | Cardiac disease | 4.2 | ≤ 5 years after EOT | Spirometry | No PTLD in cardiac disease group | High |
| Namusobya 2023^30^ | Cross-sectional | Uganda | 326 | All | Ever smokers: 20.2 | Immunosuppressive condition other than HIV^e^ | 8 | ≤ 5 years after EOT | Symptoms | aOR 7.72 (3.13 to 19.04) | High |
| Vashakidze 2019^46^ | Cross-sectional | Georgia | 58 | MDR or XDR only | 40 | Hepatitis C | 7 | ≤ 5 years after EOT | Spirometry  SGRQ | Linear regression coefficients: FEV1% -24.49, p-value >0.05, FVC% pred -10.03, p-value >0.05, FEV1/FVC -16.14, p-value 0.01-0.05 SGRQ score 7.17, p-value >0.05. P-values are reporting style of paper. | High |
| Wu 2022^48^ | Cross-sectional | China | 975 | Not stated | 13.7 | Chronic metabolic disorder | 25.23 | ≤ 5 years after EOT | CAT | uOR 1.33 (0.91 to 1.95) | Very high |

^a^ Cannabis n=39, waterpipe n=12, crystal meth n=5, methaquione n=8, other n=4. ^b^ Cannabis 22%. ^c^ Asthma n=14, COPD and post-TB bronchiectasis n=1, other conditions n=4. ^d^ Asthma, COPD, bronchiectasis, interstitial lung disease or silicosis. ^e^ Predominantly diabetes or chronic steroid use. 6MWT = six-minute walk test, aOR = adjusted odds ratio, CAT = COPD assessment tool, CKD = chronic kidney disease, CLD = chronic lung disease, CXR = chest X-ray, DS = drug sensitive, COPD = chronic obstructive pulmonary disease, EOT = end of treatment, FEV1= forced expiratory volume in 1 second, FVC = forced vital capacity, Hb = haemaglobin, HRCT = high-resolution CT, LI = lung impairment, MDR = multi-drug resistant, mMRC = modified medical research council dyspnoea scale, RRR = relative risk ratio, SGRQ = St. George's Respiratory Questionnaire, PH = pulmonary hypertension, PTSD = post-traumatic stress disorder, PTLD = post-TB lung disease, uOR = unadjusted odds ratio, XDR = extensively-drug resistant. Unless stated otherwise, number in brackets after odds ratios are 95% confidence intervals. Stated study sample size is number of participants included in the analysis.

## **Supplementary Figure S2: Sensitivity analysis for meta-analysis of abnormal spirometry by HIV status in adults**

#### Unadjusted odds ratios only


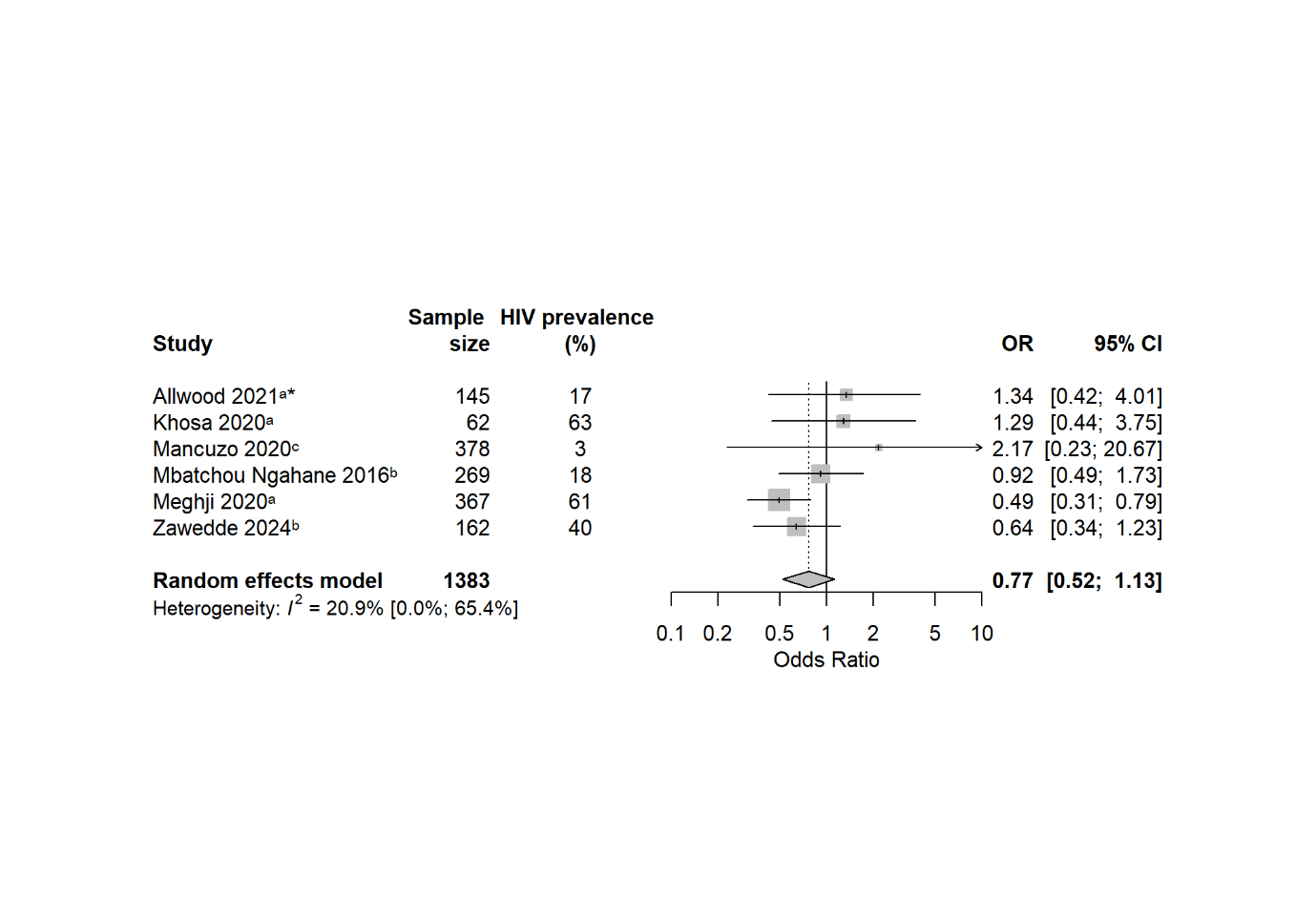


#### Fully adjusted odds ratios only


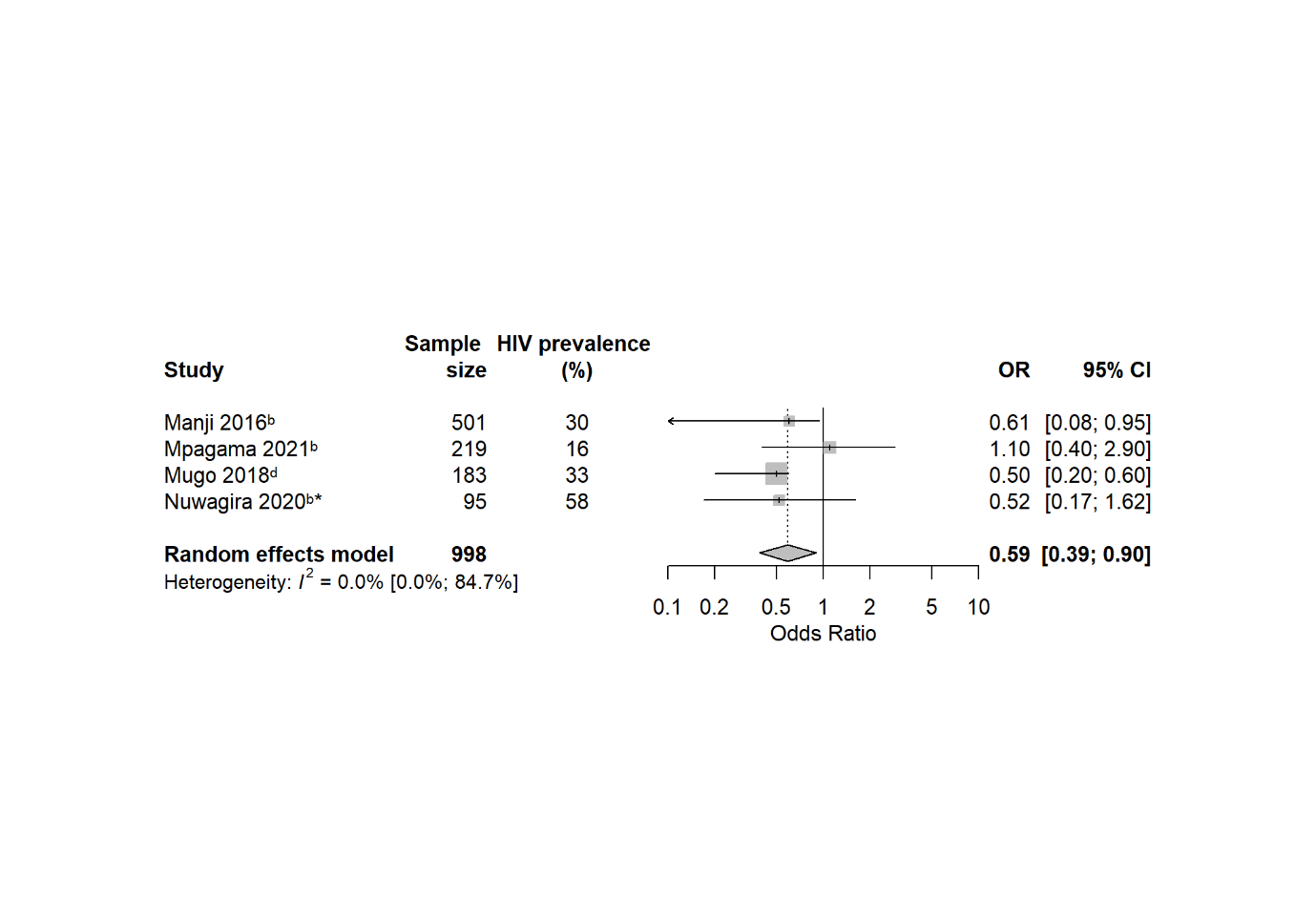


Note: CI = confidence interval. I^2^ reported as value [95% CI]. Study sample size is number of participants included in the co-morbidity – PTLD assessment. ^a^ Airway obstruction = FEV1/FVC <lower limit of normal (LLN) or Z-score <-1.64, restrictive defect = FVC <lower limit of normal or Z-score <-1.64. ^b^ Airway obstruction = FEV1/FVC <0.70, restrictive defect = FVC <0.80 predicted. ^c^ Moderate/severe airway obstruction = FEV1/FVC <0.60, moderate/severe restrictive defect = FVC <0.60 predicted. * Only airway obstruction reported.

## **Supplementary Figure S3: Publication bias for HIV meta-analysis**


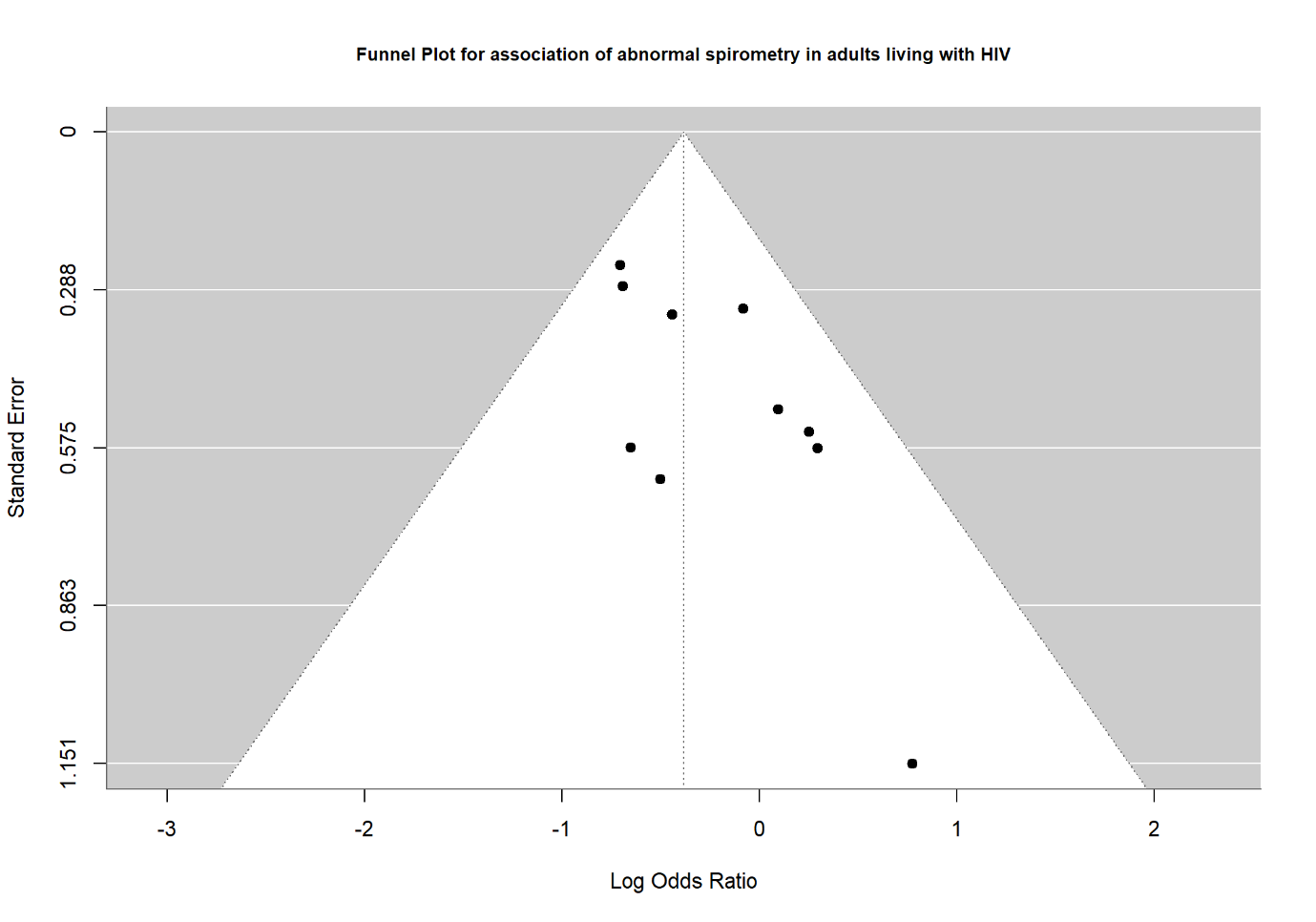


## **Supplementary Figure S4: Publication bias for diabetes meta-analysis**


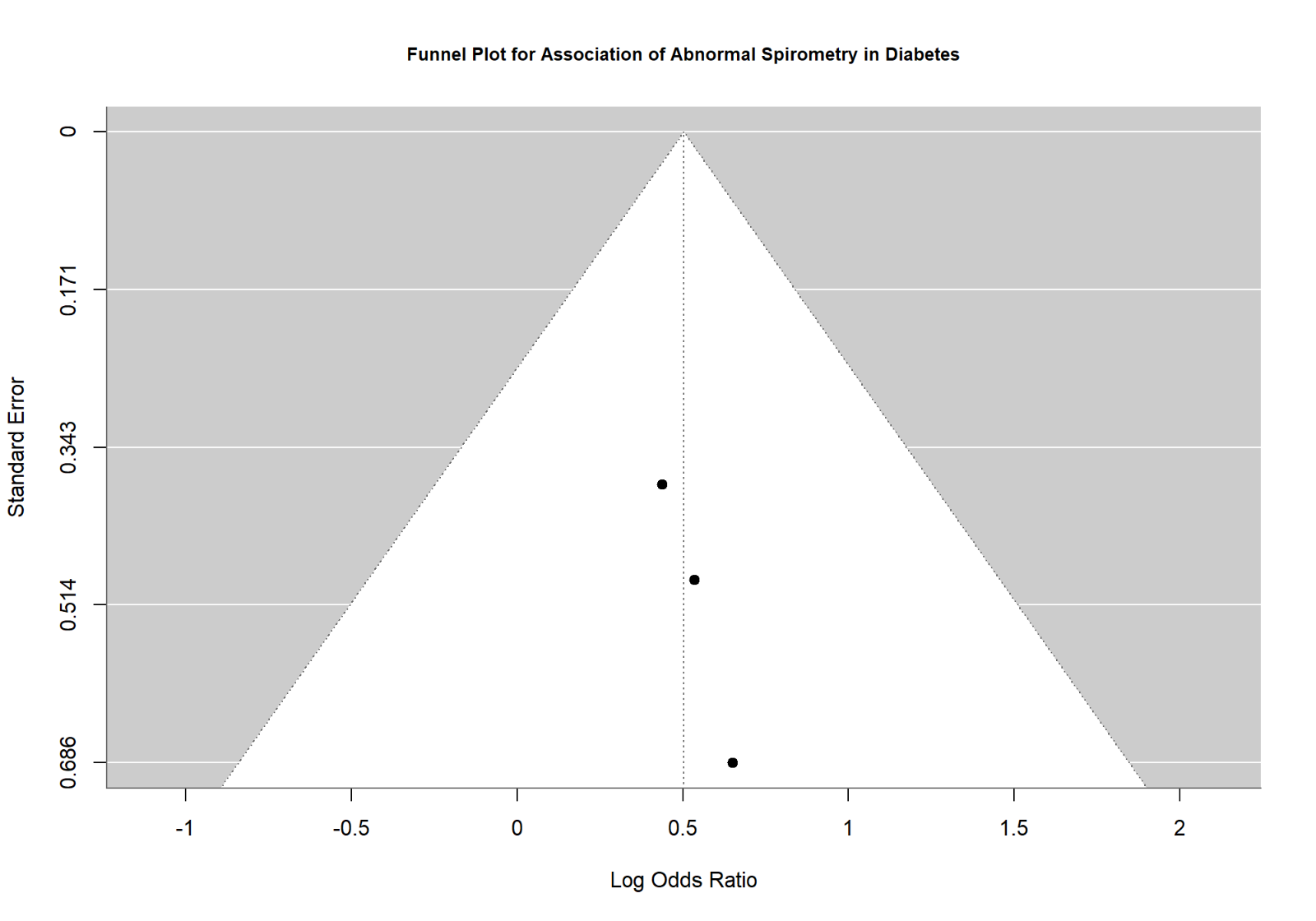


Interpret with caution as fewer than 10 studies included in meta-analysis^51^.

## **Supplementary Figure S5: Diabetes forest plots – contrasting airway obstruction and restrictive spirometry pattern from Gupte 2019^16^**

#### Gupte 2019^16^ included as airway obstruction


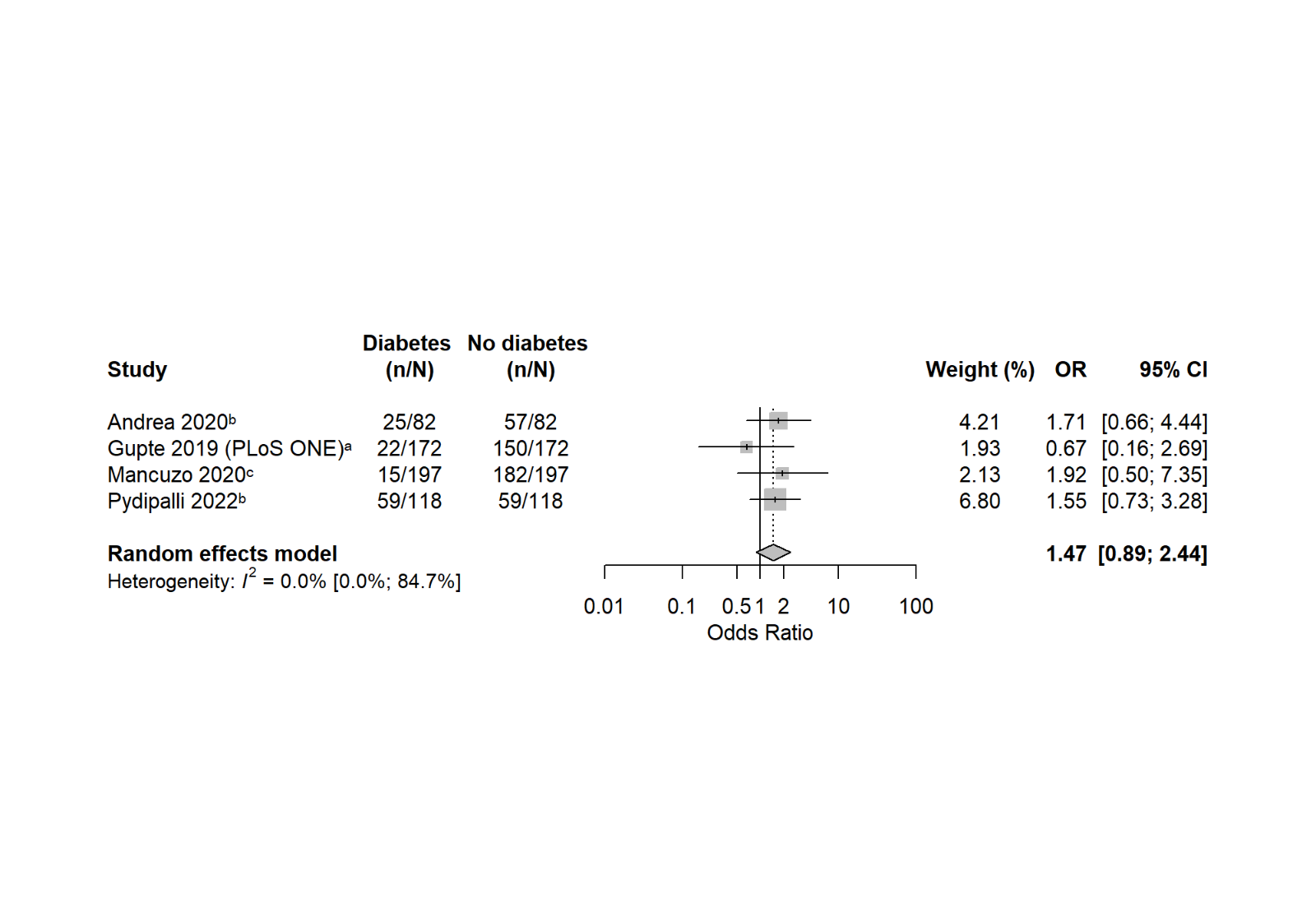


#### Gupte 2019^16^ included as restrictive spirometry pattern


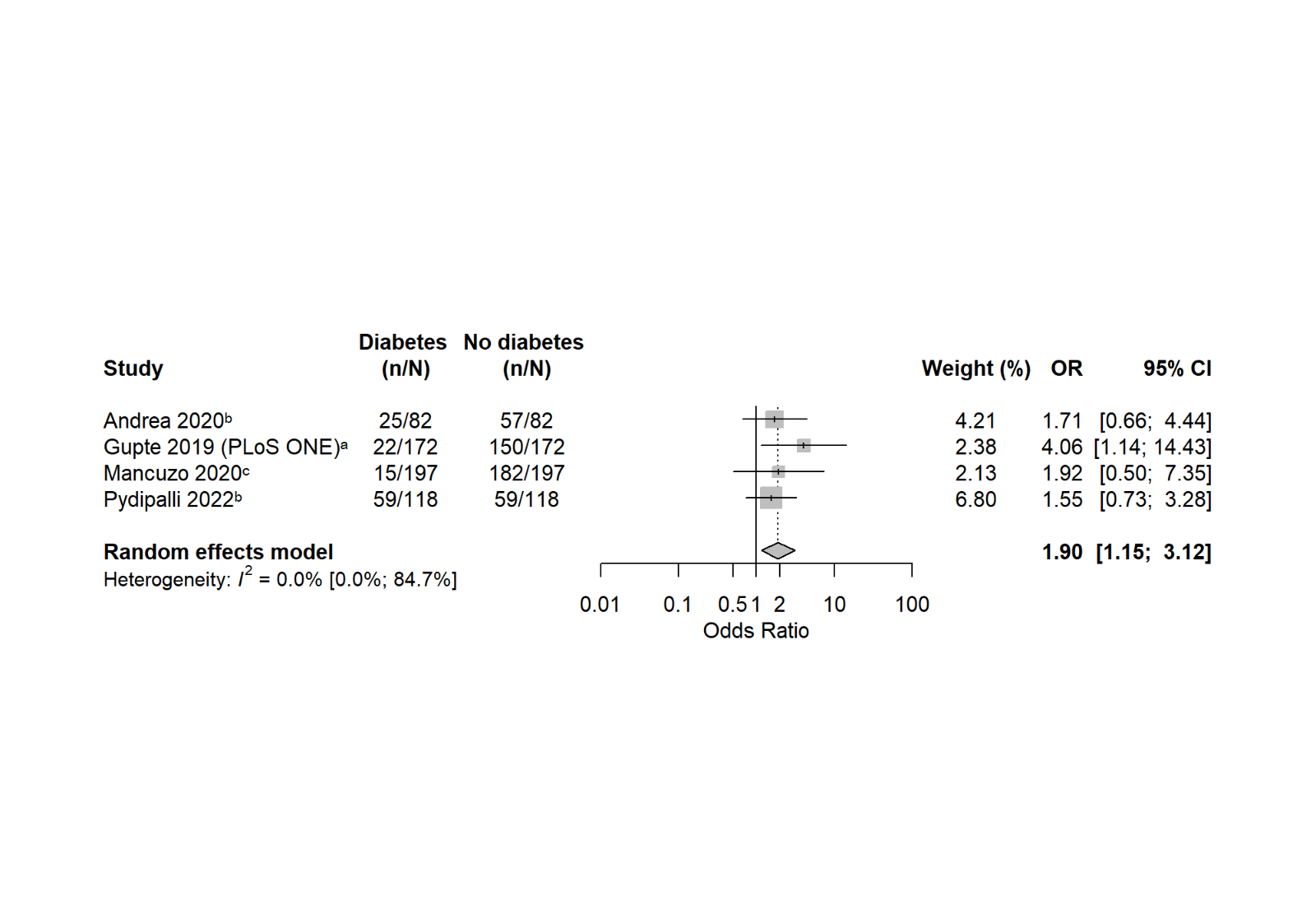


Note: CI = confidence interval. I2 reported as value [95% CI]. Study sample size is number of participants included in the co-morbidity – PTLD assessment. All studies other than Gupte 2019 report odds ratios for any abnormal lung function (airway obstruction and/or restrictive pattern combined). ^a^ Airway obstruction = FEV1/FVC <lower limit of normal (LLN) or Z-score <-1.64, restrictive defect = FVC <lower limit of normal or Z-score <-1.64. ^b^ Airway obstruction = FEV1/FVC <0.70, restrictive defect = FVC <0.80 predicted. ^c^ Moderate/severe airway obstruction = FEV1/FVC <0.60, moderate/severe restrictive defect = FVC <0.60 predicted.

## **Supplementary Figure S6: Publication bias for undernutrition meta-analysis**


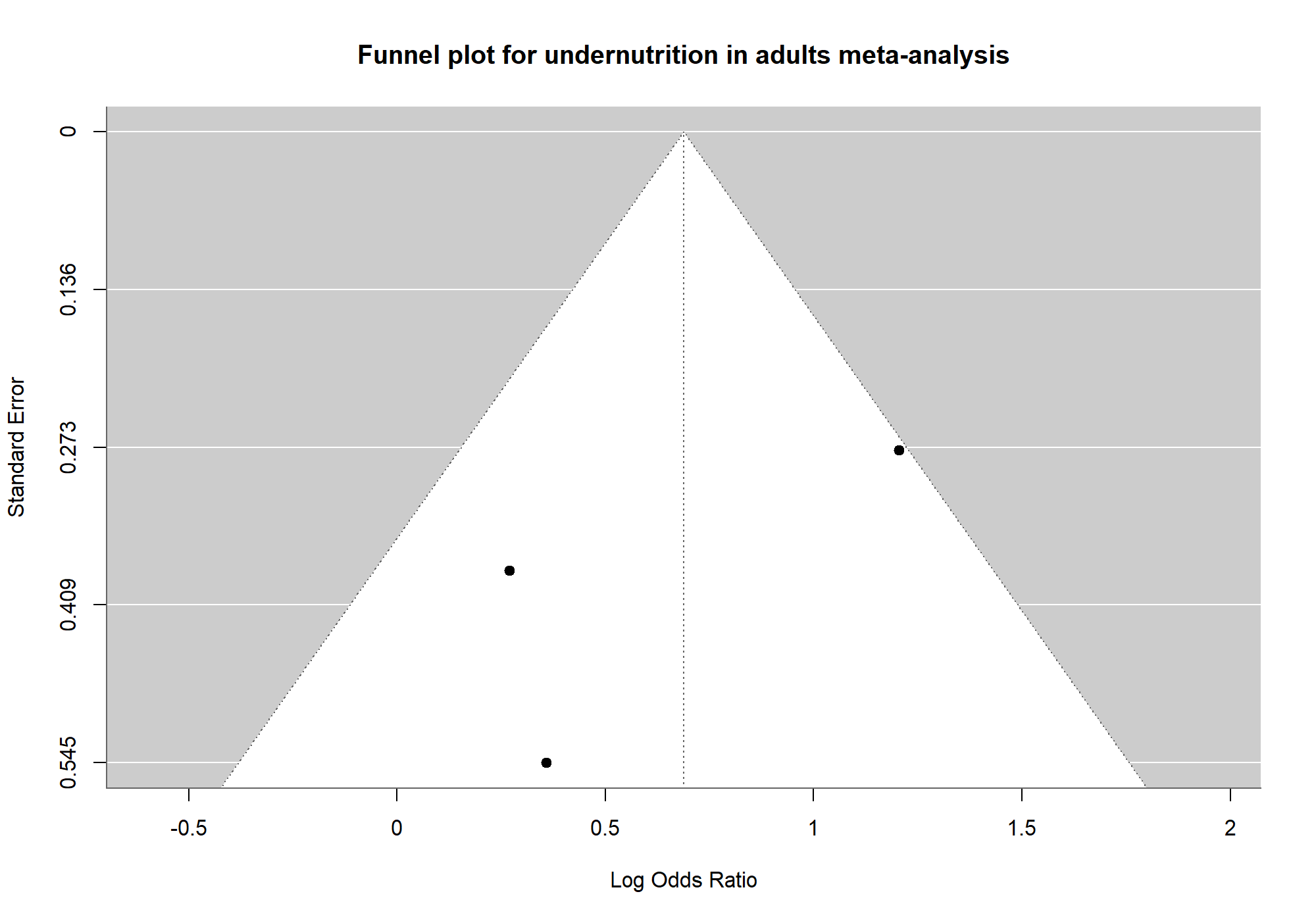


Interpret with caution as fewer than 10 studies included in meta-analysis^51^.

###

## **Supplementary Table S13: GRADE Assessment Results**

The certainty of evidence for each meta-analysed association was assessed using the Grading of Recommendations Assessment, Development and Evaluation (GRADE) framework. In all circumstances the outcome is the result of spirometry assessment, categorised into normal or abnormal.

| **Exposure** | **Number of participants (studies)** | **Risk of Bias** | **Inconsistency** | **Indirectness** | **Imprecision** | **Publication Bias** | **Large effect** | **Dose-response gradient** | **Residual confounding** | **Certainty of evidence (GRADE)** |
| --- | --- | --- | --- | --- | --- | --- | --- | --- | --- | --- |
| HIV | 2153 (10) | Very serious limitation | Serious limitation | No serious limitation | No serious limitation | Serious limitation | - | N/A* | - | ⊕◯◯◯  Very low certainty |
| Diabetes mellitus | 397 (3) | Very serious limitation | Serious limitation | No serious limitation | Serious limitation | N/A** | - | N/A* | - | ⊕◯◯◯  Very low certainty |
| Undernutrition | 391 (3) | Very serious limitation | Very serious limitation | No serious limitation | No serious limitation | N/A** | - | N/A | - | ⊕◯◯◯  Very low certainty |

*=Not applicable as all studies categorised the exposure as a binary variable. **=Unable to assess publication bias due to fewer than 10 studies included.

## **Supplementary Table S14: Papers excluded at full text stage with brief reasons**

| **Paper** | **Exclusion Reason** |
| --- | --- |
| Abdelaleem 2022 | Morbidities other than TB excluded |
| Abelman 2023 | No co-morbidity analysis |
| Agarwal 2014 | Wrong study design |
| Aggarwal 2023 | No morbidity other than TB and PTLD described |
| Agizew 2020 | No assessment of respiratory system/PTLD |
| Ahmed 2022 | Morbidities other than TB excluded |
| Ahmed 2022 | Morbidities other than TB excluded |
| Aishwarya 2022 | No morbidity other than TB and PTLD described |
| Akkara 2013 | No morbidity other than TB and PTLD described |
| Alami 2015 | Wrong population group |
| Ali 2018 | Poster/oral presentation without enough information to analyse |
| Allwood 2019 | Poster/oral presentation without enough information to analyse |
| Allwood 2020 | No morbidity other than TB and PTLD described |
| Amosov 1985 | Wrong study design |
| Anastasatu 1971 | Review |
| Auld 2020 | Poster/oral presentation without enough information to analyse |
| Ayari 2015 | Wrong population group |
| Bansal 2023 | Review |
| BanuRekha 2009 | No co-morbidity analysis |
| Bastos 2014 | No assessment of PTLD |
| BenSassi 2022 | No comparator group |
| Bhatta 2008 | Wrong population group |
| Binegdie 2018 | Poster/oral presentation without enough information to analyse |
| Binegdie 2022 | Wrong population group |
| Binegdie 2015 | Wrong population group |
| Bombarda 2003 | No morbidity other than TB and PTLD described |
| Braude 1980 | Wrong study design |
| Busila-Corabianu 1970 | Wrong study design |
| Byrne 2017 | No morbidity other than TB and PTLD described |
| Capone 2017 | Morbidities other than TB excluded |
| Chakravarti 2023 | No morbidity other than TB and PTLD described |
| Chen 2021 | No assessment of PTLD |
| Chen 2021 | No assessment of PTLD |
| Chong 2019 | Duplicate of already included study |
| Christine 2019 | No morbidity other than TB and PTLD described |
| Christopher 2020 | No morbidity other than TB and PTLD described |
| Chung 2010 | Wrong setting (HIC country/region only) |
| Chung 2011 | Wrong setting (HIC country/region only) |
| Chushkin 2012 | No morbidity other than TB and PTLD described |
| Chushkin 2011 | Poster/oral presentation without enough information to analyse |
| Chushkin 2013 | No morbidity other than TB and PTLD described |
| Chushkin 2012 | No morbidity other than TB and PTLD described |
| Chushkin 2012 | No morbidity other than TB and PTLD described |
| Chushkin 2013 | No morbidity other than TB and PTLD described |
| Chushkin 2011 | No morbidity other than TB and PTLD described |
| Chushkin 2012 | No morbidity other than TB and PTLD described |
| Chushkin 2011 | No morbidity other than TB and PTLD described |
| Chushkin 2011 | Poster/oral presentation without enough information to analyse |
| Chushkin 2013 | No morbidity other than TB and PTLD described |
| Chushkin 2017 | No co-morbidity analysis |
| Cole 2016 | No morbidity other than TB and PTLD described |
| Costantini 1961 | Review |
| Cupido 2024 | Review |
| Daidano 2017 | Wrong population group |
| Damian 2018 | Poster/oral presentation without enough information to analyse |
| Daniels 2020 | No morbidity other than TB and PTLD described |
| Daniels 2019 | No co-morbidity analysis |
| Davies 2022 | Wrong population group |
| Ddungu 2022 | No co-morbidity analysis |
| Ddungu 2021 | No co-morbidity analysis |
| DeLaMora 2015 | No morbidity other than TB and PTLD described |
| DeRosa 1998 | Review |
| deVallière 2004 | No co-morbidity analysis |
| Denning 2010 | Wrong study design |
| Dewi 2023 | No morbidity other than TB and PTLD described |
| Dhar 2019 | Wrong population group |
| DiNaso 2011 | No co-morbidity analysis |
| Dias 2022 | Wrong population group |
| Didilescu 1981 | Can't find full text |
| Didilescu 1981 | No assessment of PTLD |
| Du 2023 | No morbidity other than TB and PTLD described |
| Ekawati 2017 | No morbidity other than TB and PTLD described |
| Evfim'evskii 1999 | Can't find full text |
| Ezzaouia 2019 | Poster/oral presentation without enough information to analyse |
| Fang 2024 | Morbidities other than TB excluded |
| Fawibe 2012 | Wrong population group |
| Ferchichi 2021 | No co-morbidity analysis |
| Fitzpatrick 2023 | No co-morbidity analysis |
| Freinate 1988 | Wrong population group |
| Ganmaa 2016 | No assessment of PTLD |
| Gharsalli 2013 | No morbidity other than TB and PTLD described |
| Gie 1997 | Poster/oral presentation without enough information to analyse |
| Gimeno 1977 | No morbidity other than TB and PTLD described |
| Gomez-Olivas 2023 | Review |
| Graczyk 1971 | No morbidity other than TB and PTLD described |
| Guliani 2014 | No morbidity other than TB and PTLD described |
| Gupta 2018 | No morbidity other than TB and PTLD described |
| Gupta 2022 | No morbidity other than TB and PTLD described |
| Gupta 2022 | Morbidities other than TB excluded |
| Heo 2009 | Wrong setting (HIC country/region only) |
| Hill 1959 | Wrong population group |
| Ho 2021 | Wrong setting (HIC country/region only) |
| Houda 2016 | No assessment of PTLD |
| Hsu 2020 | Wrong study design |
| Huang 1977 | Wrong population group |
| Hutchison 1951 | Morbidities other than TB excluded |
| Irfan 2016 | Review |
| Jagmohan 2022 | No comparator group |
| Jain 2021 | No morbidity other than TB and PTLD described |
| Jaligidad 2016 | No morbidity other than TB and PTLD described |
| Jeune 1951 | Wrong study design |
| Jian 2016 | Wrong setting (HIC country/region only) |
| Jimenez-Corona 2013 | No assessment of PTLD |
| Jithoo 2011 | Wrong population group |
| Jones 2021 | Wrong setting (HIC country/region only) |
| Joo 2023 | Wrong setting (HIC country/region only) |
| Kalandadze 1984 | No morbidity other than TB and PTLD described |
| Kalua 2018 | Wrong population group |
| Katoto 2022 | No co-morbidity analysis |
| Kavya 2020 | Wrong population group |
| Khaled 2018 | No co-morbidity analysis |
| Khara 2016 | No comparator group |
| Kim 2022 | Wrong setting (HIC country/region only) |
| Kim 2001 | Review |
| Kim 2019 | Wrong setting (HIC country/region only) |
| Kim 2016 | Wrong setting (HIC country/region only) |
| Kishan 2020 | No morbidity other than TB and PTLD described |
| Ko 2015 | Wrong setting (HIC country/region only) |
| Kulshrestha 2022 | No co-morbidity analysis |
| Kumar 2018 | No comparator group |
| Kumar 2023 | Wrong population group |
| Lakhtakia 2022 | Wrong population group |
| Lalwani 2022 | Wrong population group |
| LaniadoDeLaMora 2014 | No morbidity other than TB and PTLD described |
| Latif 2022 | Morbidities other than TB excluded |
| Lawal 2020 | No assessment of PTLD |
| Liang 2009 | Wrong population group |
| Madegedara 2015 | No morbidity other than TB and PTLD described |
| Maenetje 2023 | No co-morbidity analysis |
| Mahishale 2017 | No assessment of PTLD |
| Mahishale 2015 | No assessment of PTLD |
| Maleche-Obimbo 2022 | Review |
| Malherbe 2016 | Morbidities other than TB excluded |
| Marcatili 1976 | Wrong study design |
| Marcos 2011 | Wrong setting (HIC country/region only) |
| Marie 1946 | No assessment of PTLD |
| Martinez 2023 | No morbidity other than TB and PTLD described |
| Mayo 2000 | No morbidity other than TB and PTLD described |
| Meawed 2012 | Wrong population group |
| Mehra 1985 | Review |
| Menon 2015 | No morbidity other than TB and PTLD described |
| Mkoko 2019 | Wrong population group |
| Mp 2022 | Wrong population group |
| Mukasa 2022 | No co-morbidity analysis |
| Muller 2023 | No assessment of PTLD |
| Munoz-Torrico 2020 | No co-morbidity analysis |
| Muzanyi 2019 | No assessment of PTLD |
| Nagu 2017 | No co-morbidity analysis |
| Namusobya 2023 | Wrong population group |
| Nguyen 2021 | Wrong population group |
| Nightingale 2020 | Duplicate of already included study |
| Nima 2013 | No morbidity other than TB and PTLD described |
| Nishi 2021 | No co-morbidity analysis |
| Nishi 2021 | Wrong population group |
| Nsubuga 2002 | No assessment of PTLD |
| Nuwagira 2023 | No co-morbidity analysis |
| Ocansey 2022 | No morbidity other than TB and PTLD described |
| Ocansey 2023 | No co-morbidity analysis |
| Ojuawo 2020 | No co-morbidity analysis |
| Oladele 2022 | Morbidities other than TB excluded |
| Orooj 2023 | Morbidities other than TB excluded |
| Osman 2016 | No co-morbidity analysis |
| Ouedraogo 2019 | No morbidity other than TB and PTLD described |
| Ozoh 2021 | Wrong population group |
| Page 2017 | Duplicate or already included study |
| Palma 2018 | Morbidities other than TB excluded |
| Pasipanodya 2012 | Wrong setting (HIC country/region only) |
| Patel 2015 | Morbidities other than TB excluded |
| Patil 2018 | Morbidities other than TB excluded |
| Poey 1997 | No morbidity other than TB and PTLD described |
| Prakash 2021 | No assessment of PTLD |
| Puvinel 1967 | Wrong study design |
| Racil 2010 | No morbidity other than TB and PTLD described |
| Radovic 2016 | Morbidities other than TB excluded |
| Ramos 2006 | No morbidity other than TB and PTLD described |
| Ranjan 2022 | No assessment of PTLD |
| Ravimohan 2020 | No co-morbidity analysis |
| Ravindranath 2022 | No morbidity other than TB and PTLD described |
| Ray 2023 | Wrong population group |
| Ray 2023 | Wrong population group |
| Rhee 2013 | Wrong setting (HIC country/region only) |
| Robertson 2020 | No co-morbidity analysis |
| Rozaliyani 2020 | Wrong population group |
| Saifullah 2023 | Wrong population group |
| Sailaja 2015 | No morbidity other than TB and PTLD described |
| Samanta 2022 | No comparator group |
| Santra 2017 | Wrong population group |
| Sehgal 2024 | Review |
| Setianingrum 2022 | No co-morbidity analysis |
| Shani 2018 | Morbidities other than TB excluded |
| Shanmugasundaram 2022 | Morbidities other than TB excluded |
| Sharma 2022 | No co-morbidity analysis |
| Sharma 2012 | No morbidity other than TB and PTLD described |
| Sharma 2006 | No assessment of PTLD |
| Shetty 2010 | No morbidity other than TB and PTLD described |
| Silva 2023 | Review |
| Silva 2022 | No co-morbidity analysis |
| Singh 2022 | No morbidity other than TB and PTLD described |
| Singla 2009 | No co-morbidity analysis |
| Singla 2018 | No co-morbidity analysis |
| Singla 2021 | Wrong population group |
| Soni 2016 | Poster/oral presentation without enough information to analyse |
| Stek 2020 | No morbidity other than TB and PTLD described |
| Tambunan 2017 | Poster/oral presentation without enough information to analyse |
| Tarigan 2017 | No morbidity other than TB and PTLD described |
| Tatar 2009 | No assessment of PTLD |
| Thienemann 2022 | Wrong population group |
| Thoker 2023 | Wrong population group |
| Tiewsoh 2020 | No assessment of PTLD |
| Tomeny 2022 | No assessment of PTLD |
| Torres 2021 | Wrong population group |
| Toussaint 1961 | Review |
| Tweed 2022 | Poster/oral presentation without enough information to analyse |
| Ullmann 2017 | Wrong setting (HIC country/region only) |
| vanderZalm 2024 | No morbidity other than TB and PTLD described |
| Vecino 2011 | Wrong setting (HIC country/region only) |
| Villar 1974 | Can't find full text |
| Wallis 2022 | Morbidities other than TB excluded |
| Wang 2010 | Wrong setting (HIC country/region only) |
| Willcox 1989 | No morbidity other than TB and PTLD described |
| Win 2019 | No co-morbidity analysis |
| Wolmarans 2023 | No co-morbidity analysis |
| Wolmarans 2022 | No co-morbidity analysis |
| Xia 2018 | No co-morbidity analysis |
| Xing 2023 | No co-morbidity analysis |
| Yanardag 2003 | Wrong population group |
| Zhang 2024 | Wrong population group |
| Zifodya 2023 | No co-morbidity analysis |
| Zneigui 2019 | Wrong population group |
| Zubair 2021 | No morbidity other than TB and PTLD described |

TB = tuberculosis, PTLD = post-tuberculosis lung disease, HIC = high-income country

## **References used in appendices**

1. Allwood BW, van der Zalm MM, Amaral AFS, et al. Post-tuberculosis lung health: perspectives from the First International Symposium. *Int J Tuberc Lung Dis* 2020; **24**(8): 820–8.

2. Guyatt GH, Oxman AD, Schünemann HJ, Tugwell P, Knottnerus A. GRADE guidelines: A new series of articles in the <em>Journal of Clinical Epidemiology</em>. *Journal of Clinical Epidemiology* 2011; **64**(4): 380–2.

3. Group GW, Schünemann HJ, Cuello C, et al. GRADE guidelines: 18. How ROBINS-I and other tools to assess risk of bias in nonrandomized studies should be used to rate the certainty of a body of evidence. *Journal of Clinical Epidemiology* 2019; **111**: 105–14.

4. Guyatt GH, Oxman AD, Kunz R, et al. GRADE guidelines 6. Rating the quality of evidence&#x2014;imprecision. *Journal of Clinical Epidemiology* 2011; **64**(12): 1283–93.

5. Guyatt GH, Oxman AD, Montori V, et al. GRADE guidelines: 5. Rating the quality of evidence&#x2014;publication bias. *Journal of Clinical Epidemiology* 2011; **64**(12): 1277–82.

6. Guyatt GH, Oxman AD, Vist G, et al. GRADE guidelines: 4. Rating the quality of evidence&#x2014;study limitations (risk of bias). *Journal of Clinical Epidemiology* 2011; **64**(4): 407–15.

7. The GWG, Guyatt GH, Oxman AD, et al. GRADE guidelines: 8. Rating the quality of evidence&#x2014;indirectness. *Journal of Clinical Epidemiology* 2011; **64**(12): 1303–10.

8. The GWG, Guyatt GH, Oxman AD, et al. GRADE guidelines: 7. Rating the quality of evidence&#x2014;inconsistency. *Journal of Clinical Epidemiology* 2011; **64**(12): 1294–302.

9. The GWG, Guyatt GH, Oxman AD, et al. GRADE guidelines: 9. Rating up the quality of evidence. *Journal of Clinical Epidemiology* 2011; **64**(12): 1311–6.

10. Allwood BW, Manie S, Stolbrink M, et al. Pulmonary hypertension in adults completing tuberculosis treatment. *Afr J Thorac Crit Care Med* 2023; **29**(3).

11. Allwood BW, Stolbrink M, Baines N, et al. Persistent chronic respiratory symptoms despite TB cure is poorly correlated with lung function. *Int J Tuberc Lung Dis* 2021; **25**(4): 262–70.

12. Andrea BYL, Faisal AH, Chong GY, Syed ZSZ. Incidence of post-tuberculosis chronic obstructive pulmonary disease in a tertiary centre in Malaysia. *Med & Health Dec* 2020; **15**(2): 85–95.

13. Auld SC, Kornfeld H, Maenetje P, et al. Pulmonary restriction predicts long-term pulmonary impairment in people with HIV and tuberculosis. *BMC Pulm Med* 2021; **21**(1): 19.

14. Chin AT, Rylance J, Makumbirofa S, et al. Chronic lung disease in adult recurrent tuberculosis survivors in Zimbabwe: a cohort study. *Int J Tuberc Lung Dis* 2019; **23**(2): 203–11.

15. Gandhi K, Gupta S, Singla R. Risk factors associated with development of pulmonary impairment after tuberculosis. *Indian J Tuberc* 2016; **63**(1): 34–8.

16. Gupte AN, Paradkar M, Selvaraju S, et al. Assessment of lung function in successfully treated tuberculosis reveals high burden of ventilatory defects and COPD. *PLoS One* 2019; **14**(5): e0217289.

17. Gupte AN, Selvaraju S, Paradkar M, et al. Respiratory health status is associated with treatment outcomes in pulmonary tuberculosis. *Int J Tuberc Lung Dis* 2019; **23**(4): 450–7.

18. Khosa C, Bhatt N, Massango I, et al. Development of chronic lung impairment in Mozambican TB patients and associated risks. *BMC Pulm Med* 2020; **20**(1): 127.

19. Kumar Rai D, Kumar R. Identification of risk factors for radiological sequelae in patients treated for pulmonary tuberculosis: Prospective observational cohort study. *Indian J Tuberc* 2020; **67**(4): 534–8.

20. Lin Y, Liu Y, Zhang G, et al. Is It Feasible to Conduct Post-Tuberculosis Assessments at the End of Tuberculosis Treatment under Routine Programmatic Conditions in China? *Trop Med Infect Dis* 2021; **6**(3).

21. Lisha PV, James PT, Ravindran C. Morbidity and mortality at five years after initiating category I treatment among patients with new sputum smear positive pulmonary tuberculosis. *Indian Journal of Tuberculosis* 2012; **59**: 83–91.

22. Louw E, Baines N, Maarman G, et al. The prevalence of pulmonary hypertension after successful tuberculosis treatment in a community sample of adult patients. *Pulm Circ* 2023; **13**(1): e12184.

23. Mancuzo EV, Martins Netto E, Sulmonett N, et al. Spirometry results after treatment for pulmonary tuberculosis: comparison between patients with and without previous lung disease: a multicenter study. *J Bras Pneumol* 2020; **46**(2): e20180198.

24. Manji M, Shayo G, Mamuya S, Mpembeni R, Jusabani A, Mugusi F. Lung functions among patients with pulmonary tuberculosis in Dar es Salaam - a cross-sectional study. *BMC Pulm Med* 2016; **16**(1): 58.

25. Mbatchou Ngahane BH, Nouyep J, Nganda Motto M, et al. Post-tuberculous lung function impairment in a tuberculosis reference clinic in Cameroon. *Respir Med* 2016; **114**: 67–71.

26. Meghji J, Lesosky M, Joekes E, et al. Patient outcomes associated with post-tuberculosis lung damage in Malawi: a prospective cohort study. *Thorax* 2020; **75**(3): 269–78.

27. Mily A, Sarker P, Taznin I, et al. Slow radiological improvement and persistent low-grade inflammation after chemotherapy in tuberculosis patients with type 2 diabetes. *BMC Infect Dis* 2020; **20**(1): 933.

28. Mpagama SG, Msaji KS, Kaswaga O, et al. The burden and determinants of post-TB lung disease. *Int J Tuberc Lung Dis* 2021; **25**(10): 846–53.

29. Mugo PN. Pulmonary function and quality of life in patients with treated smear positive pulmonary tuberculosis at Riruta, Kangemi and Kibera tuberculosis clinics in Nairobi. Univeristy of Nairobi Digital Repository: University of Nairobi; 2013.

30. Namusobya M, Bongomin F, John M, et al. Chronic respiratory symptoms and chronic obstructive pulmonary disease following completion of pulmonary tuberculosis treatment in Uganda. *medRxiv* 2023.

31. Nightingale R, Chinoko B, Lesosky M, et al. Respiratory symptoms and lung function in patients treated for pulmonary tuberculosis in Malawi: a prospective cohort study. *Thorax* 2022; **77**(11): 1131–9.

32. Nihues Sde S, Mancuzo EV, Sulmonetti N, et al. Chronic symptoms and pulmonary dysfunction in post-tuberculosis Brazilian patients. *Braz J Infect Dis* 2015; **19**(5): 492–7.

33. Nkereuwem E, Agbla S, Njai B, et al. Post-tuberculosis respiratory impairment in Gambian children and adolescents: A cross-sectional analysis. *Pediatr Pulmonol* 2024; **59**(7): 1912–21.

34. Nuwagira E, Stadelman A, Baluku JB, et al. Obstructive lung disease and quality of life after cure of multi-drug-resistant tuberculosis in Uganda: a cross-sectional study. *Trop Med Health* 2020; **48**: 34.

35. Osman M, Welte A, Dunbar R, et al. Morbidity and mortality up to 5 years post tuberculosis treatment in South Africa: A pilot study. *Int J Infect Dis* 2019; **85**: 57–63.

36. Page ID, Byanyima R, Hosmane S, et al. Chronic pulmonary aspergillosis commonly complicates treated pulmonary tuberculosis with residual cavitation. *Eur Respir J* 2019; **53**(3).

37. Pefura-Yone EW, Kengne AP, Tagne-Kamdem PE, Afane-Ze E. Clinical significance of low forced expiratory flow between 25% and 75% of vital capacity following treated pulmonary tuberculosis: a cross-sectional study. *BMJ Open* 2014; **4**(7): e005361.

38. Pydipalli M, Chinnakali P, Rajaram M, Sundaram SP, Roy G. Lung Function Impairment In Patients Treated For Pulmonary Tuberculosis and Associated Factors in Puducherry, South India. *Indian J Community Med* 2022; **47**(1): 111–5.

39. Ralph AP, Kenangalem E, Waramori G, et al. High morbidity during treatment and residual pulmonary disability in pulmonary tuberculosis: under-recognised phenomena. *PLoS One* 2013; **8**(11): e80302.

40. Ross J, Ehrlich RI, Hnizdo E, White N, Churchyard GJ. Excess lung function decline in gold miners following pulmonary tuberculosis. *Thorax* 2010; **65**(11): 1010–5.

41. Salzer HJF, Massango I, Bhatt N, et al. Seroprevalence of Aspergillus-Specific IgG Antibody among Mozambican Tuberculosis Patients. *J Fungi (Basel)* 2021; **7**(8).

42. Santamaria-Alza Y, Romero RSK, Sanchez RKJ, et al. Factors associated with the presence of thoracic fibrocavitary sequelae in patients with a history of pulmonary tuberculosis from Hospital Universitario de Santander, Bucaramanga, Colombia. *Revista Americana de Medicina Respiratoria* 2017; **2**: 142–7.

43. Soemarwoto RAS MA, Rusmini H, Arlek M. Factors affecting the occurrence of tuberculosis destroyed lung. *Indian J For Med Toxicol* 2021; **15**(1): 1432–7.

44. Swaminathan S, Narendran G, Menon PA, et al. Impact of HIV infection on radiographic features in patients with pulmonary tuberculosis. *The Indian journal of chest diseases & allied sciences* 2007.

45. Tandon R, Agarwal A, Nirala P, Sinha A. Evaluation of radiological sequelae after treatment completion in new cases of pulmonary and pleural tuberculosis. *Int J Mycobacteriol* 2021; **10**(4): 398–404.

46. Vashakidze SA, Kempker JA, Jakobia NA, et al. Pulmonary function and respiratory health after successful treatment of drug-resistant tuberculosis. *Int J Infect Dis* 2019; **82**: 66–72.

47. Wu H, Asad UK, Wu J, Zhang G, Zhang G, Lu X. CT findings of TB in diabetic and non-diabetic patients: A comparison before and after anti-tuberculous therapy. *Radiology of Infectious Diseases* 2016; **3**: 15–22.

48. Wu Q, Jiang J, Zhang Y, et al. Evaluation of quality of life in 975 patients with pulmonary tuberculosis within 3 years after treatment in Shanghai. *Shanghai Journal of Preventive Medicine* 2022; **34**(2): 163–7.

49. Zawedde J, Abelman R, Musisi E, et al. Lung function and health-related quality of life among adult patients following pulmonary TB treatment. *Int J Tuberc Lung Dis* 2024; **28**(9): 419–26.

50. Zubair SM, Ali MG, Irfan M. Post tuberculosis radiological sequelae in patients treated for pulmonary and pleural tuberculosis at a tertiary center in Pakistan. *Monaldi Arch Chest Dis* 2021; **92**(1).

51. Sterne JAC, Sutton AJ, Ioannidis JPA, et al. Recommendations for examining and interpreting funnel plot asymmetry in meta-analyses of randomised controlled trials. *BMJ* 2011; **343**: d4002.
