# Supplementary material for: Multimorbidity and the risk of post-tuberculosis lung disease: a systematic review and meta-analysis
Source: BMJ Glob Health. 2026 Jun 1;11(6):e020365. doi: 10.1136/bmjgh-2025-020365 (PMC13239530; doi:10.1136/bmjgh-2025-020365)
Supplement: online supplemental file 2 [file bmjgh-11-6-s002.docx]

### BMJ Global Health Author Reflexivity Statement

Adapted from Morton, B., Vercueil, A., Masekela, R., Heinz, E., Reimer, L., Saleh, S., Kalinga, C., Seekles, M., Biccard, B., Chakaya, J., Abimbola, S., Obasi, A. and Oriyo, N. (2022), Consensus statement on measures to promote equitable authorship in the publication of research from international partnerships. Anaesthesia, 77: 264-276. <https://doi.org/10.1111/anae.15597>

| **Study conceptualisation** | |
| --- | --- |
| 1. How does this study address local research and policy priorities? | There is no “local” in this systematic review and meta-analysis. The search was restricted to low- and middle-income countries (LMICs) to reflect the epidemiology and management of LTCs, and the socioeconomic determinants of chronic lung disease, in countries which account for 99% of people newly diagnosed with TB each year. |
| 1. How were local researchers involved in study design? | N/A – there is no “local” in this systematic review and meta-analysis. |
| **Research management** | |
| 1. How has funding been used to support the local research team(s)? | N/A – there is no “local” in this research team. |
| **Data acquisition and analysis** | |
| 1. How are research staff who conducted data collection acknowledged? | N/A – no new data was collected |
| 1. How have members of the research partnership been provided with access to study data? | N/A – all data used in this systematic review and meta-analysis is already published and available. |
| 1. How were data used to develop analytical skills within the partnership? | N/A – there was no new data generated in this study. The narrative synthesis and meta-analysis of the data from published literature was conducted by KJH under the supervision of her PhD supervisors – CS, DJS, HRS, SEM and SM. |
| **Data interpretation** | |
| 1. How have research partners collaborated in interpreting study data? | All authors of this systematic review and meta-analysis contributed to the final version of the manuscript, which included interpretation of study data. |
| **Drafting and revising for intellectual content** | |
| 1. How were research partners supported to develop writing skills? | KJH wrote the first draft. All authors contributed to the final draft. Supervision was provided throughout by CS, DJS, HRS, SEM and SM, two of whom are researchers based in LMICs. |
| 1. How will research products be shared to address local needs? | There is no “local” in this systematic review. This article will be published open access to ensure that stakeholders from LMICs and HICs have equal access to the outputs. |
| **Authorship** | |
| 1. How is the leadership, contribution and ownership of this work by LMIC researchers recognised within the authorship? | IM (from the Infectious Diseases Institute (IDI), Uganda) was a second reviewer. CS (from IDI) and SM (from Kibong’oto Infectious Diseases Institute, Tanzania) were supervisors. KJH is a doctoral training fellow from the UK (the University of St Andrews) and a voluntary member of staff at IDI. She conceived of the study, developed the methodology, curated the data, conducted the analysis and wrote the first draft of the manuscript.  Author order reflects the relative contributions of all team members. |
| 1. How have early career researchers across the partnership been included within the authorship team? | KJH, MS, BW and IM are all early career researchers and took prominent roles in this work. |
| 1. How has gender balance been addressed within the authorship? | Gender balance was considered in the composition of the author team, with women comprising the majority of authors (6/9), including in leading roles. Authorship reflects substantive contributions to the work, with equitable inclusion and recognition irrespective of gender. |
| **Training** | |
| 1. How has the project contributed to training of LMIC researchers? | Through this systematic review IM and KJH, who are both early career researchers based in an LMIC, gained in-depth experience in systematic review methodology and academic writing. |
| **Infrastructure** | |
| 1. How has the project contributed to improvements in local infrastructure? | N/A – there is no “local” in this work. |
| **Governance** | |
| 1. What safeguarding procedures were used to protect local study participants and researchers? | N/A – there is no “local” in this work and there were no study participants. |

Note: BW = Benedict Warner, CS = Christine Sekaggya-Wiltshire, DJS = Derek J Sloan, HRS = Helen R Stagg, IM = Irene Mbabazi, KJH = Katherine J Hill, MS = Marcello Scopazzini, SEM = Sarah E Mills, SM = Stellah Mpagama
